# Supplementary material for: Intra-vector infection dynamics challenge how to model the extrinsic incubation period for major arboviruses: dengue, Zika, and chikungunya
Source: PLoS Comput Biol. 2025 Aug 25;21(8):e1013393. doi: 10.1371/journal.pcbi.1013393 (PMC12440223; doi:10.1371/journal.pcbi.1013393)
Supplement: S1 Fig — (PDF) [file pcbi.1013393.s001.pdf]

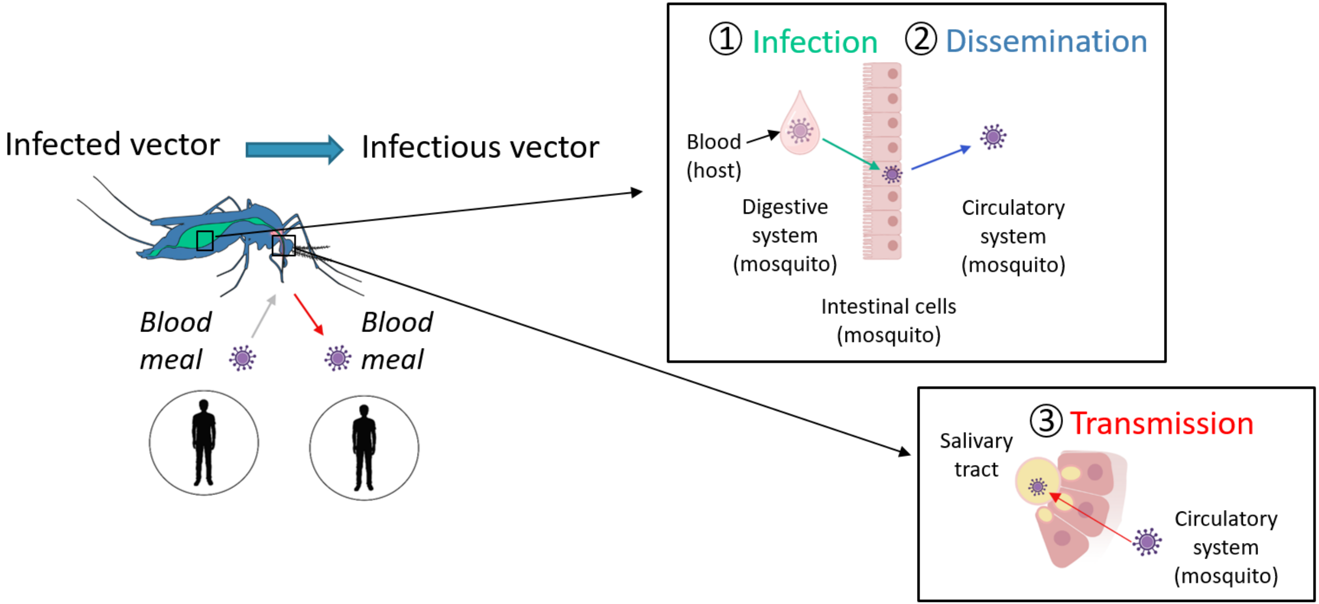

S1 Fig. Intra-vector infection dynamic conceptual diagram. Created in BioRender. https://BioRender.com/d39p227
