# Supplementary material for: Intra-vector infection dynamics challenge how to model the extrinsic incubation period for major arboviruses: dengue, Zika, and chikungunya
Source: PLoS Comput Biol. 2025 Aug 25;21(8):e1013393. doi: 10.1371/journal.pcbi.1013393 (PMC12440223; doi:10.1371/journal.pcbi.1013393)
Supplement: S1 Appendix — This appendix provides additional figures and tables on the experimental data used for IVD model inference. (PDF) [file pcbi.1013393.s002.pdf]

S1 Appendix. Vectorial-competence experimental data

This appendix provides additional figures and tables on the experimental data used for IVD model inference (including a schematic representation of the approach used during the literature search, an overview of the experimental protocol used to obtain these data and a summary of the experimental conditions used for each scenario).

Query :
("arthropod-borne" OR arbovirus)
AND (competence OR ("vector competence") OR ("intra-mosquito") OR ("intra-vector") OR (dissemination) OR (transmission) OR ("systemic infection") OR ("extrinsic incubation period" OR (dynamic))
AND (virus)
AND(("in vivo") OR (empirical) OR (experimental) OR saliva OR ("vector competence assays"))
NOT tick

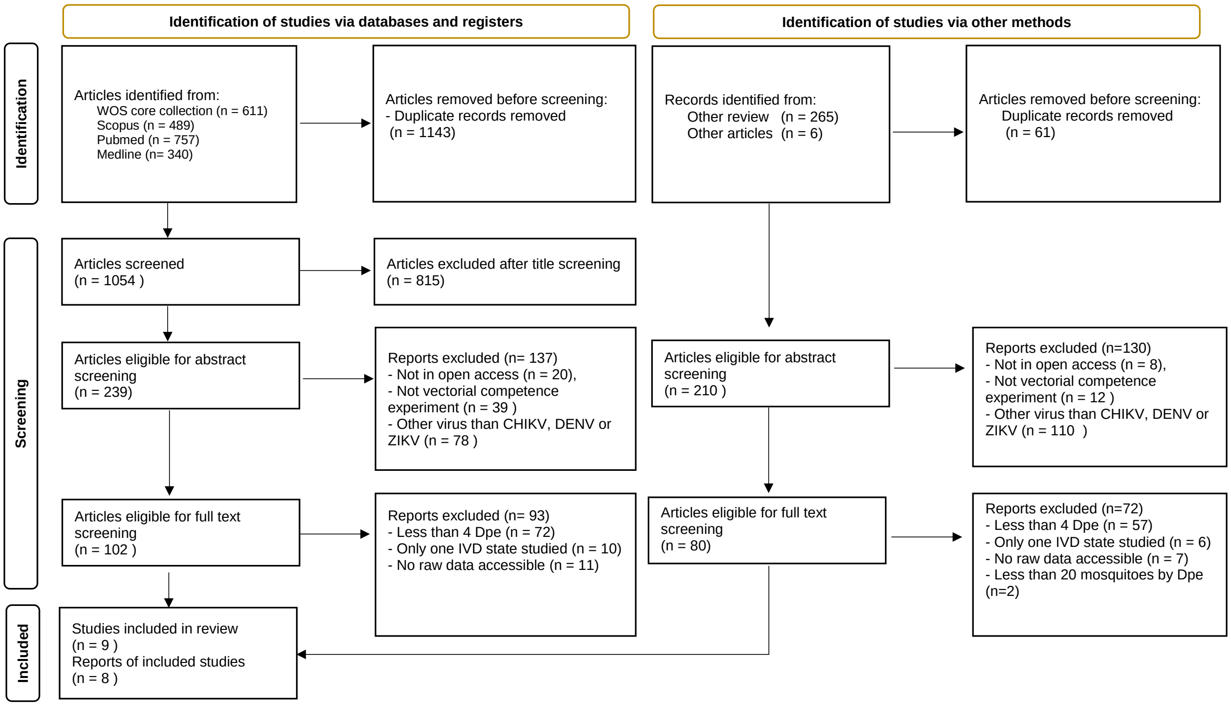

Fig A. Flowchart of extensive literature data searching for inference of IVD model (Source for template: Page MJ, et al. BMJ 2021;372:n71. doi: 10.1136/bmj.n71 (1). This work is licensed under CC BY 4.0. To view a copy of this license, visit https://creativecommons.org/licenses/by/4.0/).

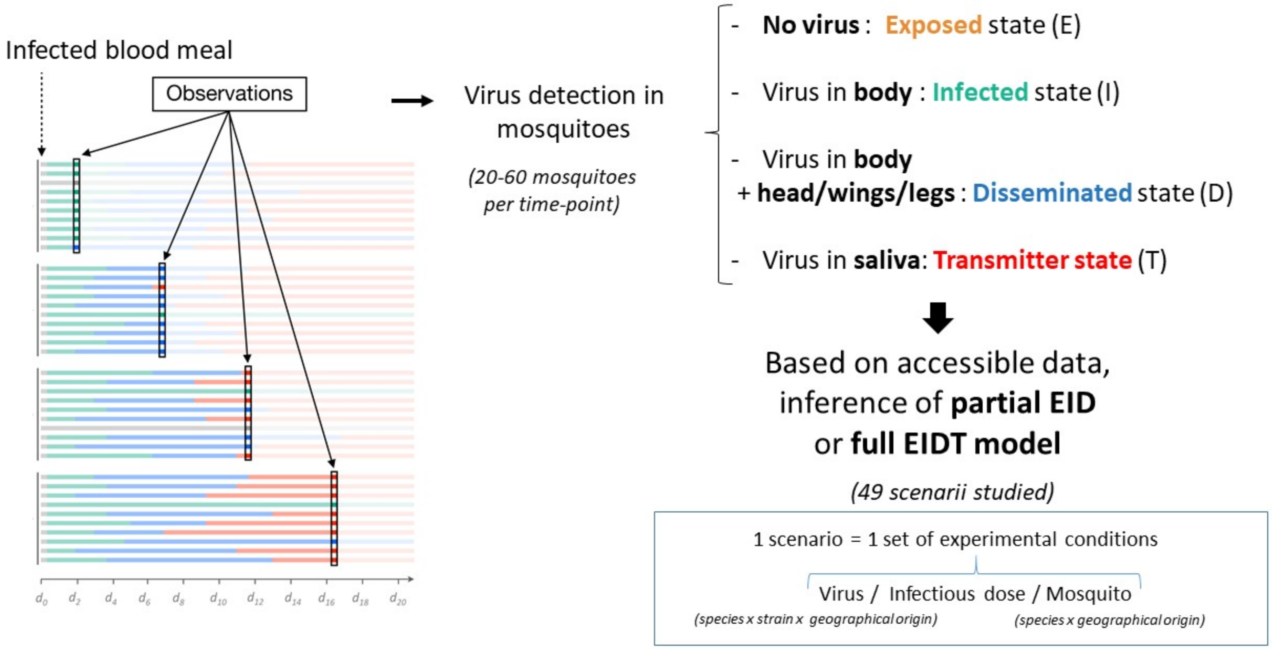

Fig B. Summary of the protocol for the vector competence experiments that provided the data used to infer the model. Each line represents a mosquito killed at a time step depending on the batch in which it is found.Original figure created by the authors.

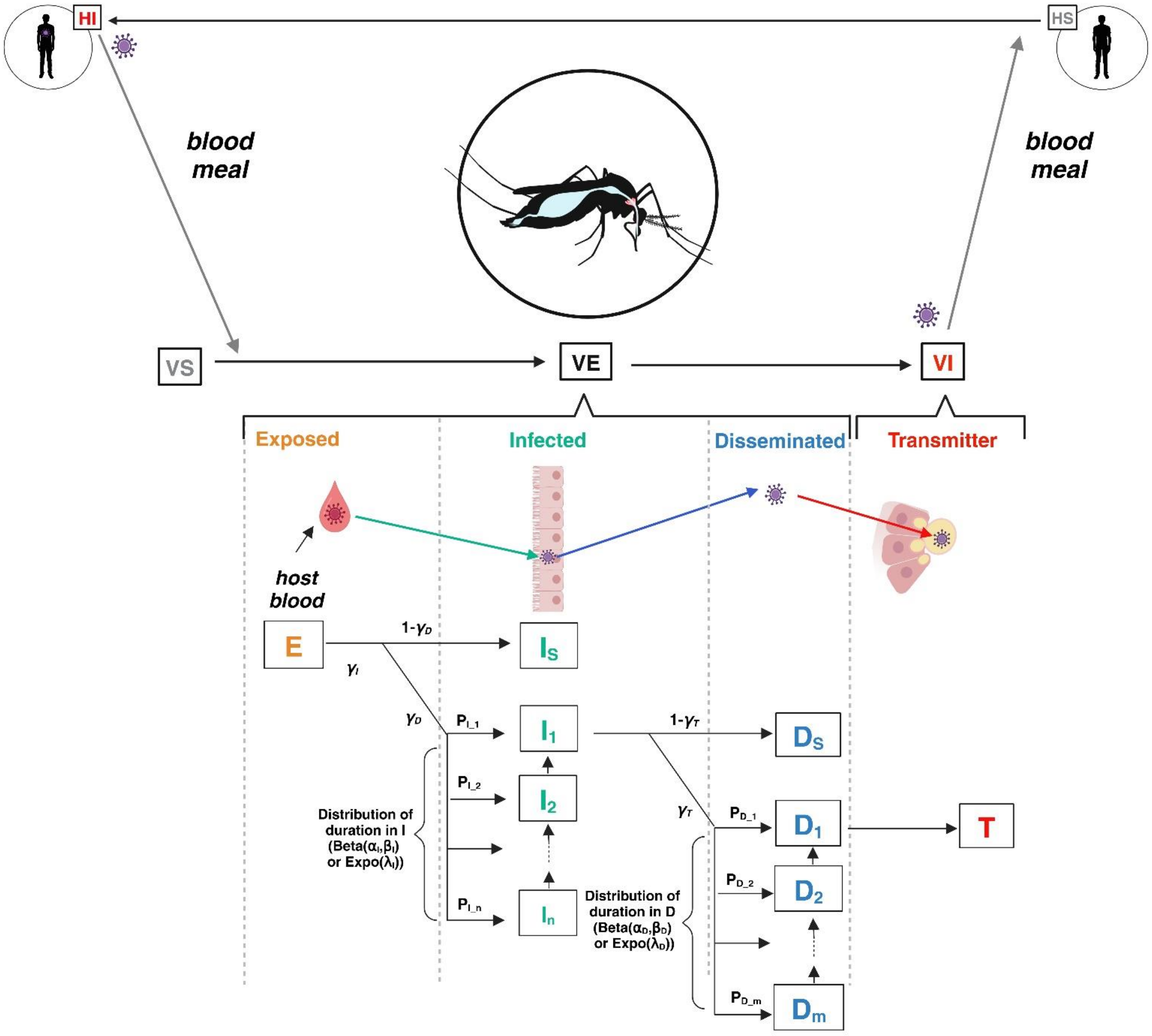

Fig C. Conceptual diagram of the intra-vector infection dynamic (IVD) model couplate with vector-borne disease model transmission. Each compartment represents a state of the vector or host (for IVD model: *E*: exposed vector = vector with virus in its digestive system into blood host, *I*: infected vector = vector with virus in its midgut epithelium, (with I_S: infected vector remaining in *I*, I_1 to I_n: infected vector remaining 1 to n days in *I*), *D*: disseminated vector = vector with virus in its circulatory system, (with D_s: infected vector remaining in *D*, D_1 to D_m: infected vector remaining 1 to *m* days in *D*); *T*: infectious vector = transmitter = vector with virus in its saliva; for vector-host model: *HI*: infectious host, *HS*: healthy host, *VS*: healthy vector, *VE*: exposed vector = vector during extrinsic incubation period, *VI*: infectious vector = transmitter). The model parameters are: γI , γD, γT
(proportion of mosquitoes for which respectively the infection, dissemination and transmission barriers are crossed), α and β (beta law parameters) or λ (exponential law parameters) and *n* and *m*
(respectively maximum length of stay in *I* and *D*), P_{I_1} to P_{I_n}: probability for mosquitoes to be distributed respectively in the I_1 to I_n compartments, P_{D_1} to P_{D_m}: probability for mosquitoes to be distributed respectively in the D_1 to D_m compartments. Created in BioRender. https://BioRender.com/x03h233.

Table A. Summary of experimental data used to infer the partial EID model. CHIKV: chikungunya virus, DENV-1: dengue virus serotype 1, ZIKV: Zika virus, ID: infectious Dose ($\log_{10}$ FFU/mL), FFA: fluorescent focus assay, RT-PCR: reverse transcription polymerase chain reaction, Dpe: day post exposure, ND: not defined, IO: Indian Ocean, AL: Asian lineage, Ly:Lyon, KPP:Kamphaeng Phet Province, Mar: Marseille, RI: Reunion Island.

Virus species	Virus strain (origin)	Mosquito genus and species (origin)	ID $\log_{10}$	Ambiance conditions: Temperature (℃) Humidity (%) Light-dark cycle (hours)	Dpe	Mosquito number (mean by Dpe)	Mosquitoes parts analyzed for infection (I) / dissemination (D) and method used	Scenario	Ref
CHIKV	strain 06.21 (IO)	*Ae.albopictus* (France, Ly)	3.94 (FFU/mL)	26℃ /70% /ND	2,6,9,14	43	I:body (FFA)/ D : head (FFA)	CHIKVp1	(2)
CHIKV	strain 06.21 (IO)	*Ae.albopictus* (France, Ly)	6.07 (FFU/mL)	26℃ /70% /ND	2,6,9,14	31	I:body (FFA)/ D : head (FFA)	CHIKVp2	(2)
CHIKV	strain 06.21 (IO)	*Ae.albopictus* (France, Ly)	8.63 (FFU/mL)	26℃ /70% /ND	2,6,9,14	19	I:body (FFA)/ D : head (FFA)	CHIKVp3	(2)
DENV-1	Thailand 2010 a	*Ae.aegypti* (Thailand, KPP)	5.74 (FFU/mL)	ND/ND/ND	4,6,8,12,18	16	I:body (FFA)/ D : head (FFA)	DENVp1	(3)
DENV-1	Thailand 2010b	*Ae.aegypti* (Thailand, KPP)	5.70 (FFU/mL)	ND/ND/ND	4,6,8,12,18	21	I:body (FFA)/ D : head (FFA)	DENVp2	(3)
DENV-1	Thailand 2013	*Ae.aegypti* (Thailand, KPP)	5.79 (FFU/mL)	ND/ND/ND	4,6,8,12,18	17	I:body (FFA)/ D : head (FFA)	DENVp3	(3)
DENV-1	Laos 2012	*Ae.aegypti* (Thailand, KPP)	5.84 (FFU/mL)	ND/ND/ND	4,6,8,12,18	25	I:body (FFA)/ D : head (FFA)	DENVp4	(3)
DENV-1	N.Caledonia 2013	*Ae.aegypti* (Thailand, KPP)	5.77 (FFU/mL)	ND/ND/ND	4,6,8,12,18	17	I:body (FFA)/ D : head (FFA)	DENVp5	(3)
DENV-1	Gabon 2012	*Ae.aegypti* (Thailand, KPP)	5.82 (FFU/mL)	ND/ND/ND	4,6,8,12,18	15	I:body (FFA)/ D : head (FFA)	DENVp6	(3)
DENV-1	Haiti 2012	*Ae.aegypti* (Thailand, KPP)	5.81 (FFU/mL)	ND/ND/ND	4,6,8,12,18	19	I:body (FFA)/ D : head (FFA)	DENVp7	(3)
DENV-1	Thailand 2012	*Ae.aegypti* (Thailand, KPP)	5.80 (FFU/mL)	ND/ND/ND	4,6,8,12,18	19	I:body (FFA)/ D : head (FFA)	DENVp8	(3)
ZIKV	strain SL1602 (AL)	*Ae.albopictus* (France, Mar)	6.48 (FFU/mL)	28℃ / 75 +/-5%/16h:8h	5,10,14,17,21	26	I:body(RT-PCR)/D:head(RT-PCR)	ZIKVp1	(4)
ZIKV	strain SL1602 (AL)	*Ae.albopictus* (France, Mar)	6.87 (FFU/mL)	28℃ / 75 +/-5%/16h:8h	5,10,14,17,21	19	I:body(RT-PCR)/D:head(RT-PCR)	ZIKVp2	(4)
ZIKV	strain SL1602 (AL)	*Ae.albopictus* (France, RI)	5.90 (FFU/mL)	28℃ / 75 +/-5%/16h:8h	5,10,14,17,21	23	I:body(RT-PCR)/D:head(RT-PCR)	ZIKVp3	(4)
ZIKV	strain SL1602 (AL)	*Ae.albopictus* (France, RI)	6.87 (FFU/mL)	28℃ / 75 +/-5%/16h:8h	5,10,14,17,21	23	I:body(RT-PCR)/D:head(RT-PCR)	ZIKVp4	(4)
ZIKV	strain SL1602 (AL)	*Ae.albopictus* (France, RI)	8.37 (FFU/mL)	28℃ / 75 +/-5%/16h:8h	5,10,14,17,21	21	I:body(RT-PCR)/D:head(RT-PCR)	ZIKVp5	(4)
ZIKV	strain SL1602 (AL)	*Ae.albopictus* (France, RI)	6.48 (FFU/mL)	28℃ / 75 +/-5%/16h:8h	10,14,17,21	13	I:body(RT-PCR)/D:head(RT-PCR)	ZIKVp6	(4)

Table B. Summary of experimental data used for CHIKV to infer the complete EIDT model. CHIKV: chikungunya virus, Car: Carthage, Ami: Amilcar, La Mar: La Marsa, Mad Island: Madeira Island, P do mar: Paul do Mar, ID: infectious dose, FFA: focus forming assay, PFA: plaque forming assay, PFU: plaque forming unit, FFU: focus forming unit, RT-PCR: reverse transcription polymerase chain reaction, Dpe: day post exposure, ND: not defined

Virus species	Virus strain (origin)	Mosquito genus<br>and<br>species (origin)	ID $\log_{10}$	Temperature (℃)<br>Humidity (%)<br>Light-dark cycle (h)	Dpe	Mosquito<br>number<br>(mean by Dpe)	Mosquitoes parts<br>analyzed (method)	Scenario	Ref
CHIKV	06.21<br>(Reunion Island)	*Ae.albopictus*<br>(Morocco, Rabat)	7 (PFU/mL)	28 +/- 1℃<br>80%<br>16h:8h	3,7,14,21	30	I:abdomen/thorax (FFA)<br>D:head (FFA)<br>T:saliva (FFA)	CHIKVc1	(5)
CHIKV	Carribean strain<br>(French carribean Island)	*Ae.aegypti*<br>(Thailand, Kamphaeng Phet)	6 (PFU/mL)	28℃<br>70%<br>12h:12h	3,6,9,12	48	I:body(PFA)<br>D:head(PFA)<br>saliva(PFA)	CHIKVc2	(6)
CHIKV	Carribean strain-2013<br>(Carribean Island)	*Ae.aegypti*<br>(French Guiana, Royal Islands)	6 (PFU/mL)	28 +/- 2℃<br>80% +/- 10%<br>12h:12h	3,5,7,10,14	41	I:midgut (RT-PCR)<br>I:head(RT-PCR)<br>T:saliva(RT-PCR)	CHIKVc3	(7)
CHIKV	06.21<br>(India)	*Ae.geniculatus*<br>(Albania, Tirana)	8 (FFU/mL)	28 +/-1℃<br>80%<br>16h:8h	3,5,7,10,12,14,20	19	I:body(FFA)<br>D:head(FFA)<br>T:saliva(FFA)	CHIKVc4	(8)
CHIKV	PF14/300914-109<br>(Tahiti)	*Ae.aegypti*<br>(Tahiti island, Toahotu)	7 (TCID50/mL)	27℃<br>80%<br>12h:12h	6,9,14,21	39	I:thorax/abdomen (RT-PCR)<br>D:legs (RT-PCR)<br>T:saliva (FFA)	CHIKVc5	(9)
CHIKV	06.21<br>(Reunion Island)	*Ae.albopictus*<br>(Tunisia,Car, Ami, La Mar)	7 (FFU/mL)	28 +/- 1℃<br>80%<br>16h:8h	3,7,10,14,21	27	I:abdomen (FFA)<br>D:thorax/head (FFA)<br>T:saliva (FFA)	CHIKVc6	(10)
CHIKV	06.21<br>(India)	*Ae.albopictus*<br>(Albania, Tirana)	8 (FFU/mL)	28 +/-1℃<br>80%<br>16h:8h	3,5,7,10,12,14,20	18	I:body(FFA)<br>D:head(FFA)<br>T:saliva(FFA)	CHIKVc7	(8)
CHIKV	NC/2011-568<br>(New Caledonia)	*Ae.aegypti*<br>(Mad Island, Funchal)	7.3 (FFU/mL)	28 +/- 1℃<br>80%<br>ND	3,6,9,14	20	I:thorax/abdomen (ND)<br>I:head(FFA)<br>T:saliva(FFA)	CHIKVc8	(11)
CHIKV	NC/2011-568<br>(New Caledonia)	*Ae.aegypti*<br>(Mad Island, P. do Mar)	7.3 (FFU/mL)	28 +/- 1℃<br>80%<br>ND	3,6,9,14	20	I:thorax/abdomen (ND)<br>I:head(FFA)<br>T:saliva(FFA)	CHIKVc9	(11)
CHIKV	99659<br>(British Virgin Islands)	*Ae.aegypti*<br>(Mexico, Poza Rica)	6.9 (PFU/mL)	28℃<br>70-80%<br>12h:12h	2,4,6,8,10,12,14,<br>16,18,20	60	I:midgut(RT-PCR)<br>D:legs/wings (RT-PCR)<br>T:saliva (PFA)	CHIKVc10	(12)

Table C. Summary of experimental data used for DENV to infer the complete EIDT model. DENV-1: dengue virus serotype 1, DENV-2: dengue virus serotype 2, DENV-3: dengue virus serotype 3, Car: Carthage, Ami: Amilcar, La Mar: La Marsa, ID: infectious dose, FFA: focus forming assay, PFA:plaque forming assay, PFU: plaque forming unit, FFU: focus forming unit, RT-PCR: reverse transcription polymerase chain reaction, Dpe: day post exposure, ND: not defined

Virus species	Virus strain (origin)	Mosquito genus and species (origin)	ID $\log_{10}$	Temperature (℃) Humidity (%) Light-dark cycle (h)	Dpe	Mosquito number (mean by Dpe)	Mosquitoes parts analyzed (method)	Scenario	Ref
DENV-2	Prof Leon Rosen (Thailand, Bangkok)	*Ae.albopictus* (Morocco, Rabat)	7 (FFU/mL)	28 +/- 1℃<br>80%<br>16h:8h	3,7,14,21	28	I:abdomen/thorax (FFA)<br>D:head (FFA)<br>T:saliva (FFA)	DENVc1	(5)
DENV-2	(Thailand, Bangkok)	*Ae.albopictus* (Tunisia,Car, Ami, La Mar)	7 (TCID50/mL)	28 +/- 1℃<br>80%<br>16h:8h	3,7,10,14,21	28	I:abdomen (FFA)<br>D:thorax/head (FFA)<br>T:saliva (FFA)	DENVc2	(10)
DENV-1	SG (EHI)D1/30889Y14 (Singapore)	*Ae.albopictus* (Italia, Reynosa)	5 (PFU/mL)	ND<br>ND<br>ND	7,14,21,28	17	I:body(PFA)<br>D:legs/wings(PFA)<br>T:saliva (PFA)	DENVc3	(13)
DENV-3	1998- GenBank JN406514 (Cairns)	*Ae.aegypti* (Australia,Cairns)	4.9 (CCID50/mosquito)	28℃<br>80%<br>12h:12h	2,3,4,5,6,7,10,14	21	I:body(ELISA)<br>D:legs/wings (ELISA)<br>T:saliva(ELISA)	DENVc4	(14)
DENV-3	2008a - GenBank JN406515 (Cairns)	*Ae.aegypti* (Australia,Cairns)	5.1(CCID50/mosquito)	28℃<br>75%<br>12h:12h	2,3,4,5,6,7,10,14	21	I:body(ELISA)<br>D:legs/wings (ELISA)<br>T:saliva(ELISA)	DENVc5	(14)
DENV-1	SG (EHI)D1/30889Y14 (Singapore)	*Ae.albopictus* (Italia, Rome)	5 (PFU/mL)	ND<br>ND<br>ND	7,14,21,28	18	I:body(PFA)<br>D:legs/wings(PFA)<br>T:saliva (PFA)	DENVc6	(13)
DENV-1	SG (EHI)D1/30889Y14 (Singapore)	*Ae.albopictus* (Italia, Montecchio)	5 (PFU/mL)	ND<br>ND<br>ND	7,14,21,28	18	I:body(PFA)<br>D:legs/wings(PFA)<br>T:saliva (PFA)	DENVc7	(13)

Table D. Summary of experimental data used for ZIKV to infer the complete EIDT model. ZIKV: Zika virus, Car: Carthage, Ami: Amilcar, La Mar: La Marsa, ID: infectious dose, FFA: focus forming assay, PFA: plaque forming assay, PFU: plaque forming unit, FFU: focus forming unit, RT-PCR: reverse transcription polymerase chain reaction, Dpe: day post exposure, ND: not defined, HRH: high relative humidity

Virus species	Virus strain (origin)	Mosquito genus and species (origin)	ID $\log_{10}$	Temperature (℃) Humidity (%) Light-dark cycle (h)	Dpe	Mosquito number (mean by Dpe)	Mosquitoes parts analyzed (method)	Scenario	Ref
ZIKV	PRVABC59 (United States Puerto Rico)	*Ae.aegypti* (Mexico, Poza Rica)	7.2 (PFU/mL)	28℃ 70-80% 12h:12h	2,4,6,8,10,12,14, 16,18,20	30	I:midgut(RT-PCR) D:legs/wings (RT-PCR) T:saliva (PFA)	ZIKVc1	(12)
ZIKV	ZIKV strain PF13/251013-18 (French Polynesia)	*Ae.aegypti* (Tahiti island, Toahotu)	6.8 (TCID50/mL)	27℃ 0,8 12h:12h	6,9,14,21	40	-I : abdomen/thorax (RT-PCR) D: legs(RT-PCR) T : saliva(FFA)	ZIKVc2	(15)
ZIKV	MR 766 (Uganda)	*Ae.aegypti* (Australia,Townsville)	6.5(TCID50/mL)	28℃ HRH 12h:12h	5,7,10,14	24	I:body (RT-PCR) D:legs/wings (RT-PCR) T:saliva (RT-PCR)	ZIKVc3	(16)
ZIKV	NC-2014-5132 (New Caledonia, 2014)	*Ae.aegypti* (French Polynesia)	7 (TCID50/mL)	28℃ 80% 12h:12h	6,9,14,21	32	I:abdomen/thorax (PFA) D:head PFA) T:saliva (PFA)	ZIKVc4	(17)
ZIKV	NC-2014-5132 (New Caledonia,2014)	*Ae.polynesiensis* (Wallis)	7 (TCID50/mL)	28℃ 80% 12h:12h	6,9,14,21	38	I:abdomen/thorax (PFA) D:head PFA) T:saliva (PFA)	ZIKVc5	(17)
ZIKV	NC-2014-5132 (New Caledonia,2014)	*Ae.albopictus* (Morocco, Rabat)	7.2 (PFU/mL)	28 +/- 1℃ 80% 16h:8h	3,7,14,21	28	I: abdomen/thorax (PFA) D:head (PFA) T:saliva (PFA)	ZIKVc6	(5)
ZIKV	NC-2014-5132 (New Caledonia)	*Ae.aegypti* (Samoa)	7 (TCID50/mL)	28℃ 80% 12h:12h	6,9,14,21	39	I:abdomen/thorax (PFA) D:head PFA) T:saliva (PFA)	ZIKVc7	(17)
ZIKV	NC-2014-5132 (New Caledonia,2014)	*Ae.aegypti* (New Caledonia)	7 (TCID50/mL)	28℃ 80% 12h:12h	6,9,14,21	26	I:abdomen/thorax (PFA) D:head PFA) T:saliva (PFA)	ZIKVc8	(17)
ZIKV	NC-2014-5132 (New Caledonia,2014)	*Ae.albopictus* (Tunisia,Car, Ami, La Mar)	7 (TCID50/mL)	28 +/- 1℃ 80% 16h:8h	7,10,14,21	23	I:abdomen (PFA) D:thorax/head (PFA) T:saliva (PFA)	ZIKVc9	(10)
ZIKV	SA-2016-18246 (French Guiana)	*Ae.aegypti* (New Caledonia)	7 (TCID50/mL)	28℃ 80% 12h:12h	6,9,14,21	29	I:abdomen/thorax (PFA) D:head PFA) T:saliva (PFA)	ZIKVc10	(18)
ZIKV	AF-1991-HD78788 (Senegal, 1991)	*Ae.aegypti* (New Caledonia)	7 (TCID50/mL)	28℃ 80% 12h:12h	6,9,14,21	28	I:abdomen/thorax (PFA) D:head PFA) T:saliva (PFA)	ZIKVc11	(18)
ZIKV	NC-2014-843 (New Caledonia, 2014)	*Ae.aegypti* (New Caledonia)	7 (TCID50/mL)	28℃ 80% 12h:12h	6,9,14,21	30	I:abdomen/thorax (PFA) D:head PFA) T:saliva (PFA)	ZIKVc12	(18)
ZIKV	NC-2015-2391 (New Caledonia, 2015)	*Ae.aegypti* (New Caledonia)	7 (TCID50/mL)	28℃ 80% 12h:12h	6,9,14,21	34	I:abdomen/thorax (PFA) D:head PFA) T:saliva (PFA)	ZIKVc13	(18)
ZIKV	AF-2002-ArD 165 522 (Senegal, 2002)	*Ae.aegypti* (New Caledonia)	7 (TCID50/mL)	28℃ 80% 12h:12h	6,9,14,21	30	I:abdomen/thorax (PFA) D:head PFA) T:saliva (PFA)	ZIKVc14	(18)
ZIKV	NC-2014-5132 (New Caledonia)	*Ae.polynesiensis* (French Polynesia)	7 (TCID50/mL)	28℃ 80% 12h:12h	6,9,14,21	30	I:abdomen/thorax (PFA) D:head PFA) T:saliva (PFA)	ZIKVc15	(17)

References

1. MJ Page, et al., The PRISMA 2020 statement: an updated guideline for reporting systematic reviews. *BMJ (Clinical research ed.)* **372**, n71 (2021).
2. B Viginier, et al., Chikungunya intra-vector dynamics in Aedes albopictus from Lyon (France) upon exposure to a human viremia-like dose range reveals vector barrier's permissiveness and supports local epidemic potential. *Peer Community J.* **3**, e96 (2023).
3. A Fontaine, et al., Epidemiological significance of dengue virus genetic variation in mosquito infection dynamics. *PLOS Pathog.* **14**, e1007187 (2018) lecture totale annotée.
4. S Lequime, et al., Modeling intra-mosquito dynamics of Zika virus and its dose-dependence confirms the low epidemic potential of Aedes albopictus. *PLOS Pathog.* **16**, e1009068 (2020) lecture totale annotée.
5. F Amraoui, et al., Potential of Aedes albopictus to cause the emergence of arboviruses in Morocco. *PLOS Neglected Trop. Dis.* **13**, e0006997 (2019).
6. F Merwaiss, et al., Chikungunya Virus Replication Rate Determines the Capacity of Crossing Tissue Barriers in Mosquitoes. *J. Virol.* **95**, e01956–20 (2021).
7. L Wang, et al., Interactions between vector competence to chikungunya virus and resistance to deltamethrin in Aedes aegypti laboratory lines? *Med. Vet. Entomol.* **36**, 486–495 (2022) _eprint: https://onlinelibrary.wiley.com/doi/pdf/10.1111/mve.12593.
8. J Prudhomme, et al., The native European Aedes geniculatus mosquito species can transmit chikungunya virus. *Emerg. Microbes & Infect.* **8**, 962–972 (2019).
9. V Richard, T Paoaafaite, VM Cao-Lormeau, Vector Competence of Aedes aegypti and Aedes polynesiensis Populations from French Polynesia for Chikungunya Virus. *PLoS Neglected Trop. Dis.* **10**, e0004694 (2016).
10. C Bohers, et al., The recently introduced Aedes albopictus in Tunisia has the potential to transmit chikungunya, dengue and Zika viruses. *PLoS Neglected Trop. Dis.* **14**, e0008475 (2020).
11. G Seixas, et al., Potential of Aedes aegypti populations in Madeira Island to transmit dengue and chikungunya viruses. *Parasites & Vectors* **11**, 509 (2018).
12. A Robison, MC Young, AD Byas, C Rückert, GD Ebel, Comparison of Chikungunya Virus and Zika Virus Replication and Transmission Dynamics in Aedes aegypti Mosquitoes. *The Am. J. Trop. Medicine Hyg.* **103**, 869–875 (2020).
13. C Fortuna, et al., Assessing the Risk of Dengue Virus Local Transmission: Study on Vector Competence of Italian Aedes albopictus. *Viruses* **16**, 176 (2024) Number: 2 Publisher: Multidisciplinary Digital Publishing Institute.
14. SA Ritchie, et al., An Explosive Epidemic of DENV-3 in Cairns, Australia. *PLoS ONE* **8**, e68137 (2013).
15. V Richard, T Paoaafaite, VM Cao-Lormeau, Vector Competence of French Polynesian Aedes aegypti and Aedes polynesiensis for Zika Virus. *PLoS Neglected Trop. Dis.* **10**, e0005024 (2016).
16. S Hall-Mendelin, et al., Assessment of Local Mosquito Species Incriminates Aedes aegypti as the Potential Vector of Zika Virus in Australia. *PLoS Neglected Trop. Dis.* **10**, e0004959 (2016).
17. E Calvez, et al., Zika virus outbreak in the Pacific: Vector competence of regional vectors. *PLoS Neglected Trop. Dis.* **12**, e0006637 (2018).
18. E Calvez, et al., Differential transmission of Asian and African Zika virus lineages by Aedes aegypti from New Caledonia. *Emerg. Microbes & Infect.* **7**, 159 (2018).
