# Supplementary material for: Intra-vector infection dynamics challenge how to model the extrinsic incubation period for major arboviruses: dengue, Zika, and chikungunya
Source: PLoS Comput Biol. 2025 Aug 25;21(8):e1013393. doi: 10.1371/journal.pcbi.1013393 (PMC12440223; doi:10.1371/journal.pcbi.1013393)
Supplement: S2 Appendix — This appendix provides additional figures showing the results obtained from datasets that used the partial EID model. Additionally, for each scenario, figures are provided that include the main selected distributions, as well as the main selected dynamics in the different IVD stages. (PDF) [file pcbi.1013393.s003.pdf]

S2 Appendix. Figures of additional results

This appendix provides additional figures of results obtain for datasets requiring to use the partial EID model (same figures as those in the main article for EIDT model). Additionally, for each scenario, figures are provided that include the main selected distributions, as well as the main selected dynamics in the different IVD stages.

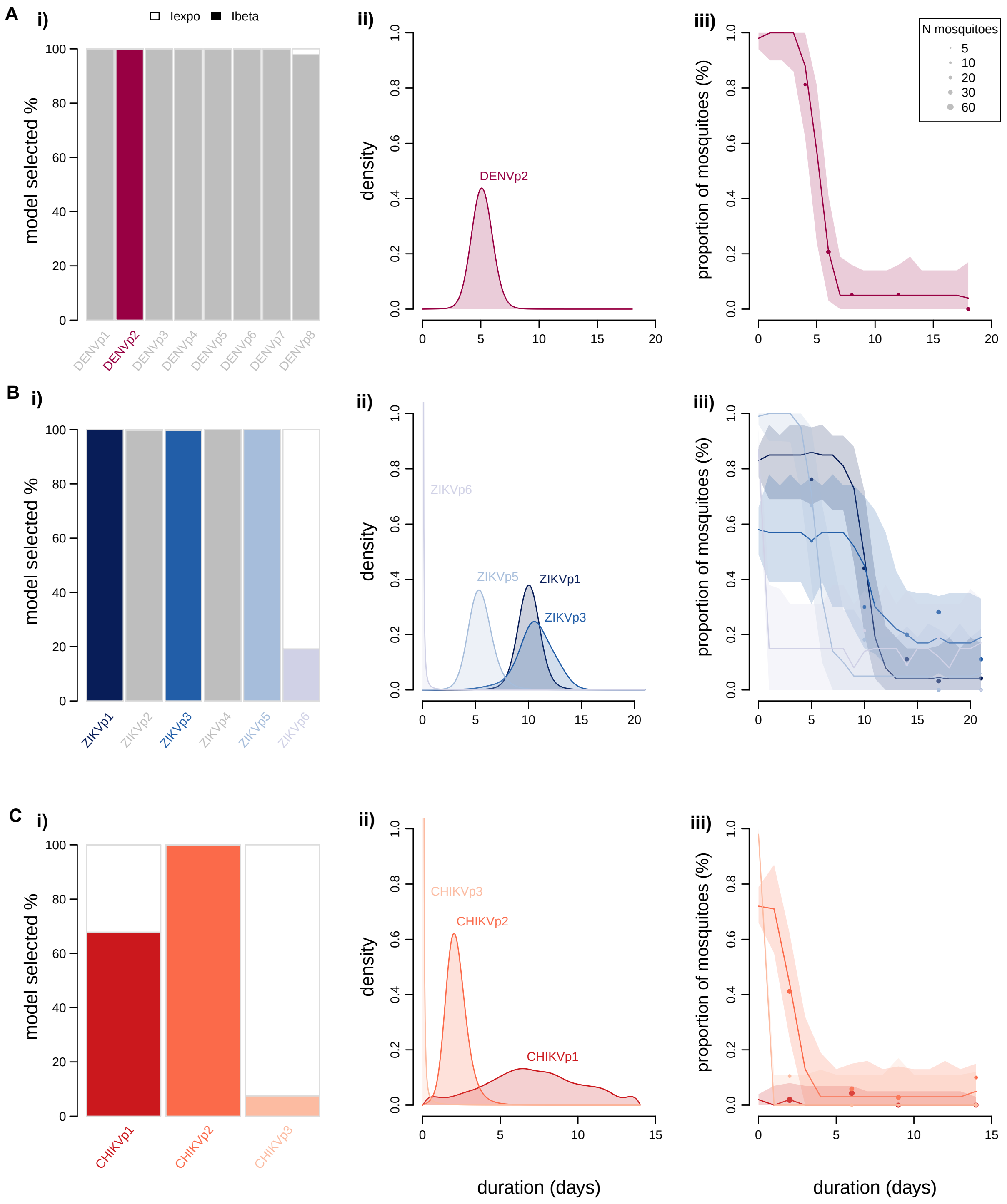

Fig A. Inference results for IVD stages distributions for all scenarios tested for DENV (A), ZIKV (B) and CHIKV (C) with EID model: i) Selected proportion of each model (modBeta, modExpo,) for each scenario with one colour per scenario. ii) Average of the selected distributions in the infected state for the main model selected (only selected scenarios* are represented). iii) Selected dynamics in the infected state for the main model selected (only selected scenarios* are represented). Dots represent observed data, line mean dynamics and uncertainty ribbons (5%-95%) represent selected simulated dynamics for each scenario.* *scenarios with 5 or more observed day post exposure*

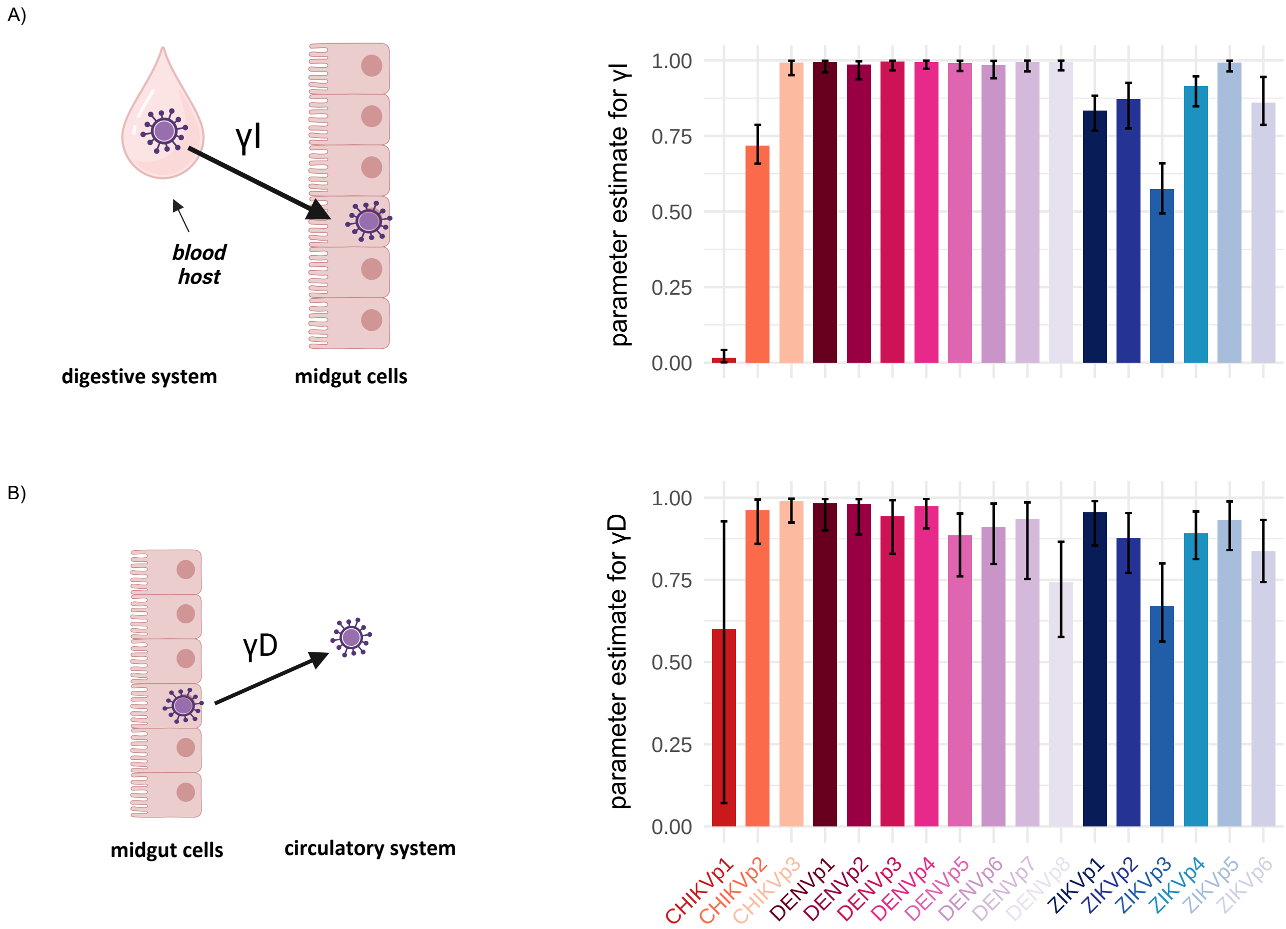

Fig B. Parameter estimates for the probability of crossing the infection and dissemination barriers for all scenarios* tested for chikungunya virus (CHIKV), dengue virus (DENV) and Zika virus (ZIKV). A: Infection barrier; B: Dissemination barrier. Each bar represents the parameter mode for a given scenario, with one colour per scenario and with the 90% credibility interval represented by the error bar. **One scenario consists of a vector competence experiment on female mosquitoes from a specific genus, species and geographical origin, exposed to a virus isolate from a given species at a single infectious dose (log10 FFU/mL) in the blood meal.* Created in BioRender. https://BioRender.com/v27z184

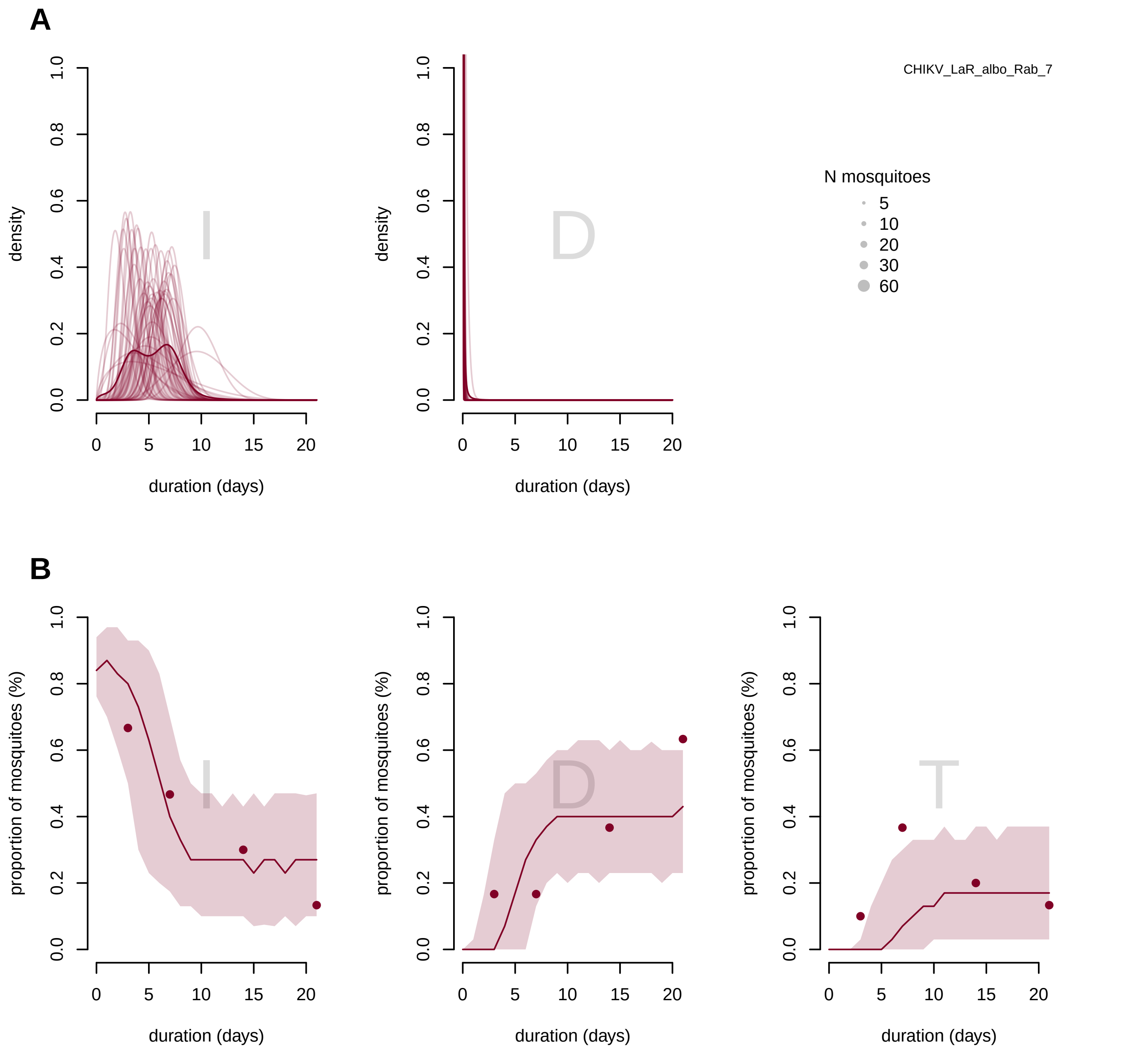

Fig C. Inference results for IVD stages distributions for scenario CHIKVc1(CHIKV_LaR_albo_Rab_7): *Aedes.albopictus* from Rabat infected by chikungunya virus from Reunion Island with an infectious dose of 7 $\log_{10}$ PFU/mL : A) Selected distributions in the infected and disseminated states for the main model selected. The dark line represents the mean of distributions and light lines represent a random sample of 50 distribution among all selected distribution. B) Selected dynamics in the infected (I), disseminated (D), and transmitter (T) states for the main model selected. The dots represent the observed data, the line (mean dynamics), and the uncertainty ribbons (5%-95%) represent selected simulated dynamic.

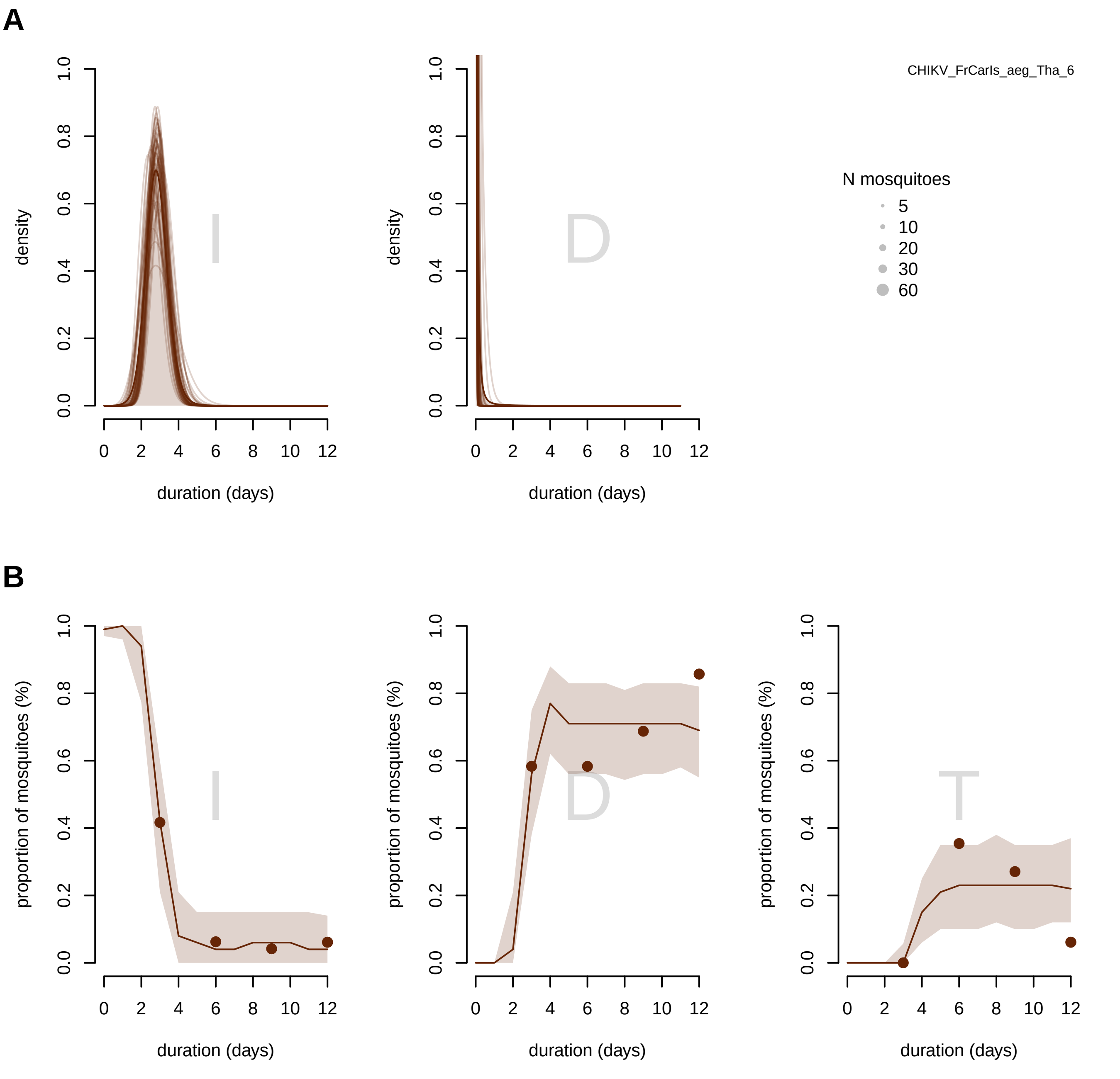

Fig D. Inference results for IVD stages distributions for scenario CHIKVc2(CHIKV_FrCarIs_aeg_Tha_6): *Aedes.aegypti* from Thailand infected by chikungunya virus from French Caribbean Island with an infectious dose of 6 $\log_{10}$ PFU/mL : A) Selected distributions in the infected and disseminated states for the main model selected. The dark line represents the mean of distributions and light lines represent a random sample of 50 distribution among all selected distribution. B) Selected dynamics in the infected (I), disseminated (D), and transmitter (T) states for the main model selected. The dots represent the observed data, the line (mean dynamics), and the uncertainty ribbons (5%-95%) represent selected simulated dynamic.

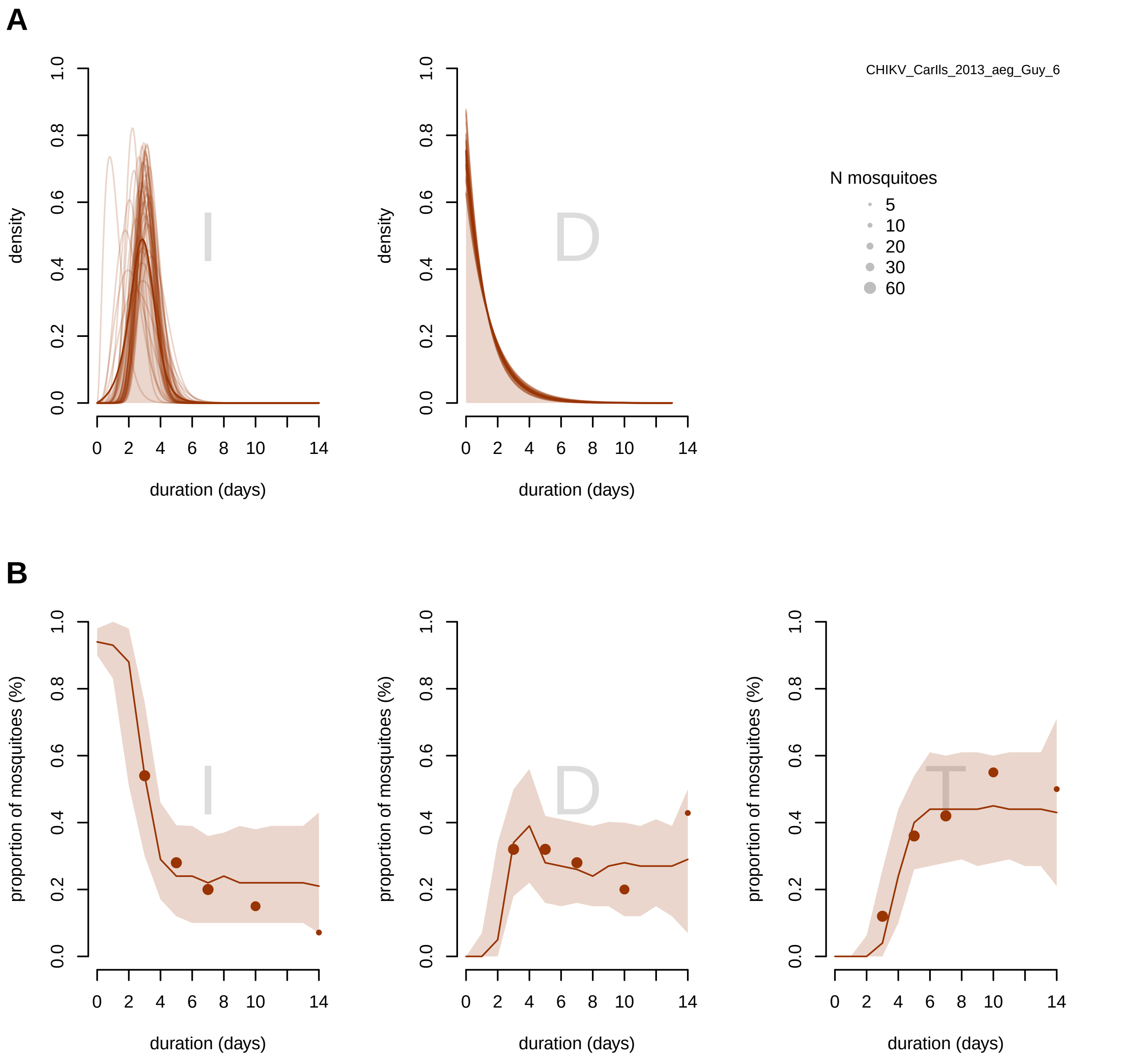

Fig E. Inference results for IVD stages distributions for scenario CHIKVc3(CHIKV_CarIls_2013_aeg_Guy_6): *Aedes.aegypti* from Guyana infected by chikungunya virus from Caribbean Island (2013) with an infectious dose of 6 $\log_{10}$ PFU/mL : A) Selected distributions in the infected and disseminated states for the main model selected. The dark line represents the mean of distributions and light lines represent a random sample of 50 distribution among all selected distribution. B) Selected dynamics in the infected (I), disseminated (D), and transmitter (T) states for the main model selected. The dots represent the observed data, the line (mean dynamics), and the uncertainty ribbons (5%-95%) represent selected simulated dynamic.

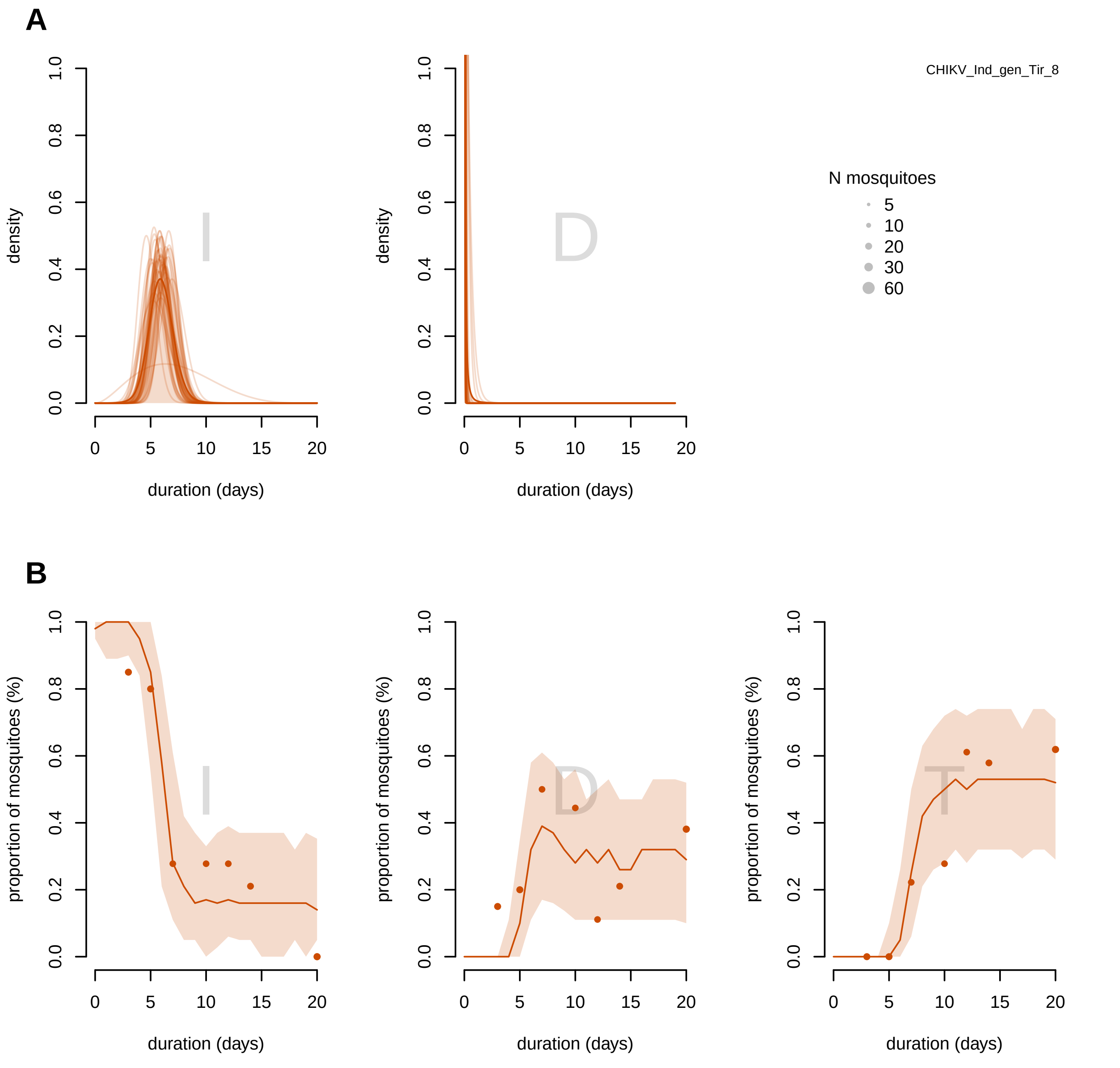

Fig F. Inference results for IVD stages distributions for scenario CHIKVc4(CHIKV_Ind_gen_Tir_8): *Aedes.geniculatus* from Tirana infected by chikungunya virus from India with an infectious dose of 8 $\log_{10}$ FFU/mL : A) Selected distributions in the infected and disseminated states for the main model selected. The dark line represents the mean of distributions and light lines represent a random sample of 50 distribution among all selected distribution. B) Selected dynamics in the infected (I), disseminated (D), and transmitter (T) states for the main model selected. The dots represent the observed data, the line (mean dynamics), and the uncertainty ribbons (5%-95%) represent selected simulated dynamic.

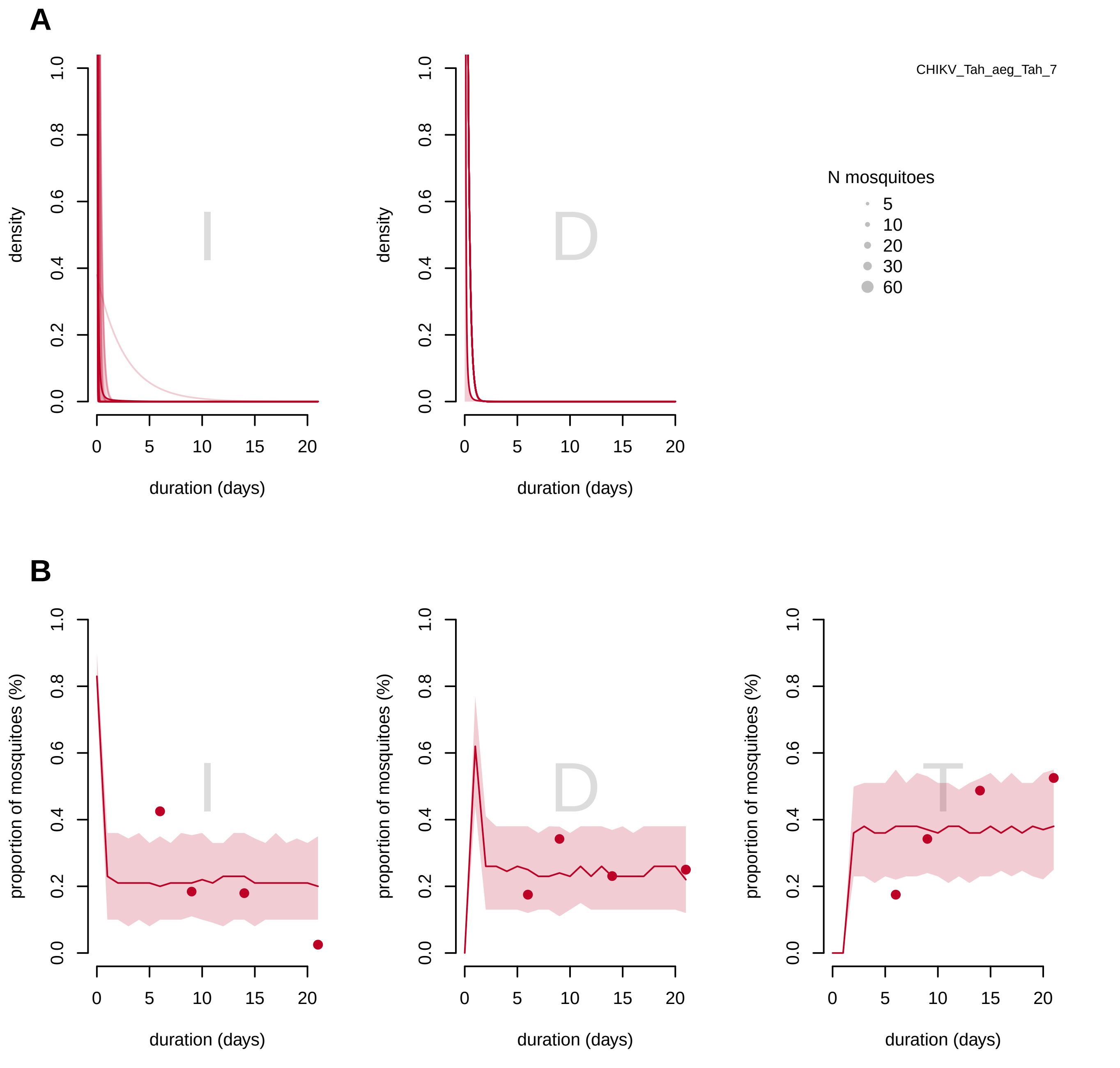

Fig G. Inference results for IVD stages distributions for scenario CHIKVc5(CHIKV_Tah_aeg_Tah_7): *Aedes.aegypti* from Tahiti infected by chikungunya virus from Tahiti with an infectious dose of 7 $\log_{10}$ TCID50/mL : A) Selected distributions in the infected and disseminated states for the main model selected. The dark line represents the mean of distributions and light lines represent a random sample of 50 distribution among all selected distribution. B) Selected dynamics in the infected (I), disseminated (D), and transmitter (T) states for the main model selected. The dots represent the observed data, the line (mean dynamics), and the uncertainty ribbons (5%-95%) represent selected simulated dynamic.

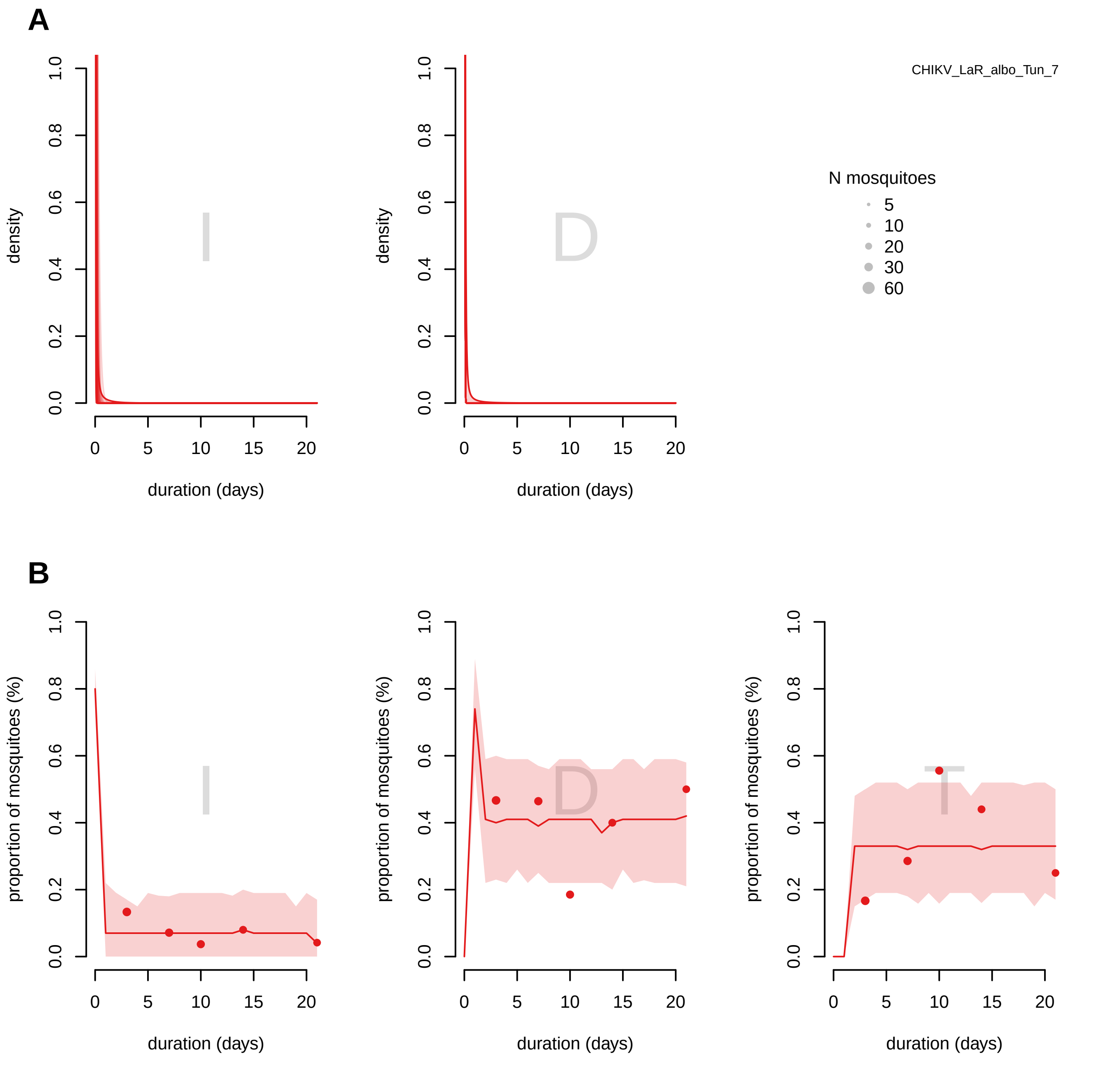

Fig H. Inference results for IVD stages distributions for scenario CHIKVc6(CHIKV_LaR_albo_Tun_7): *Aedes.albopictus* from Tunisia infected by chikungunya virus from Reunion Island with an infectious dose of 7 $\log_{10}$ FFU/mL : A) Selected distributions in the infected and disseminated states for the main model selected. The dark line represents the mean of distributions and light lines represent a random sample of 50 distribution among all selected distribution. B) Selected dynamics in the infected (I), disseminated (D), and transmitter (T) states for the main model selected. The dots represent the observed data, the line (mean dynamics), and the uncertainty ribbons (5%-95%) represent selected simulated dynamic.

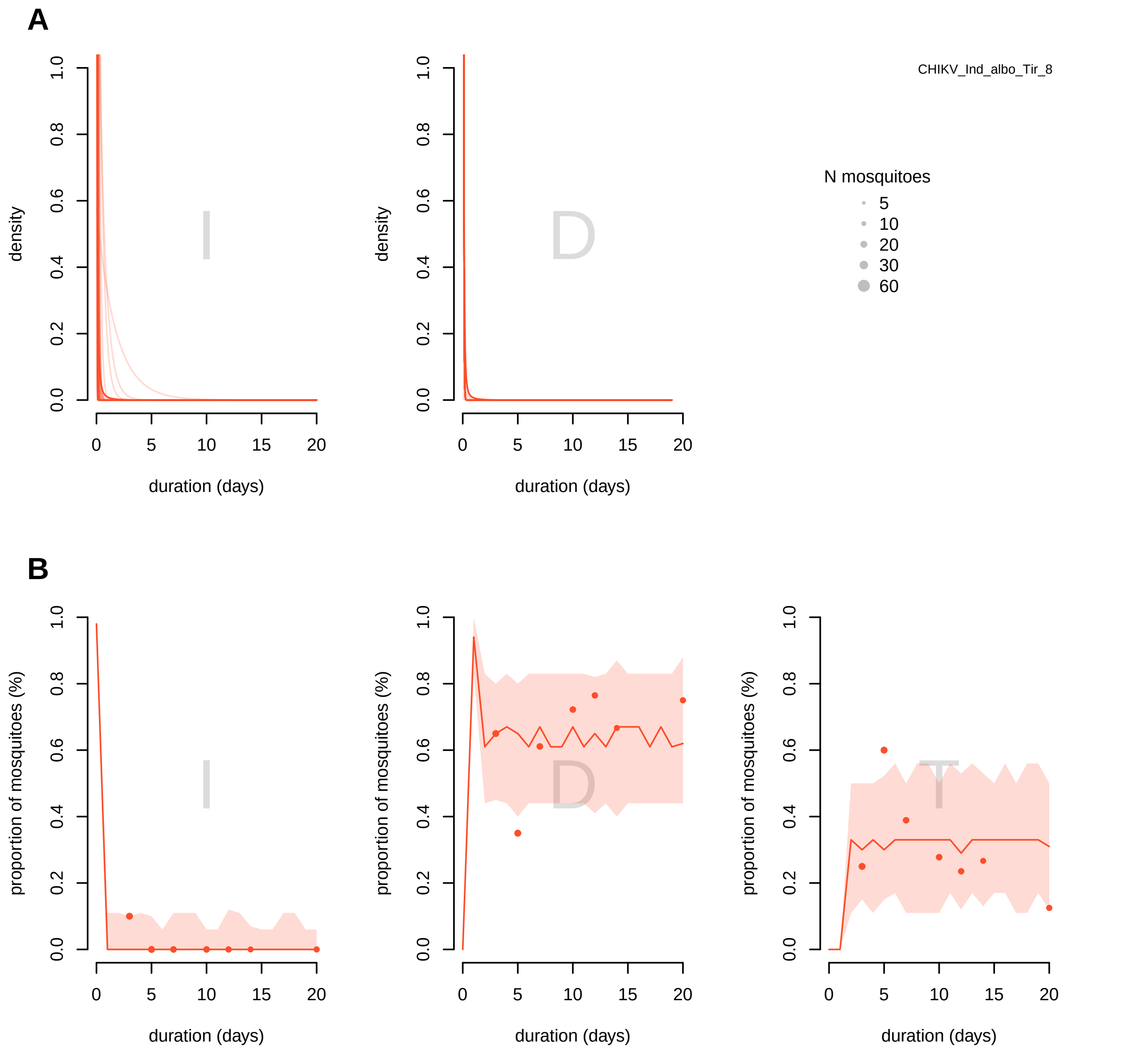

Fig I. Inference results for IVD stages distributions for scenario CHIKVc7(CHIKV_Ind_albo_Tir_8): *Aedes.albopictus* from Tirana infected by chikungunya virus from India with an infectious dose of 8 $\log_{10}$FFU/mL : A) Selected distributions in the infected and disseminated states for the main model selected. The dark line represents the mean of distributions and light lines represent a random sample of 50 distribution among all selected distribution. B) Selected dynamics in the infected (I), disseminated (D), and transmitter (T) states for the main model selected. The dots represent the observed data, the line (mean dynamics), and the uncertainty ribbons (5%-95%) represent selected simulated dynamic.

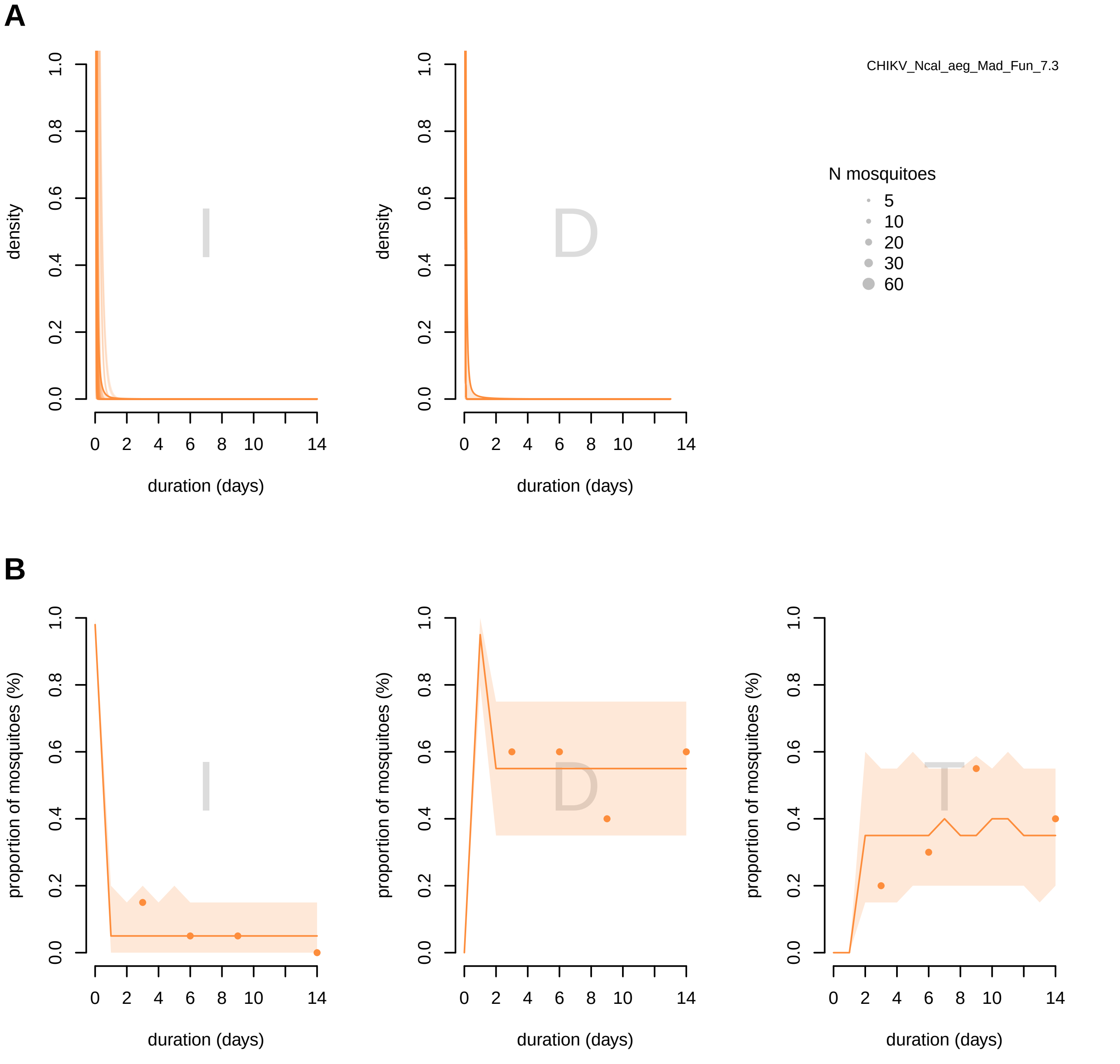

Fig J. Inference results for IVD stages distributions for scenario CHIKVc8(CHIKV_Ncal_aeg_Mad_Fun_7.3): *Aedes.aegypti* from Madeira Island, Funchal infected by chikungunya virus from New Caledonia with an infectious dose of 7.3 $\log_{10}$ FFU/mL : A) Selected distributions in the infected and disseminated states for the main model selected. The dark line represents the mean of distributions and light lines represent a random sample of 50 distribution among all selected distribution. B) Selected dynamics in the infected (I), disseminated (D), and transmitter (T) states for the main model selected. The dots represent the observed data, the line (mean dynamics), and the uncertainty ribbons (5%-95%) represent selected simulated dynamic.

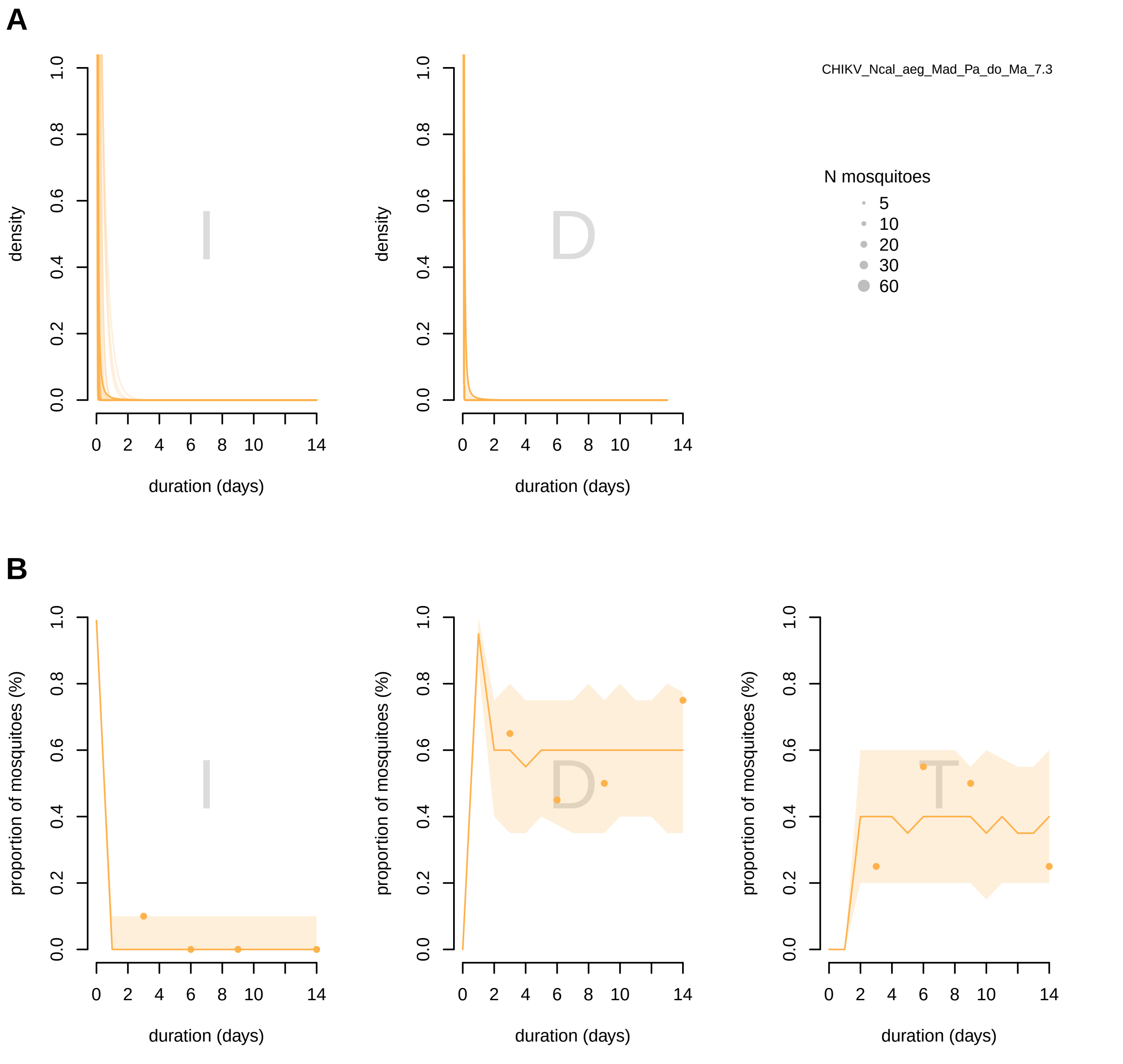

Fig K. Inference results for IVD stages distributions for scenario CHIKVc9(CHIKV_Ncal_aeg_Mad_Pa_do_Ma_7.3): *Aedes.aegypti* from Madeira Island, Paul do Mar infected by chikungunya virus from New Caledonia with an infectious dose of 7.3 $\log_{10}$ FFU/mL : A) Selected distributions in the infected and disseminated states for the main model selected. The dark line represents the mean of distributions and light lines represent a random sample of 50 distribution among all selected distribution. B) Selected dynamics in the infected (I), disseminated (D), and transmitter (T) states for the main model selected. The dots represent the observed data, the line (mean dynamics), and the uncertainty ribbons (5%-95%) represent selected simulated dynamic.

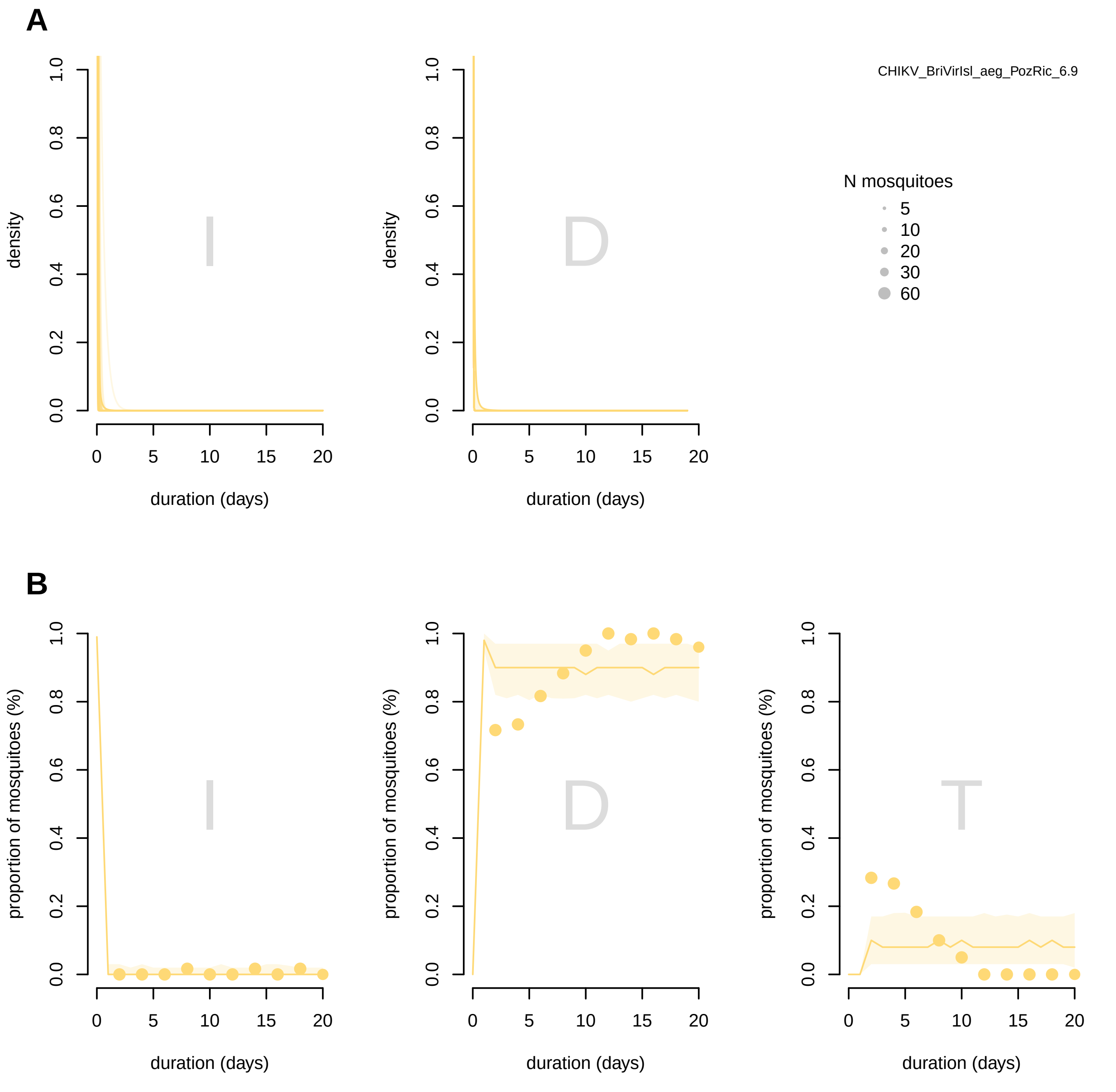

Fig L. Inference results for IVD stages distributions for scenario CHIKVc10(CHIKV_BriVirIsl_aeg_PozRic_6.9): *Aedes.aegypti* from Mexico, Poza Rica, infected by chikungunya virus from British Virgin Island with an infectious dose of 6.9$\log_{10}$ PFU/mL : A) Selected distributions in the infected and disseminated states for the main model selected. The dark line represents the mean of distributions and light lines represent a random sample of 50 distribution among all selected distribution. B) Selected dynamics in the infected (I), disseminated (D), and transmitter (T) states for the main model selected. The dots represent the observed data, the line (mean dynamics), and the uncertainty ribbons (5%-95%) represent selected simulated dynamic.

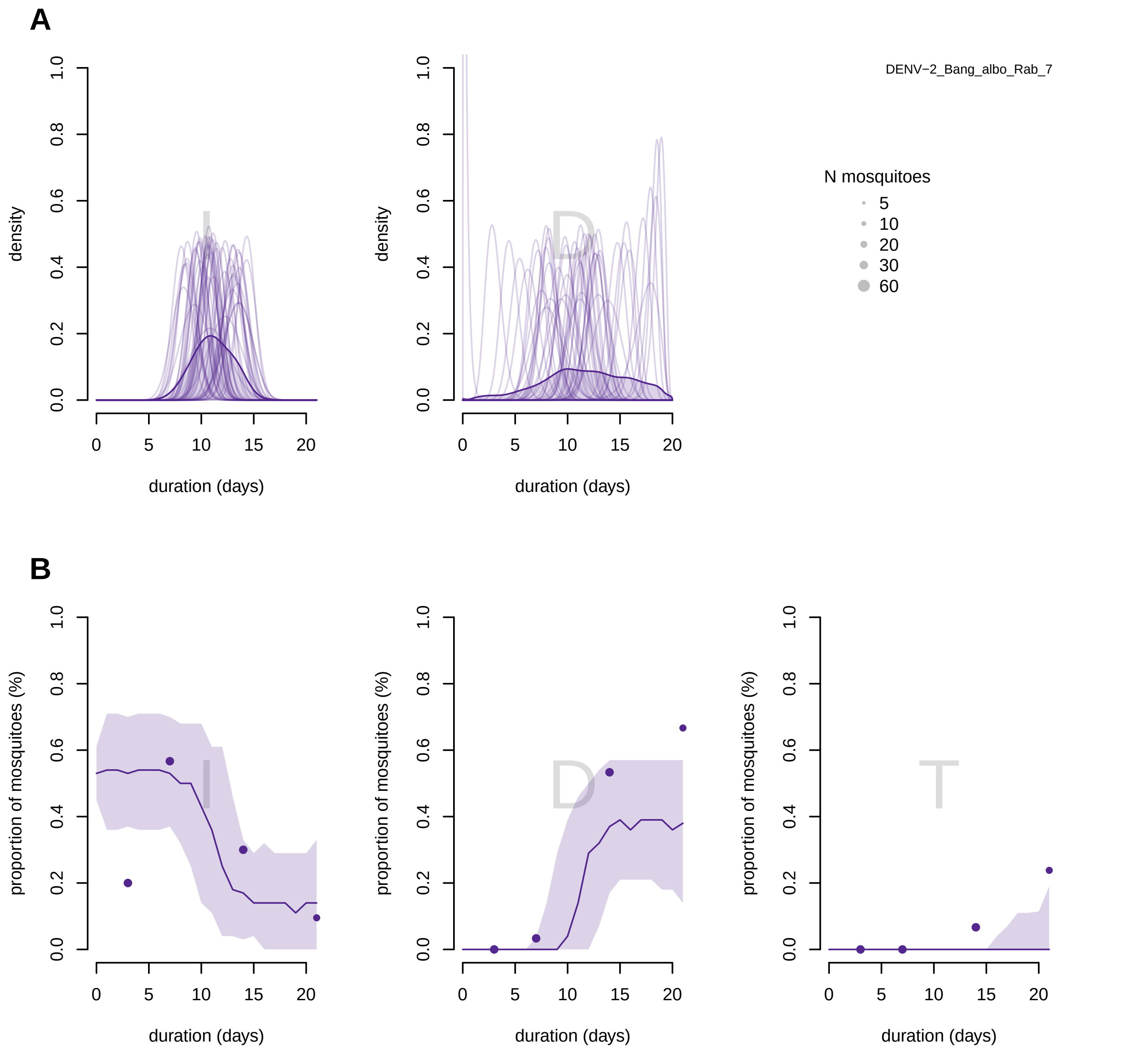

Fig M. Inference results for IVD stages distributions for scenario DENVc1(DENV2_Bang_albo_Rab_7): *Aedes.albopictus* from Rabat infected by dengue virus from Bangkok with an infectious dose of 7 $\log_{10}$ FFU/mL : A) Selected distributions in the infected and disseminated states for the main model selected. The dark line represents the mean of distributions and light lines represent a random sample of 50 distribution among all selected distribution. B) Selected dynamics in the infected (I), disseminated (D), and transmitter (T) states for the main model selected. The dots represent the observed data, the line (mean dynamics), and the uncertainty ribbons (5%-95%) represent selected simulated dynamic.

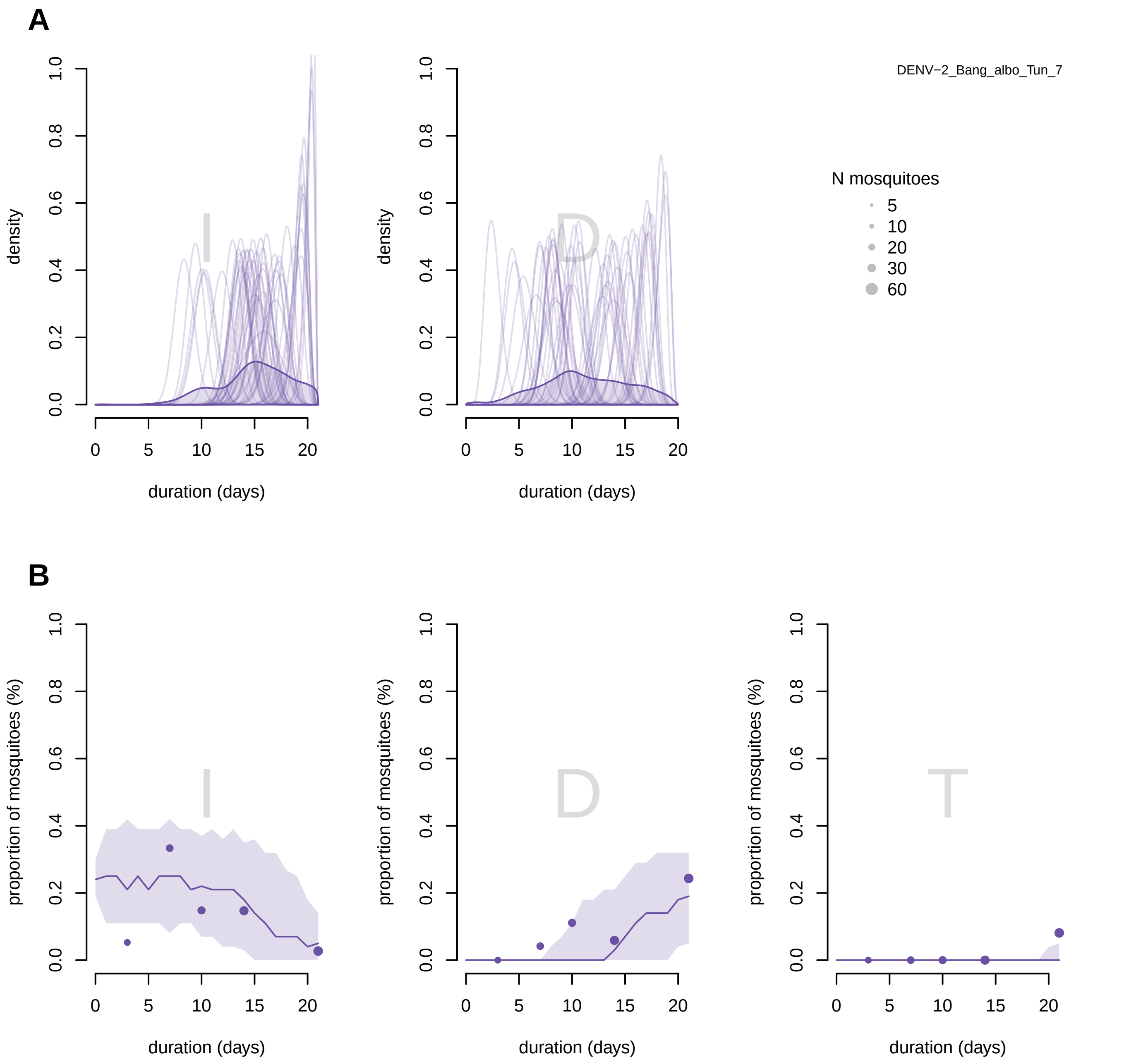

Fig N. Inference results for IVD stages distributions for scenario DENVc2(DENV2_Bang_albo_Tun_7): *Aedes.albopictus* from Tunisia infected by dengue virus from Bangkok with an infectious dose of 7 $\log_{10}$ TCID50/mL : A) Selected distributions in the infected and disseminated states for the main model selected. The dark line represents the mean of distributions and light lines represent a random sample of 50 distribution among all selected distribution. B) Selected dynamics in the infected (I), disseminated (D), and transmitter (T) states for the main model selected. The dots represent the observed data, the line (mean dynamics), and the uncertainty ribbons (5%-95%) represent selected simulated dynamic.

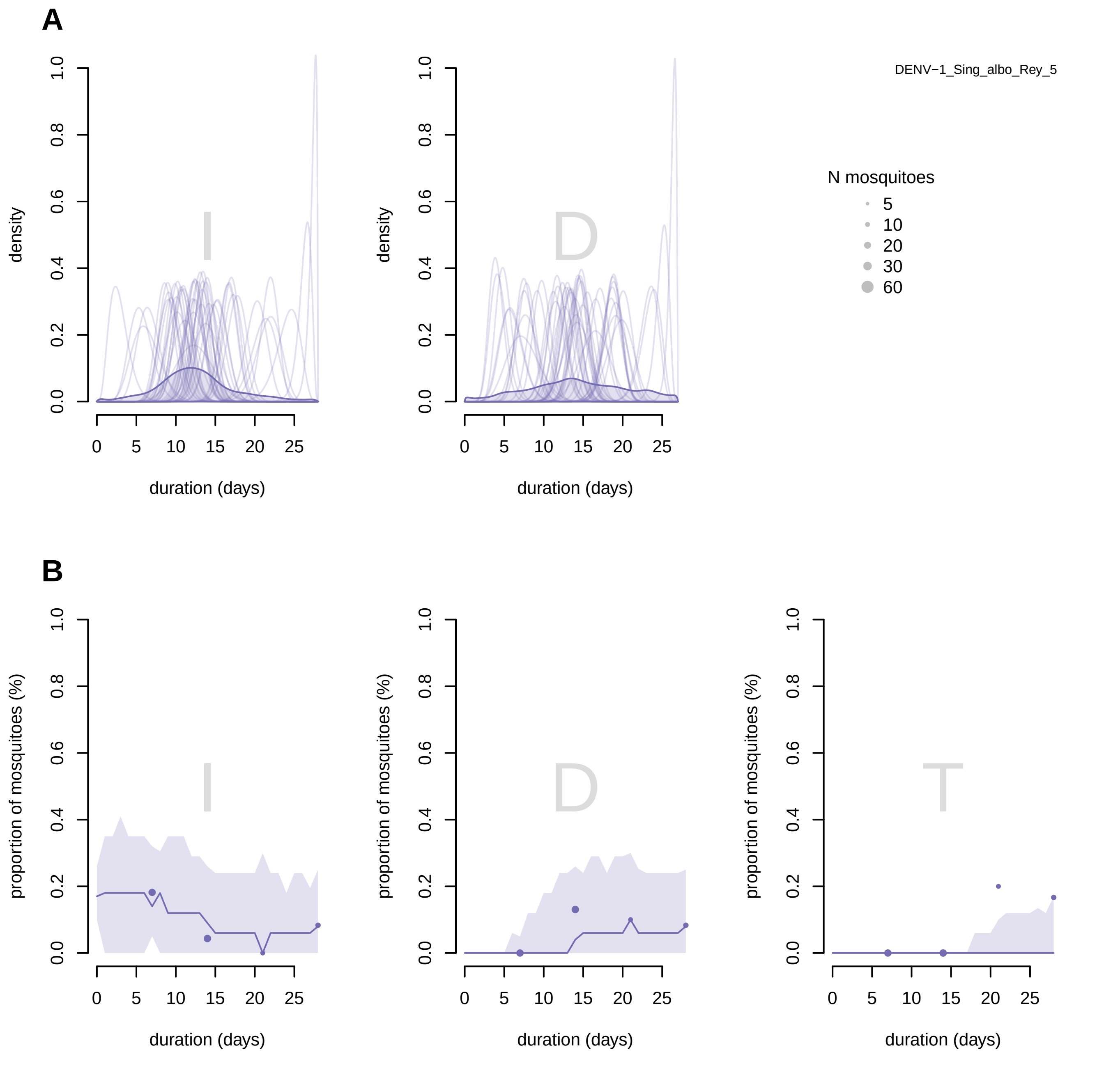

Fig O. Inference results for IVD stages distributions for scenario DENVc3(DENV1_Sing_albo_Rey_5): *Aedes.albopictus* from Reynosa infected by dengue virus from Singapore with an infectious dose of 5 $\log_{10}$ PFU/mL : A) Selected distributions in the infected and disseminated states for the main model selected. The dark line represents the mean of distributions and light lines represent a random sample of 50 distribution among all selected distribution. B) Selected dynamics in the infected (I), disseminated (D), and transmitter (T) states for the main model selected. The dots represent the observed data, the line (mean dynamics), and the uncertainty ribbons (5%-95%) represent selected simulated dynamic.

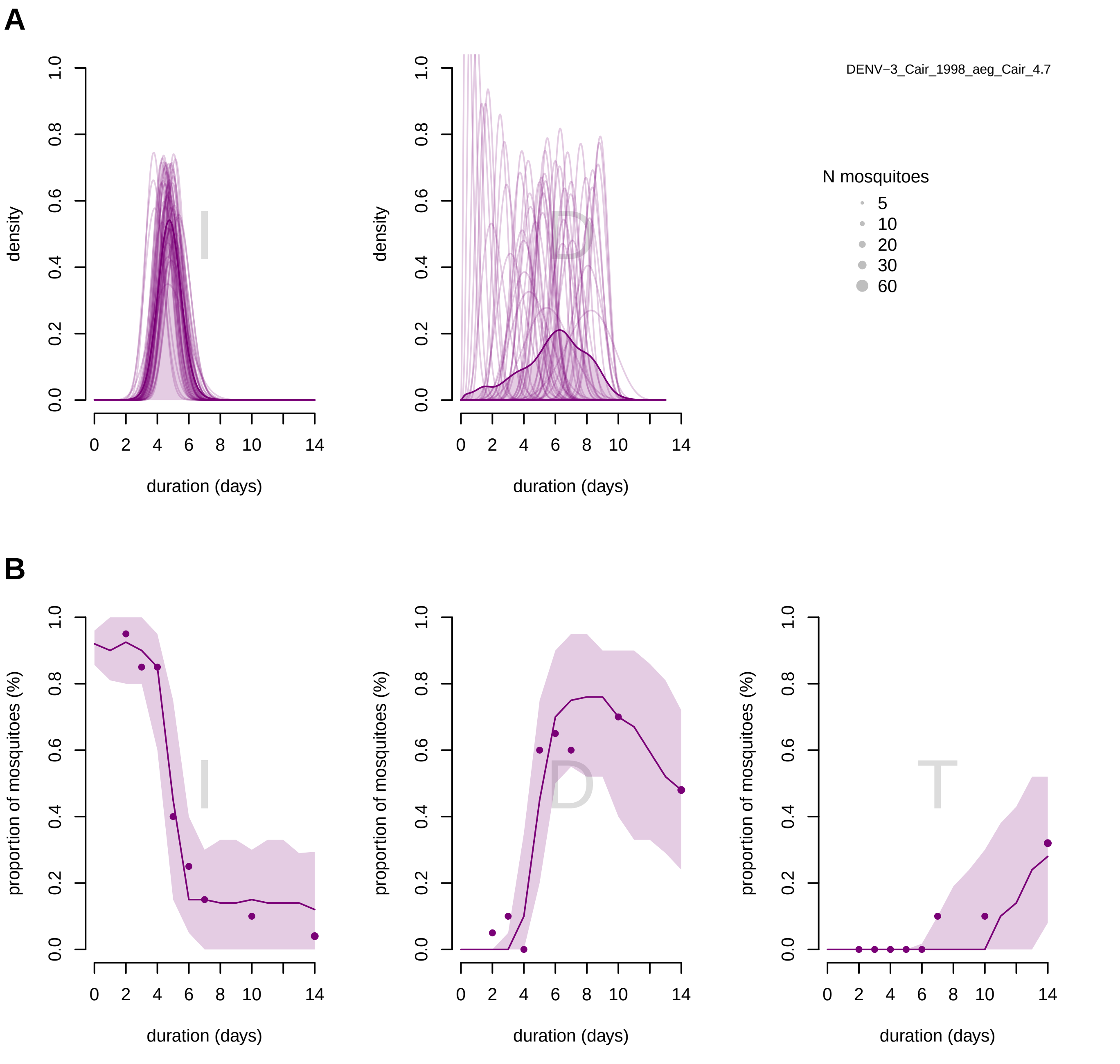

Fig P. Inference results for IVD stages distributions for scenario DENVc4(DENV3_Cair_1998_aeg_Cair_4.7): *Aedes.aegypti* from Cairns infected by dengue virus from Cairns with an infectious dose of 4.9 $\log_{10}$ CCID50/mosquito : A) Selected distributions in the infected and disseminated states for the main model selected. The dark line represents the mean of distributions and light lines represent a random sample of 50 distribution among all selected distribution. B) Selected dynamics in the infected (I), disseminated (D), and transmitter (T) states for the main model selected. The dots represent the observed data, the line (mean dynamics), and the uncertainty ribbons (5%-95%) represent selected simulated dynamic.

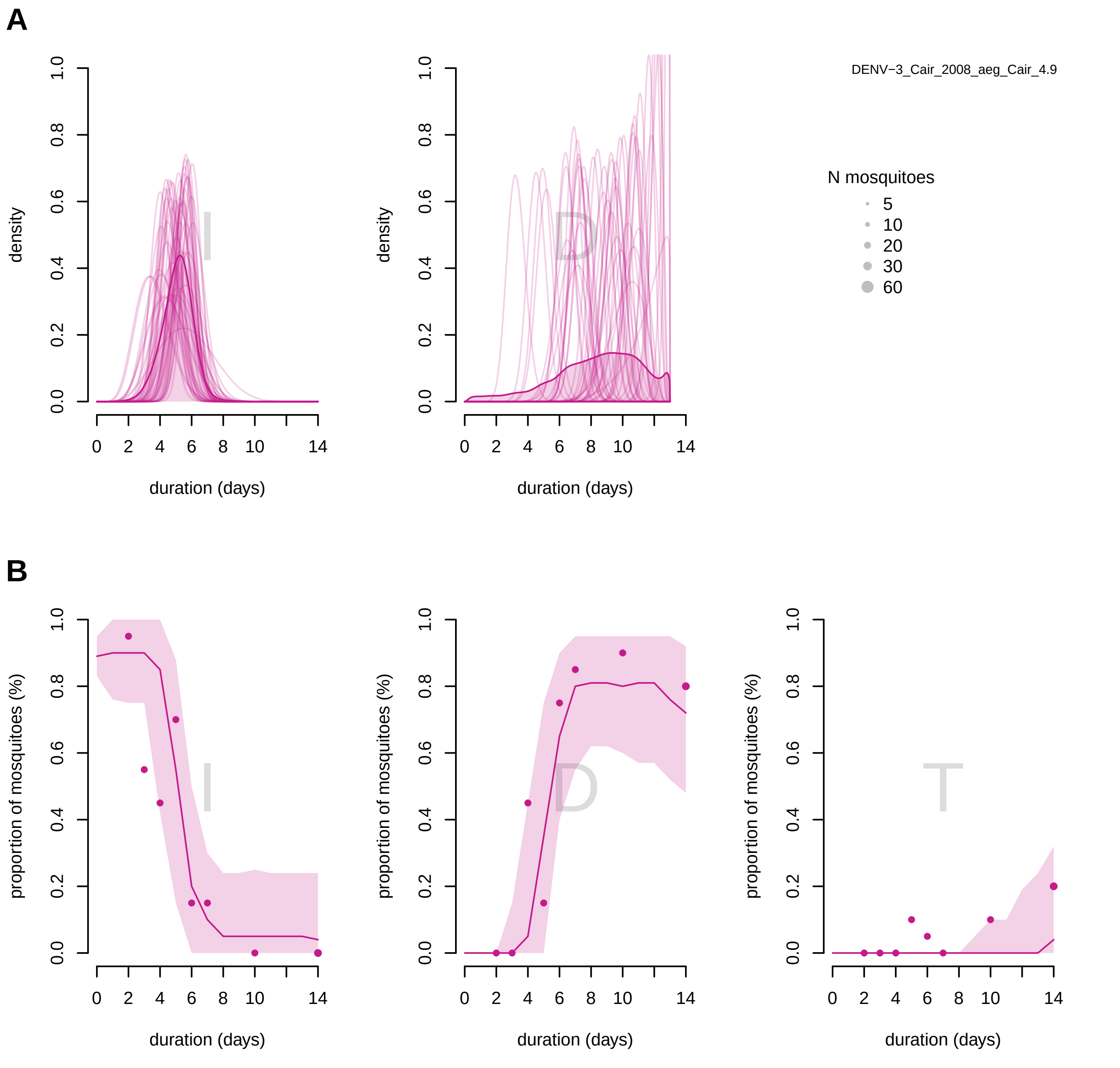

Fig Q. Inference results for IVD stages distributions for scenario DENVc5(DENV3_Cair_2008_aeg_Cair_4.9): *Aedes.aegypti* from Cairns infected by dengue virus from Cairns with an infectious dose of 5.1 $\log_{10}$ CCID50/mosquito: A) Selected distributions in the infected and disseminated states for the main model selected. The dark line represents the mean of distributions and light lines represent a random sample of 50 distribution among all selected distribution. B) Selected dynamics in the infected (I), disseminated (D), and transmitter (T) states for the main model selected. The dots represent the observed data, the line (mean dynamics), and the uncertainty ribbons (5%-95%) represent selected simulated dynamic.

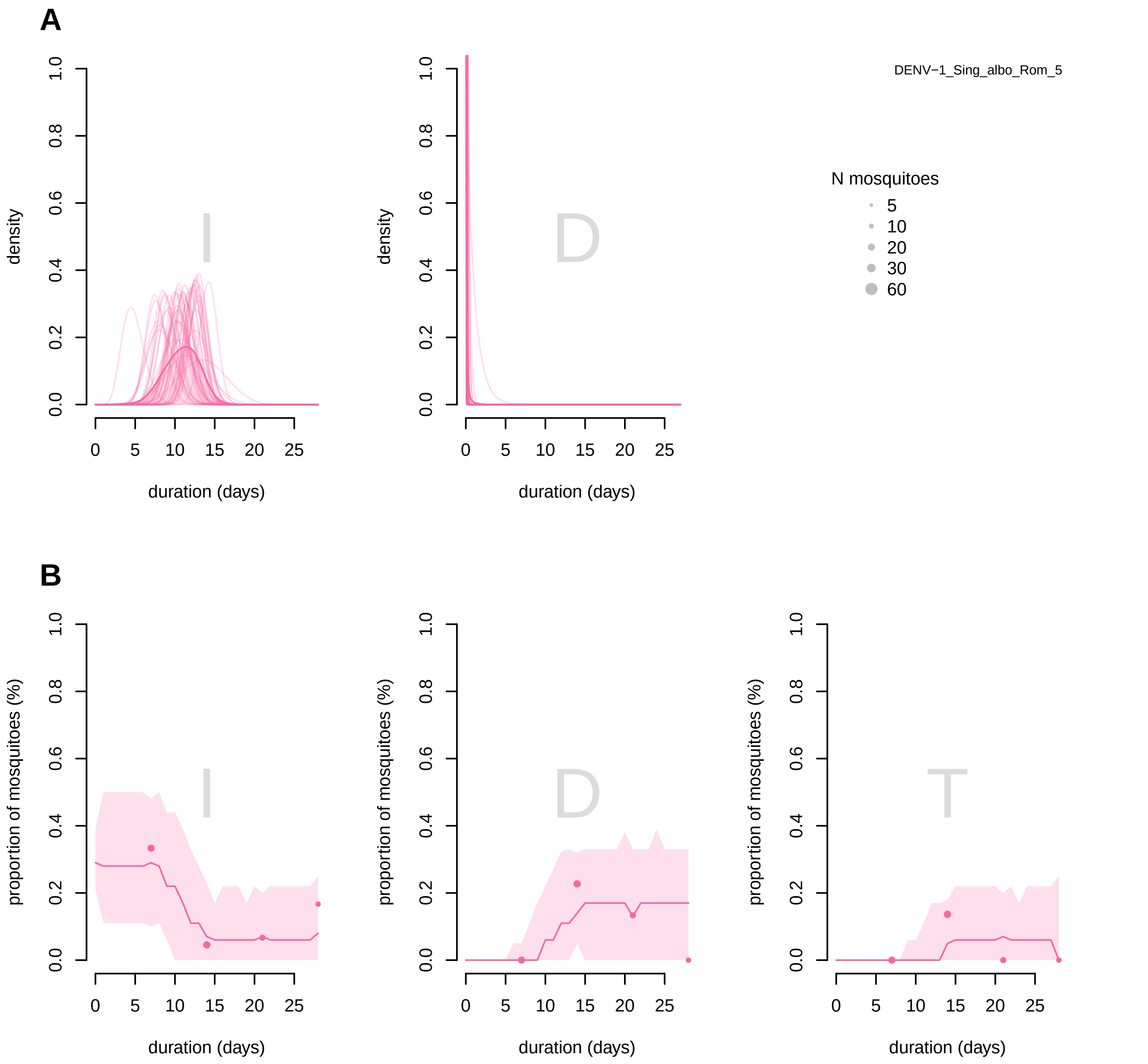

Fig R. Inference results for IVD stages distributions for scenario DENVc6(DENV1_Sing_albo_Rom_5): *Aedes.albopictus* from Roma infected by dengue virus from Singapore with an infectious dose of 5 $\log_{10}$ PFU/mL : A) Selected distributions in the infected and disseminated states for the main model selected. The dark line represents the mean of distributions and light lines represent a random sample of 50 distribution among all selected distribution. B) Selected dynamics in the infected (I), disseminated (D), and transmitter (T) states for the main model selected. The dots represent the observed data, the line (mean dynamics), and the uncertainty ribbons (5%-95%) represent selected simulated dynamic.

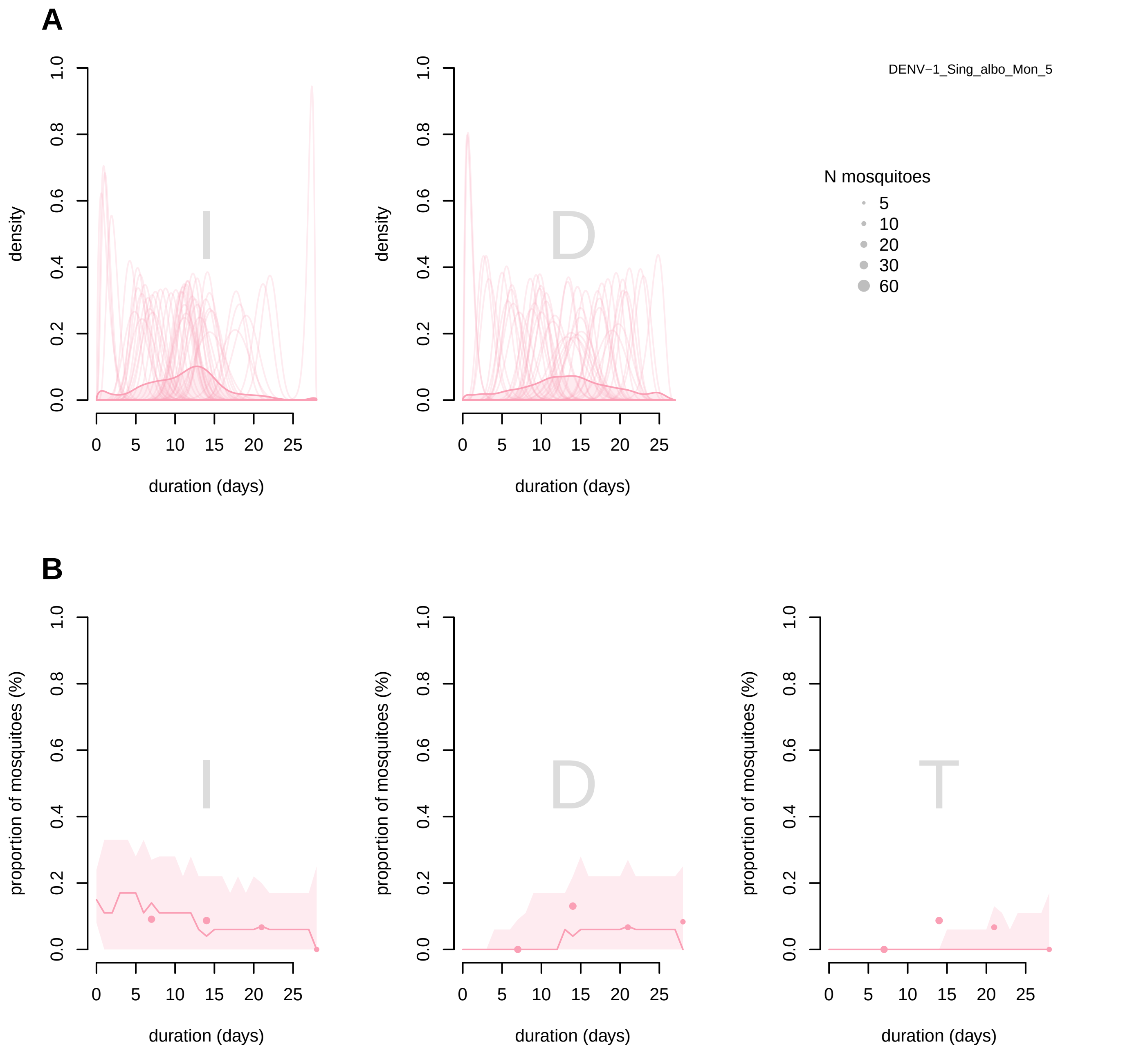

Fig S. Inference results for IVD stages distributions for scenario DENVc7(DENV1_Sing_albo_Mon_5): *Aedes.albopictus* from Montecchio infected by dengue virus from Singapore with an infectious dose of 5 $\log_{10}$ PFU/mL : A) Selected distributions in the infected and disseminated states for the main model selected. The dark line represents the mean of distributions and light lines represent a random sample of 50 distribution among all selected distribution. B) Selected dynamics in the infected (I), disseminated (D), and transmitter (T) states for the main model selected. The dots represent the observed data, the line (mean dynamics), and the uncertainty ribbons (5%-95%) represent selected simulated dynamic.

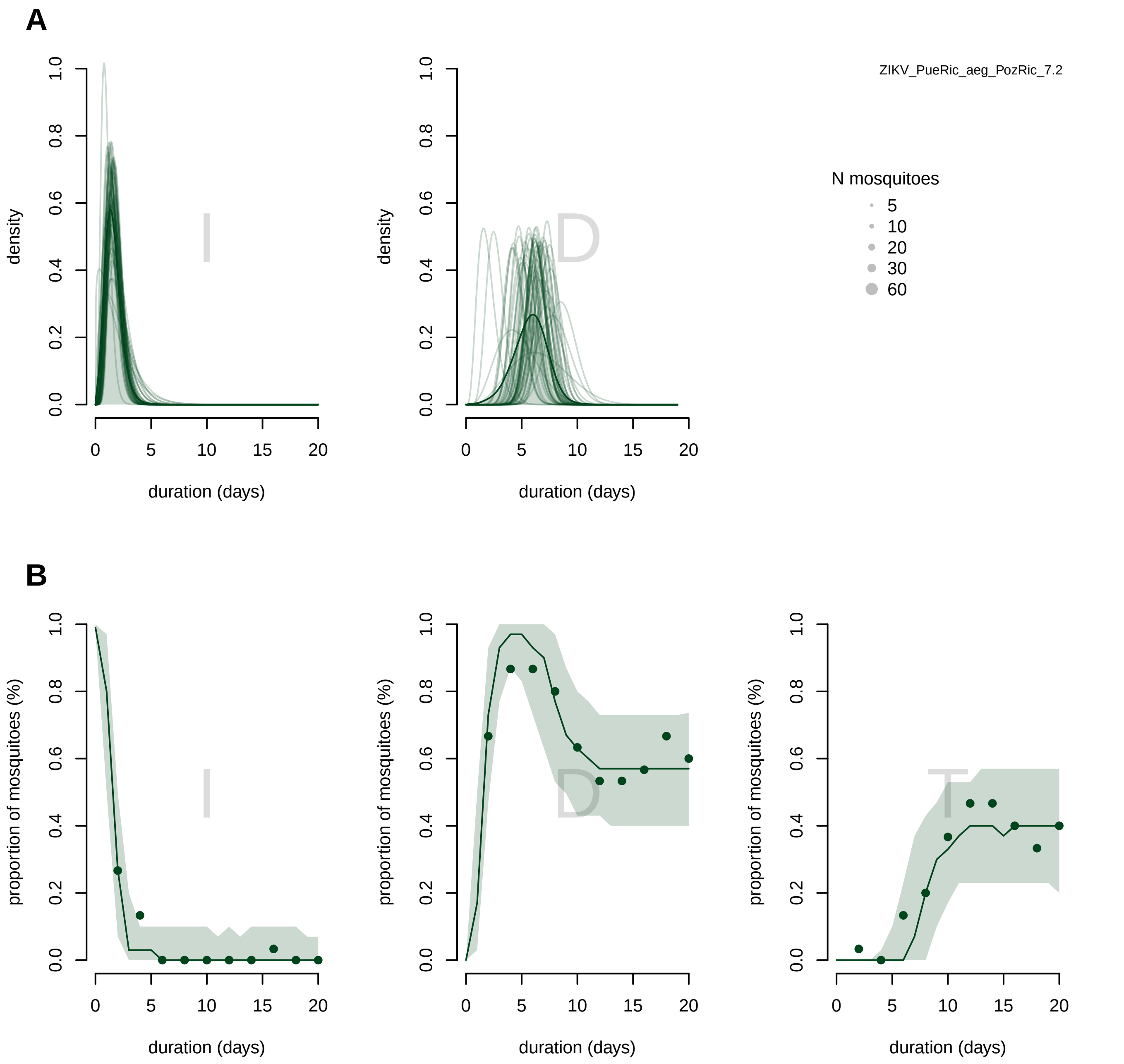

Fig T. Inference results for IVD stages distributions for scenario ZIKVc1(ZIKV_PueRic_aeg_PozRic_7.2): *Aedes.aegypti* from Poza Rica infected by Zika virus from Puerto Rico with an infectious dose of 7.2 $\log_{10}$ PFU/mL : A) Selected distributions in the infected and disseminated states for the main model selected. The dark line represents the mean of distributions and light lines represent a random sample of 50 distribution among all selected distribution. B) Selected dynamics in the infected (I), disseminated (D), and transmitter (T) states for the main model selected. The dots represent the observed data, the line (mean dynamics), and the uncertainty ribbons (5%-95%) represent selected simulated dynamic.

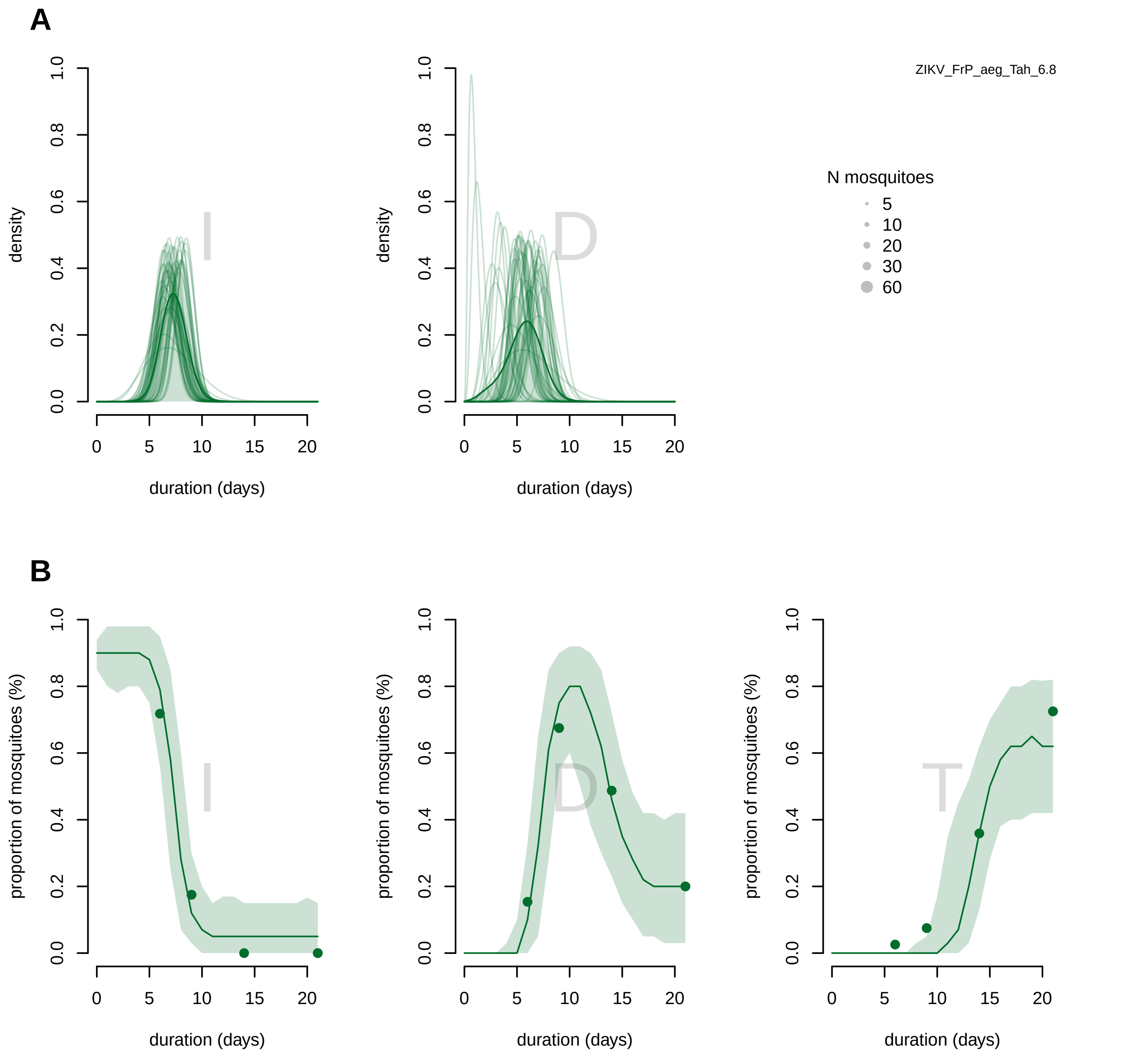

Fig U. Inference results for IVD stages distributions for scenario ZIKVc2(ZIKV_FrP_aeg_Tah_6.8): *Aedes.aegypti* from Tahiti infected by Zika virus from French Polynesia with an infectious dose of 6.8 $\log_{10}$ TCID50/mL : A) Selected distributions in the infected and disseminated states for the main model selected. The dark line represents the mean of distributions and light lines represent a random sample of 50 distribution among all selected distribution. B) Selected dynamics in the infected (I), disseminated (D), and transmitter (T) states for the main model selected. The dots represent the observed data, the line (mean dynamics), and the uncertainty ribbons (5%-95%) represent selected simulated dynamic.

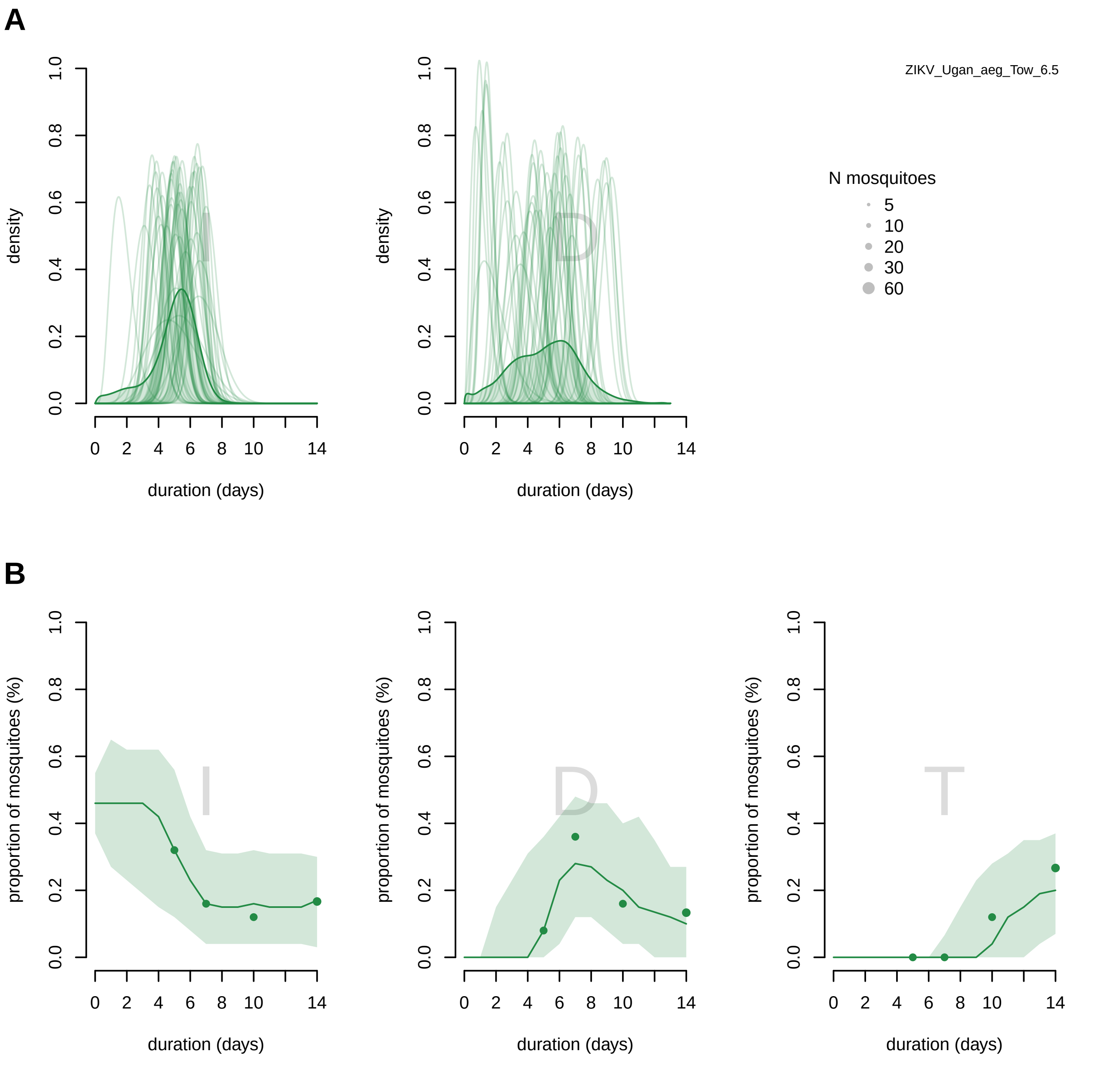

Fig V. Inference results for IVD stages distributions for scenario ZIKVc3(ZIKV_Ugan_aeg_Tow_6.5): *Aedes.aegypti* from Townsville infected by Zika virus from Uganda with an infectious dose of 6.5 $\log_{10}$ TCID50/mL: A) Selected distributions in the infected and disseminated states for the main model selected. The dark line represents the mean of distributions and light lines represent a random sample of 50 distribution among all selected distribution. B) Selected dynamics in the infected (I), disseminated (D), and transmitter (T) states for the main model selected. The dots represent the observed data, the line (mean dynamics), and the uncertainty ribbons (5%-95%) represent selected simulated dynamic.

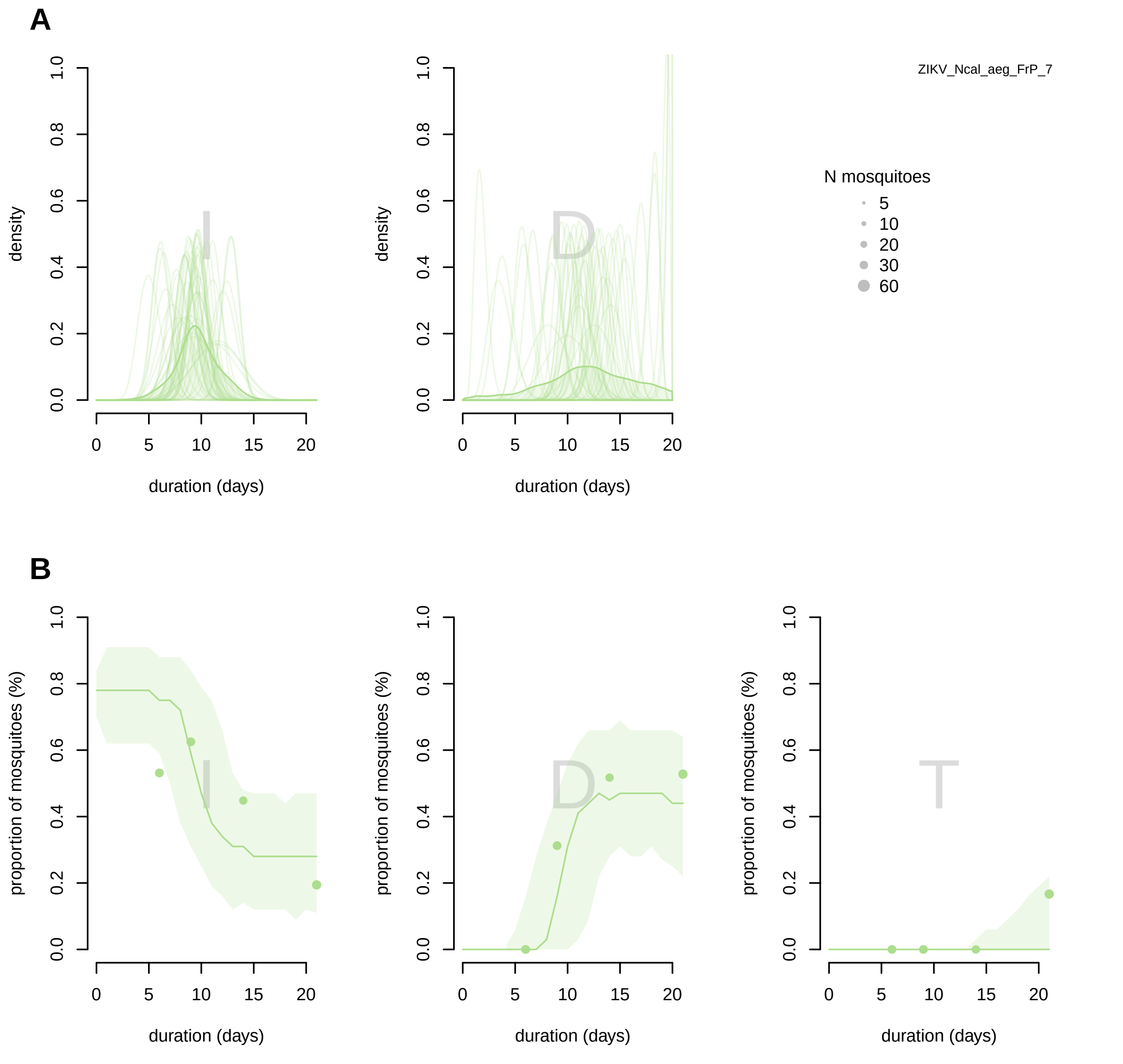

Fig W. Inference results for IVD stages distributions for scenario ZIKVc4(ZIKV_Ncal_aeg_FrP_7): *Aedes.aegypti* from French Polynesia infected by Zika virus from New Caledonia with an infectious dose of 7 $\log_{10}$ TCID50/mL : A) Selected distributions in the infected and disseminated states for the main model selected. The dark line represents the mean of distributions and light lines represent a random sample of 50 distribution among all selected distribution. B) Selected dynamics in the infected (I), disseminated (D), and transmitter (T) states for the main model selected. The dots represent the observed data, the line (mean dynamics), and the uncertainty ribbons (5%-95%) represent selected simulated dynamic.

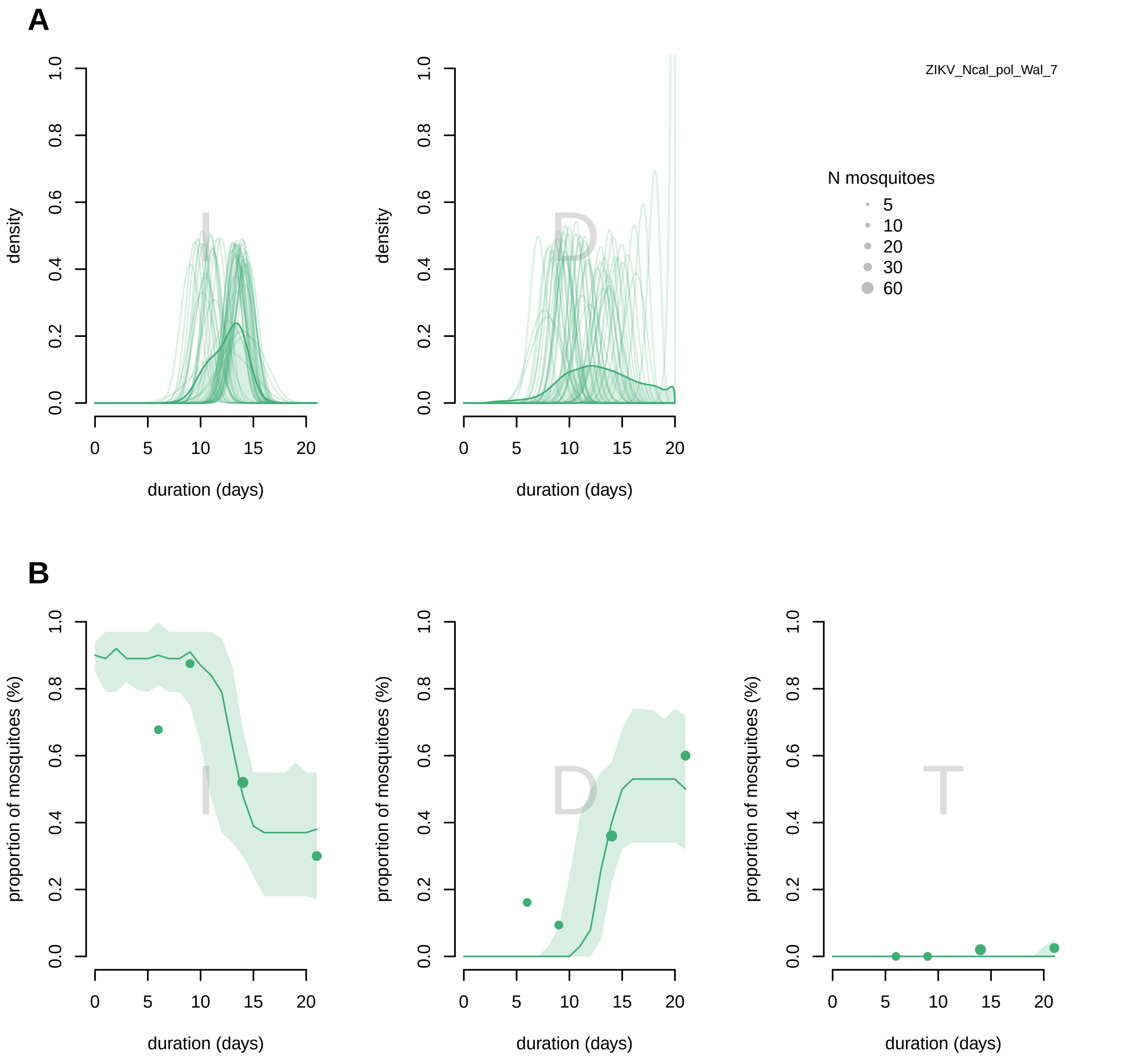

Fig X. Inference results for IVD stages distributions for scenario ZIKVc5(ZIKV_Ncal_pol_Wal_7): *Aedes.polynesiensis* from Wallis infected by Zika virus from New Caledonia with an infectious dose of 7 $\log_{10}$ TCID50/mL : A) Selected distributions in the infected and disseminated states for the main model selected. The dark line represents the mean of distributions and light lines represent a random sample of 50 distribution among all selected distribution. B) Selected dynamics in the infected (I), disseminated (D), and transmitter (T) states for the main model selected. The dots represent the observed data, the line (mean dynamics), and the uncertainty ribbons (5%-95%) represent selected simulated dynamic.

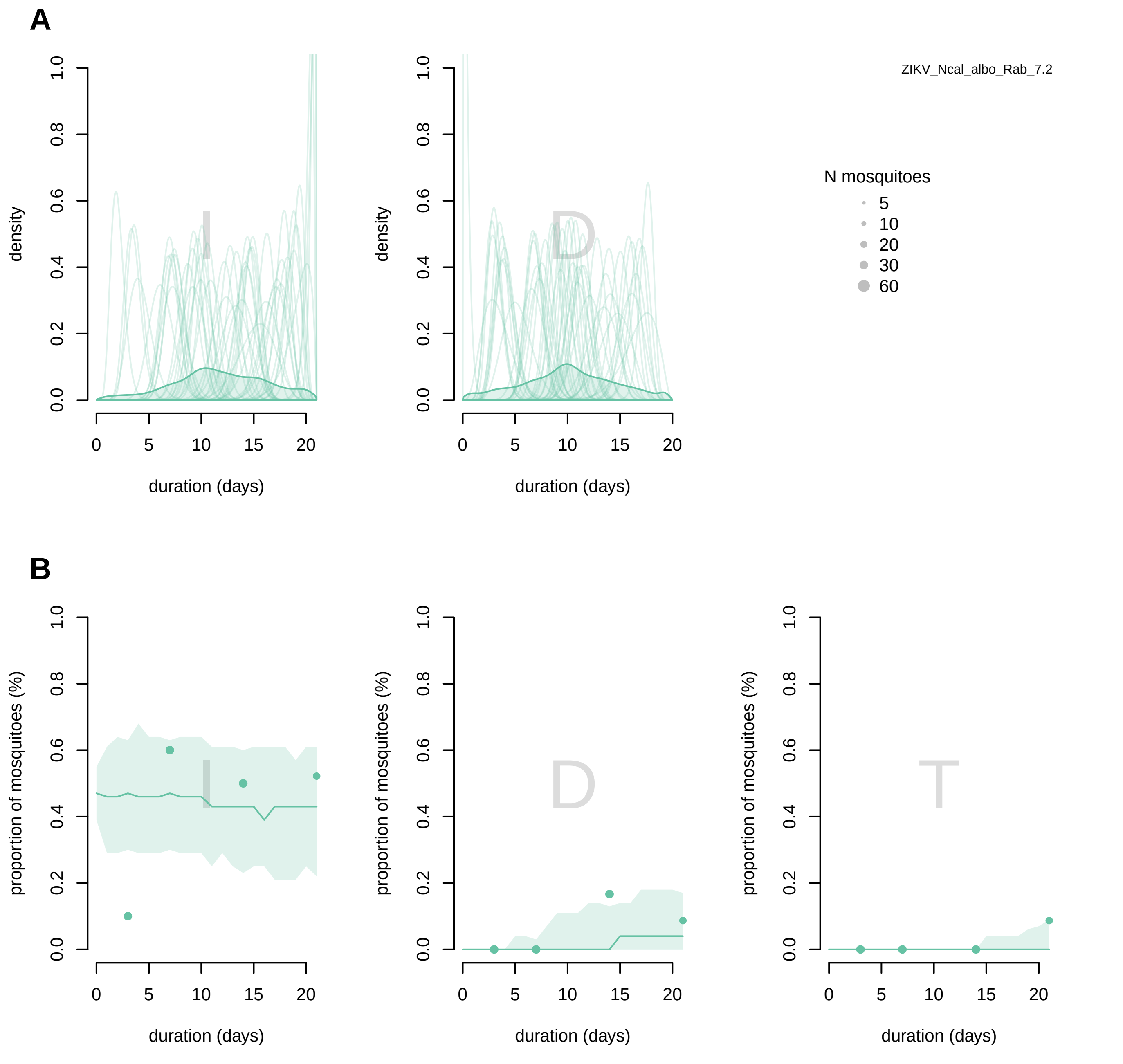

Fig Y. Inference results for IVD stages distributions for scenario ZIKVc6(ZIKV_Ncal_albo_Rab_7.2): *Aedes.albopictus* from Rabat infected by Zika virus from New Caledonia with an infectious dose of 7.2 $\log_{10}$ PFU/mL : A) Selected distributions in the infected and disseminated states for the main model selected. The dark line represents the mean of distributions and light lines represent a random sample of 50 distribution among all selected distribution. B) Selected dynamics in the infected (I), disseminated (D), and transmitter (T) states for the main model selected. The dots represent the observed data, the line (mean dynamics), and the uncertainty ribbons (5%-95%) represent selected simulated dynamic.

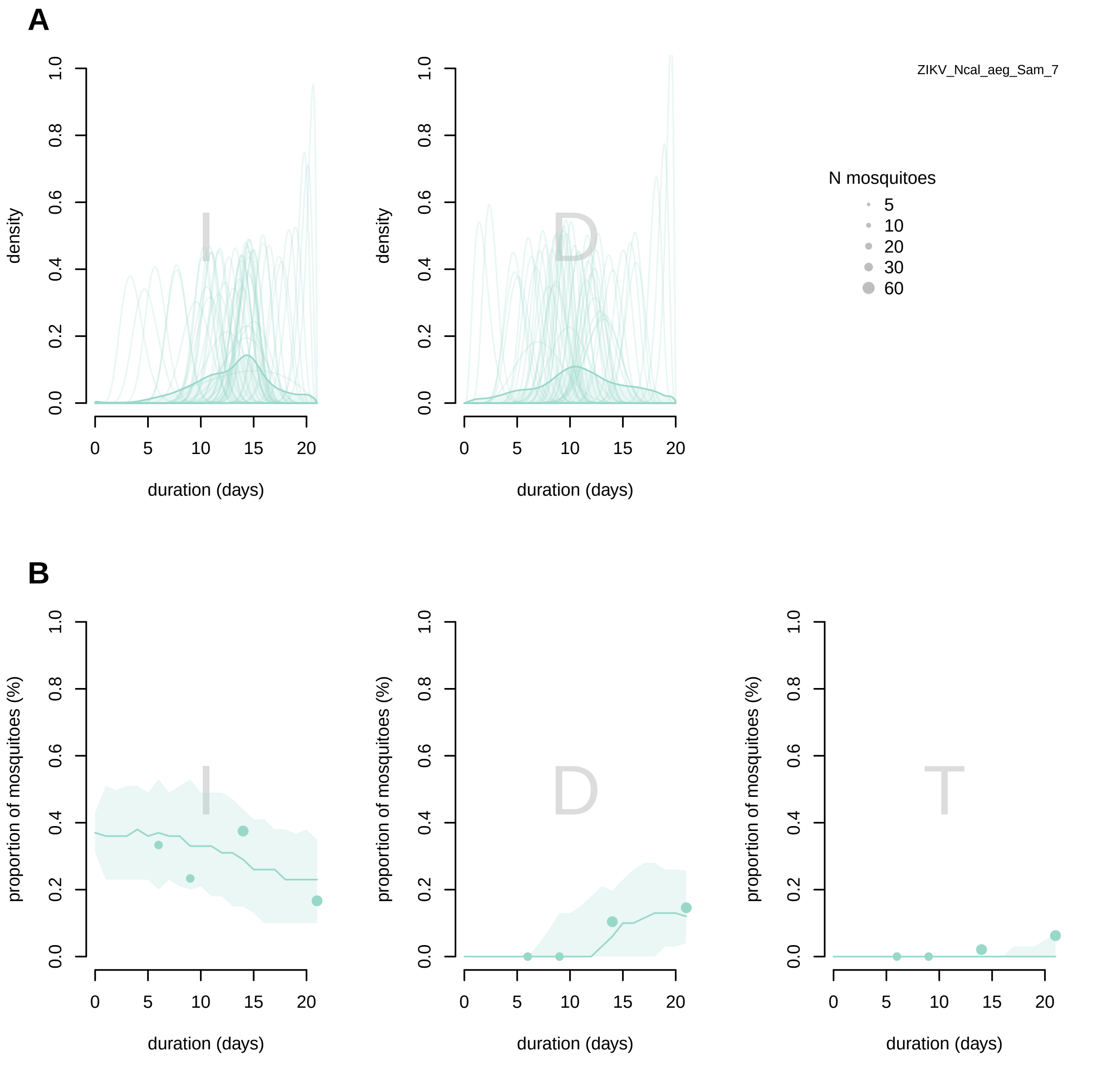

Fig Z. Inference results for IVD stages distributions for scenario ZIKVc7(ZIKV_Ncal_aeg_Sam_7): *Aedes.aegypti* from Samoa infected by Zika virus from New Caledonia with an infectious dose of 7 $\log_{10}$ TCID50/mL : A) Selected distributions in the infected and disseminated states for the main model selected. The dark line represents the mean of distributions and light lines represent a random sample of 50 distribution among all selected distribution. B) Selected dynamics in the infected (I), disseminated (D), and transmitter (T) states for the main model selected. The dots represent the observed data, the line (mean dynamics), and the uncertainty ribbons (5%-95%) represent selected simulated dynamic.

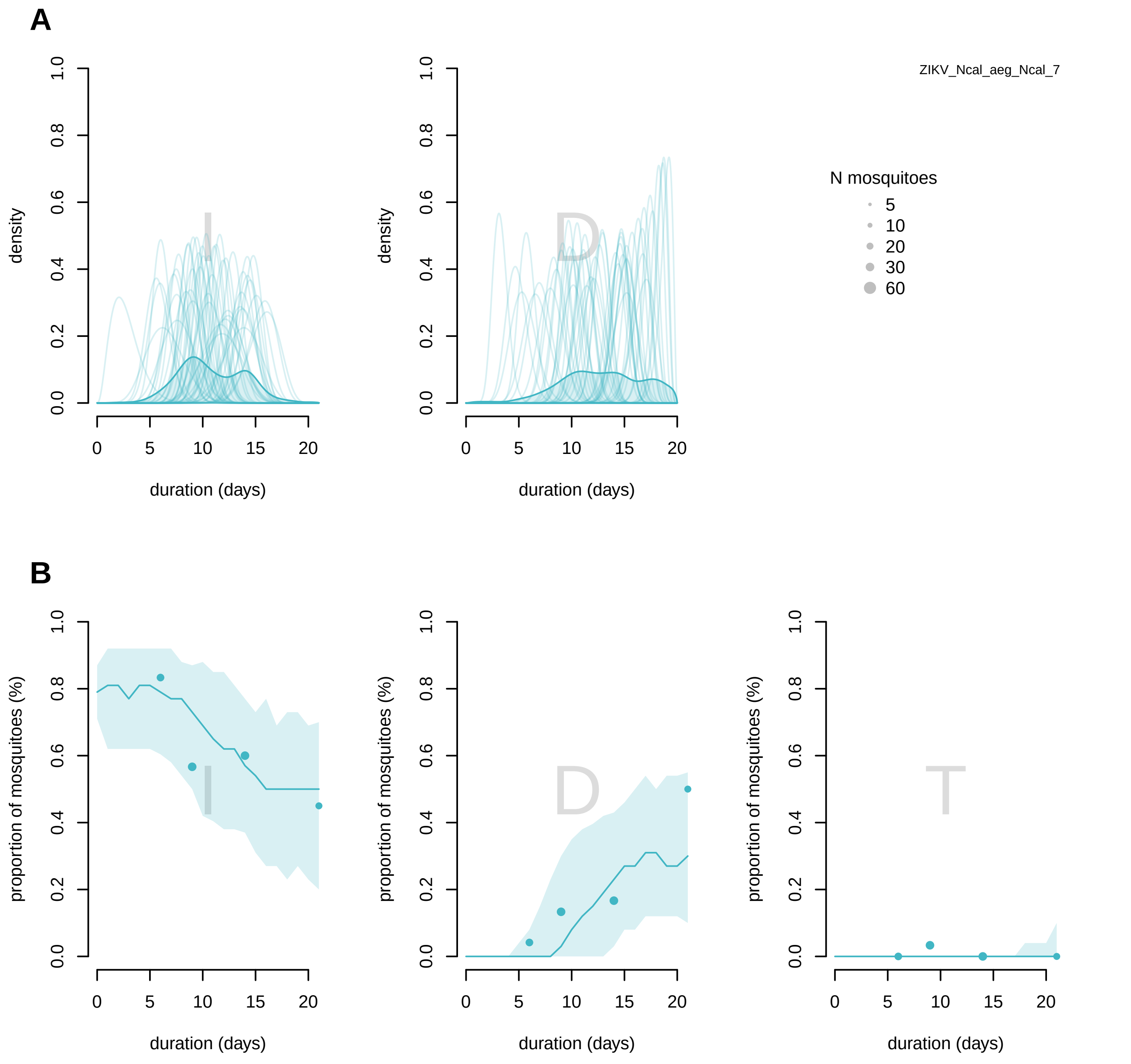

Fig AA. Inference results for IVD stages distributions for scenario ZIKVc8(ZIKV_Ncal_aeg_Ncal_7): *Aedes.aegypti* from New caledonia infected by Zika virus (NC-2014-5132) from New Caledonia with an infectious dose of 7 $\log_{10}$ TCID50/mL : A) Selected distributions in the infected and disseminated states for the main model selected. The dark line represents the mean of distributions and light lines represent a random sample of 50 distribution among all selected distribution. B) Selected dynamics in the infected (I), disseminated (D), and transmitter (T) states for the main model selected. The dots represent the observed data, the line (mean dynamics), and the uncertainty ribbons (5%-95%) represent selected simulated dynamic.

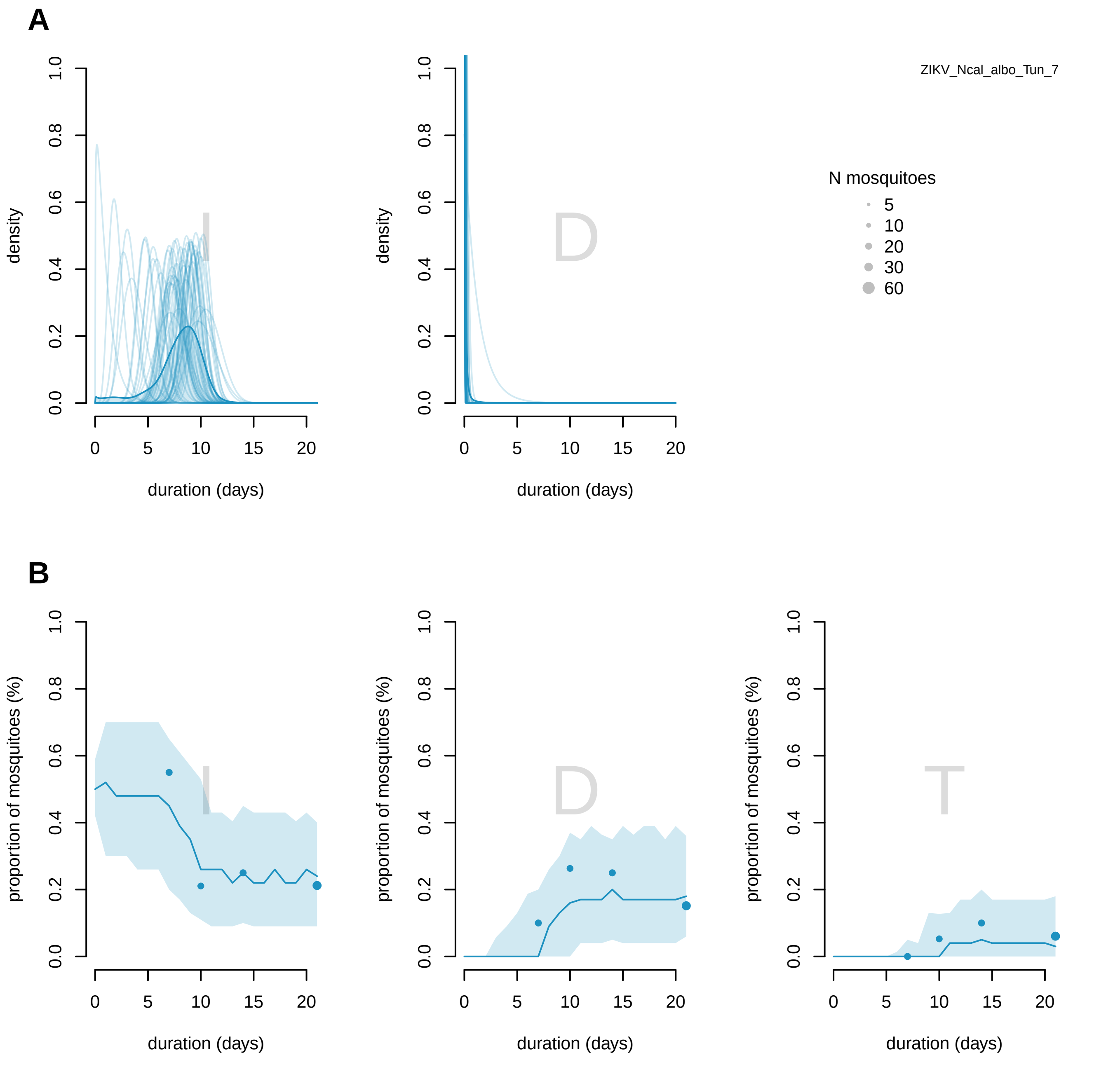

Fig AB. Inference results for IVD stages distributions for scenario ZIKVc9(ZIKV_Ncal_albo_Tun_7): *Aedes.albopictus* from Tunisia infected by Zika virus from New Caledonia with an infectious dose of 7 $\log_{10}$ TCID50/mL : A) Selected distributions in the infected and disseminated states for the main model selected. The dark line represents the mean of distributions and light lines represent a random sample of 50 distribution among all selected distribution. B) Selected dynamics in the infected (I), disseminated (D), and transmitter (T) states for the main model selected. The dots represent the observed data, the line (mean dynamics), and the uncertainty ribbons (5%-95%) represent selected simulated dynamic.

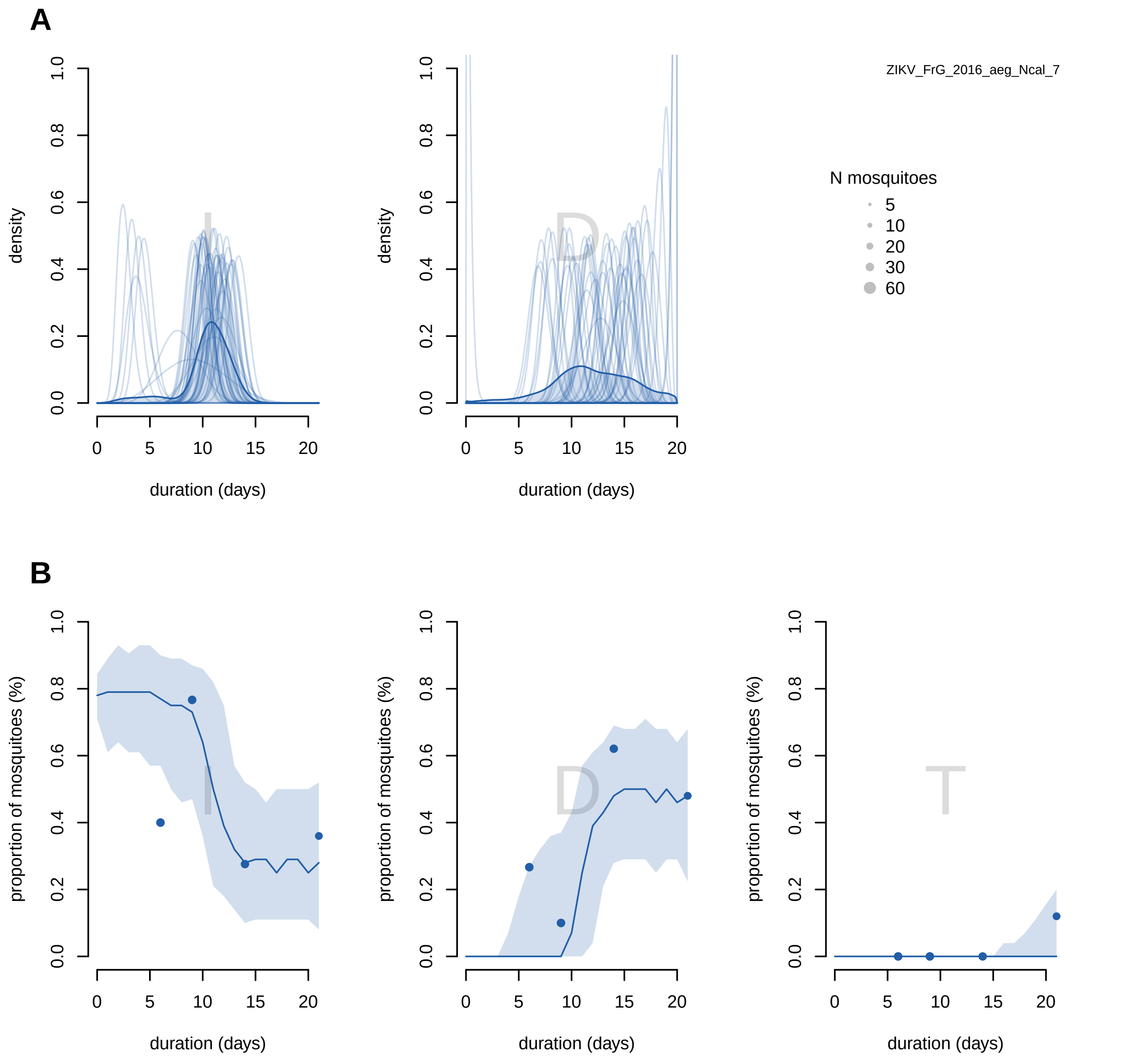

Fig AC. Inference results for IVD stages distributions for scenario ZIKVc10(ZIKV_FrG_2016_aeg_Ncal_7): *Aedes.aegypti* from New caledonia infected by Zika virus from French Guiana (2016) with an infectious dose of 7 log10 FFU/mL : A) Selected distributions in the infected and disseminated states for the main model selected. The dark line represents the mean of distributions and light lines represent a random sample of 50 distribution among all selected distribution. B) Selected dynamics in the infected (I), disseminated (D), and transmitter (T) states for the main model selected. The dots represent the observed data, the line (mean dynamics), and the uncertainty ribbons (5%-95%) represent selected simulated dynamic.

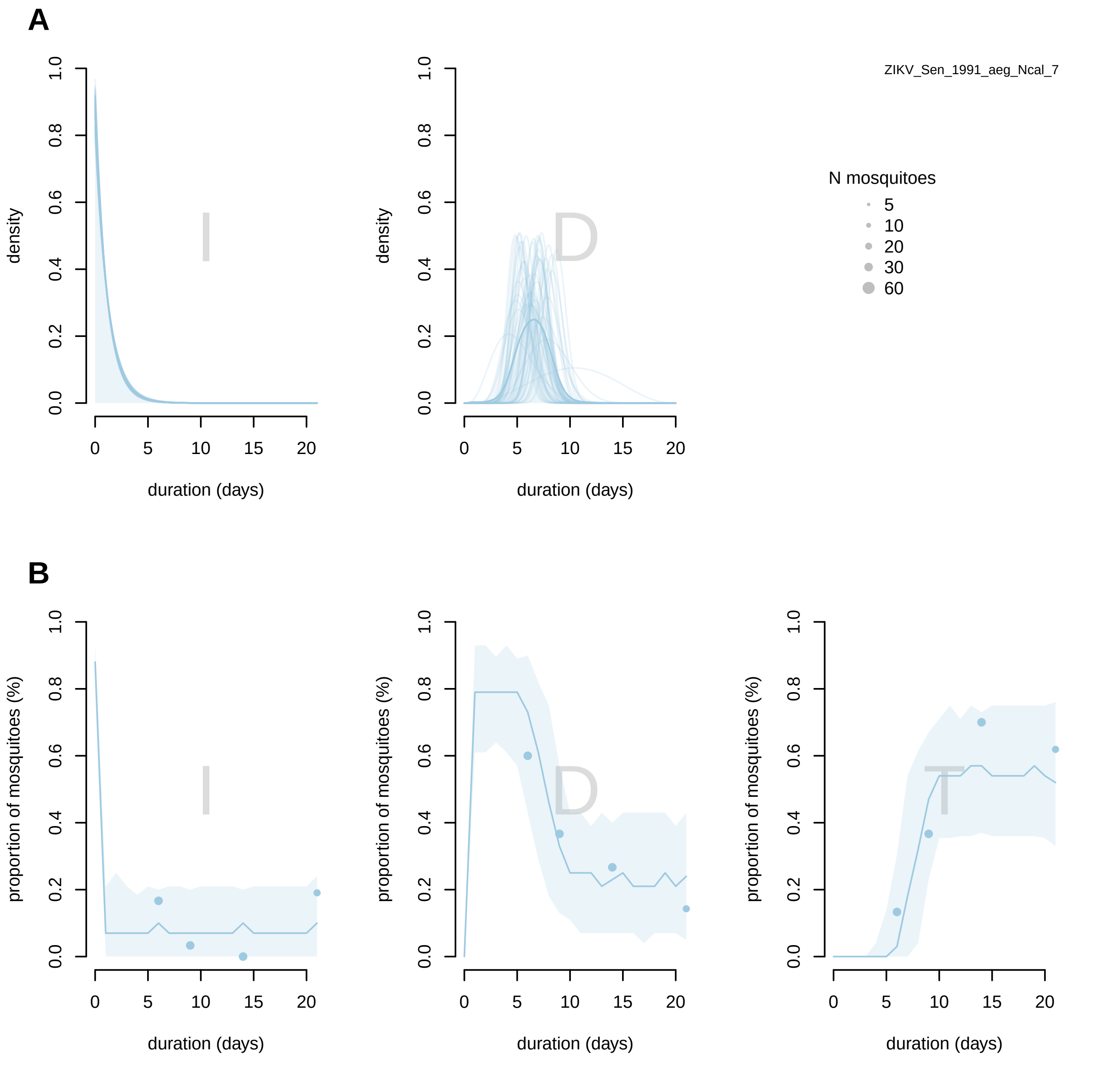

Fig AD. Inference results for IVD stages distributions for scenario ZIKVc11(ZIKV_Sen_1991_aeg_Ncal_7): *Aedes.aegypti* from New caledonia infected by Zika virus from Senegal (1991) with an infectious dose of 7 $\log_{10}$ TCID50/mL : A) Selected distributions in the infected and disseminated states for the main model selected. The dark line represents the mean of distributions and light lines represent a random sample of 50 distribution among all selected distribution. B) Selected dynamics in the infected (I), disseminated (D), and transmitter (T) states for the main model selected. The dots represent the observed data, the line (mean dynamics), and the uncertainty ribbons (5%-95%) represent selected simulated dynamic.

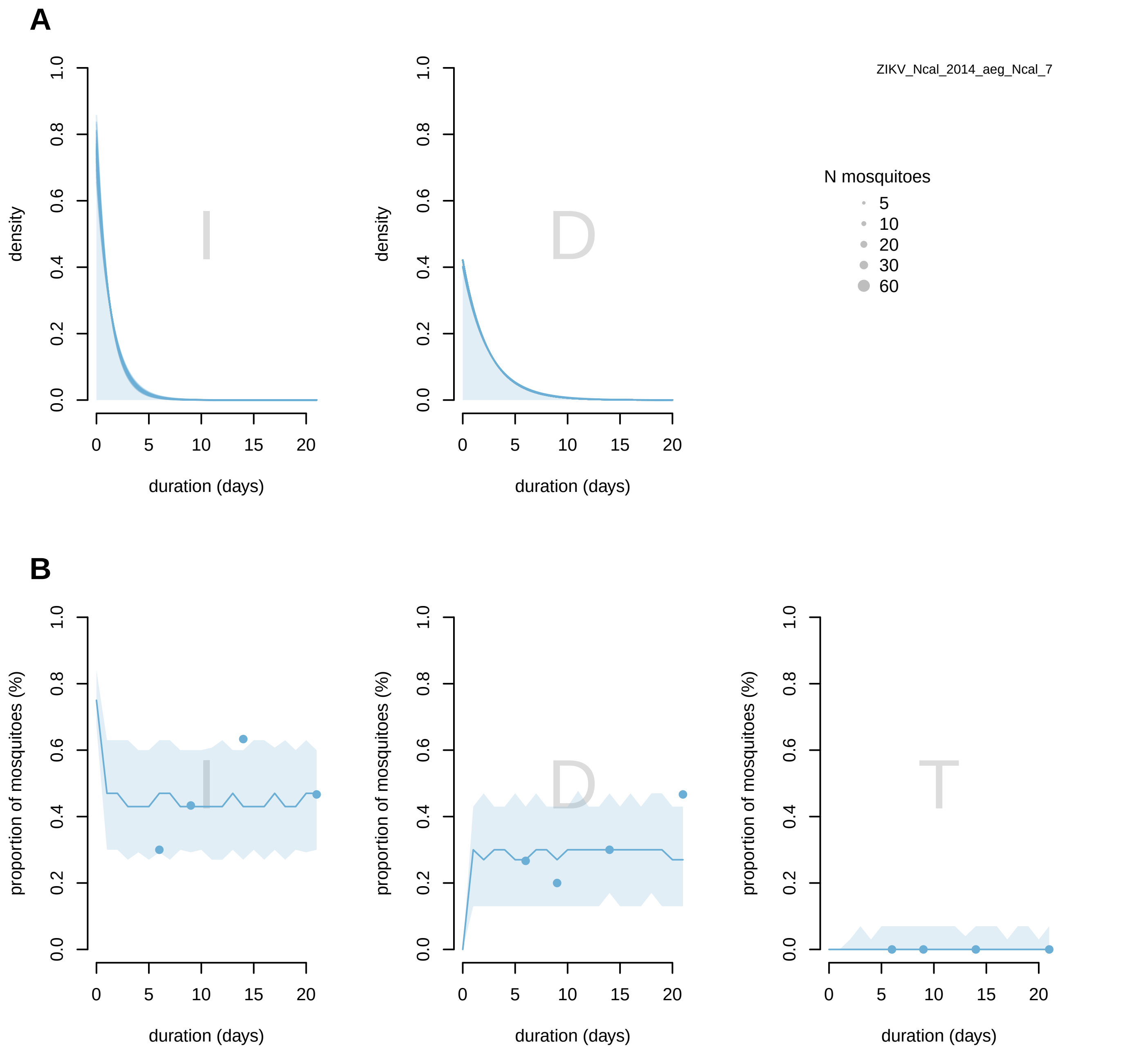

Fig AE. Inference results for IVD stages distributions for scenario ZIKVc12(ZIKV_Ncal_2014_aeg_Ncal_7): *Aedes.aegypti* from New caledonia infected by Zika virus (NC-2014-843) from New caledonia (2014) with an infectious dose of 7 $\log_{10}$ TCID50/mL : A) Selected distributions in the infected and disseminated states for the main model selected. The dark line represents the mean of distributions and light lines represent a random sample of 50 distribution among all selected distribution. B) Selected dynamics in the infected (I), disseminated (D), and transmitter (T) states for the main model selected. The dots represent the observed data, the line (mean dynamics), and the uncertainty ribbons (5%-95%) represent selected simulated dynamic.

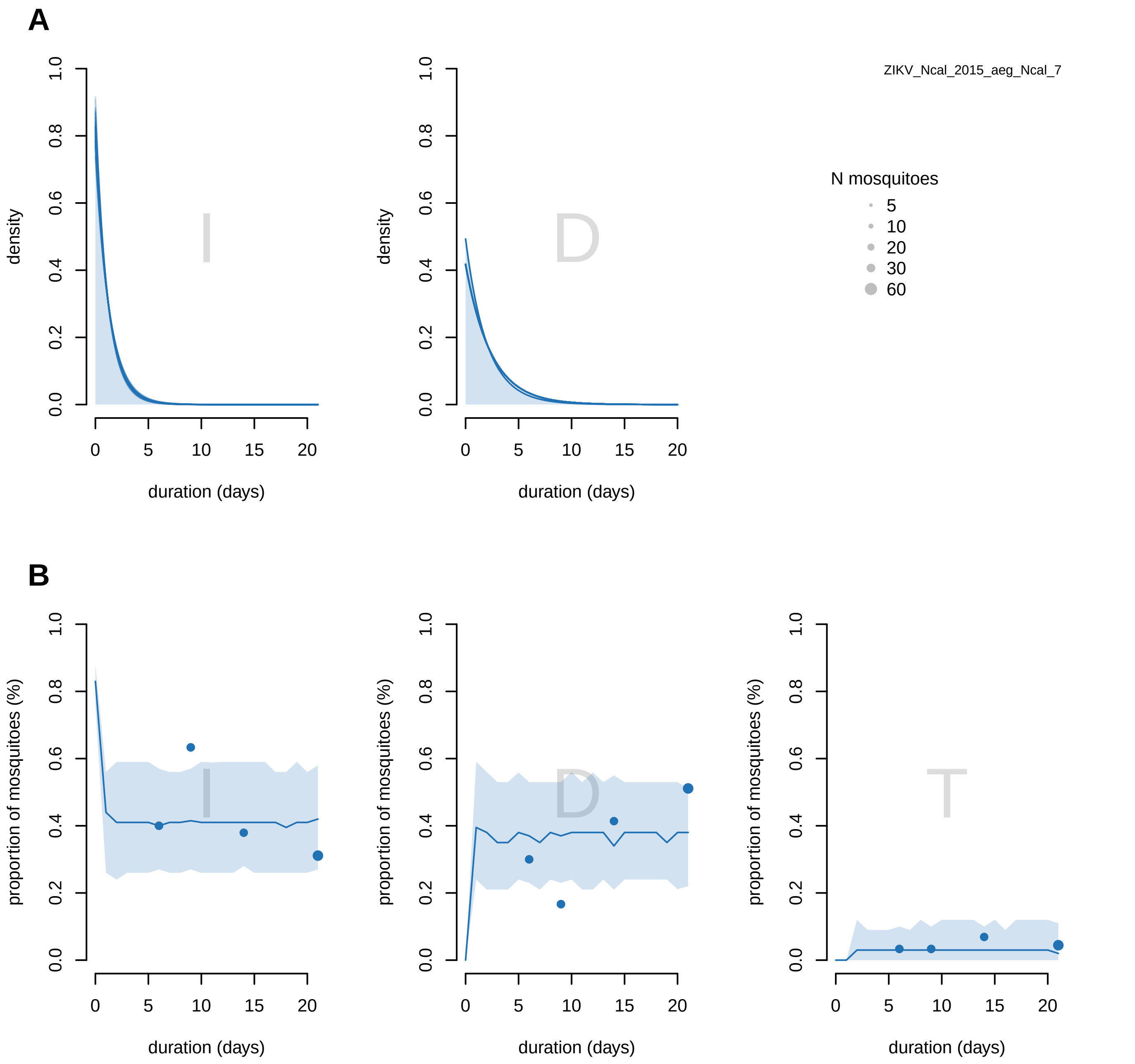

Fig AF. Inference results for IVD stages distributions for scenario ZIKVc13(ZIKV_Ncal_2015_aeg_Ncal_7): *Aedes.aegypti* from New caledonia infected by Zika virus from New caledonia(2015) with an infectious dose of 7 $\log_{10}$ TCID50/mL : A) Selected distributions in the infected and disseminated states for the main model selected. The dark line represents the mean of distributions and light lines represent a random sample of 50 distribution among all selected distribution. B) Selected dynamics in the infected (I), disseminated (D), and transmitter (T) states for the main model selected. The dots represent the observed data, the line (mean dynamics), and the uncertainty ribbons (5%-95%) represent selected simulated dynamic.

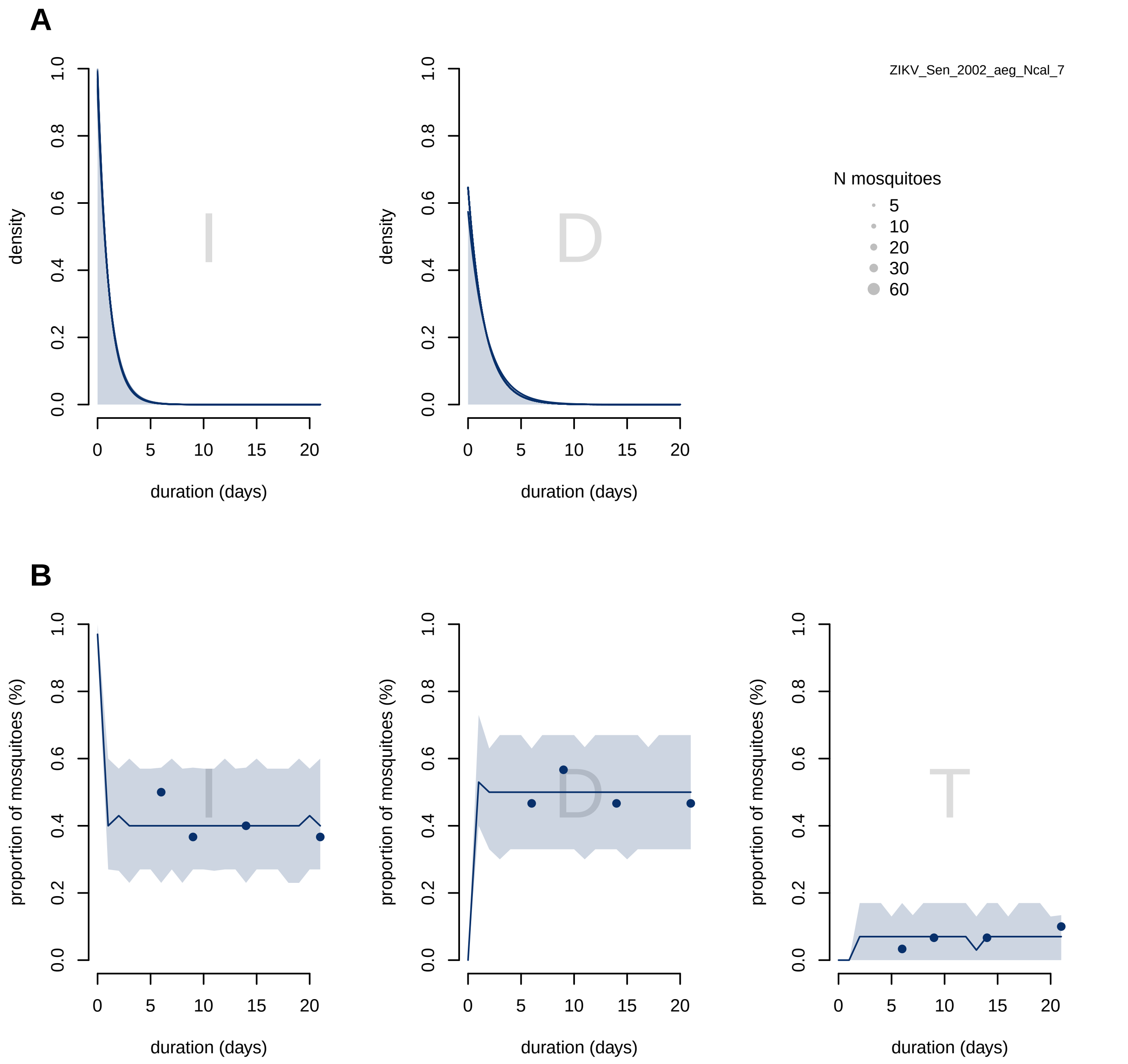

Fig AG. Inference results for IVD stages distributions for scenario ZIKVc14(ZIKV_Sen_2002_aeg_Ncal_7): *Aedes.aegypti* from New caledonia infected by Zika virus from Senegal(2002) with an infectious dose of 7 $\log_{10}$ TCID50/mL : A) Selected distributions in the infected and disseminated states for the main model selected. The dark line represents the mean of distributions and light lines represent a random sample of 50 distribution among all selected distribution. B) Selected dynamics in the infected (I), disseminated (D), and transmitter (T) states for the main model selected. The dots represent the observed data, the line (mean dynamics), and the uncertainty ribbons (5%-95%) represent selected simulated dynamic.

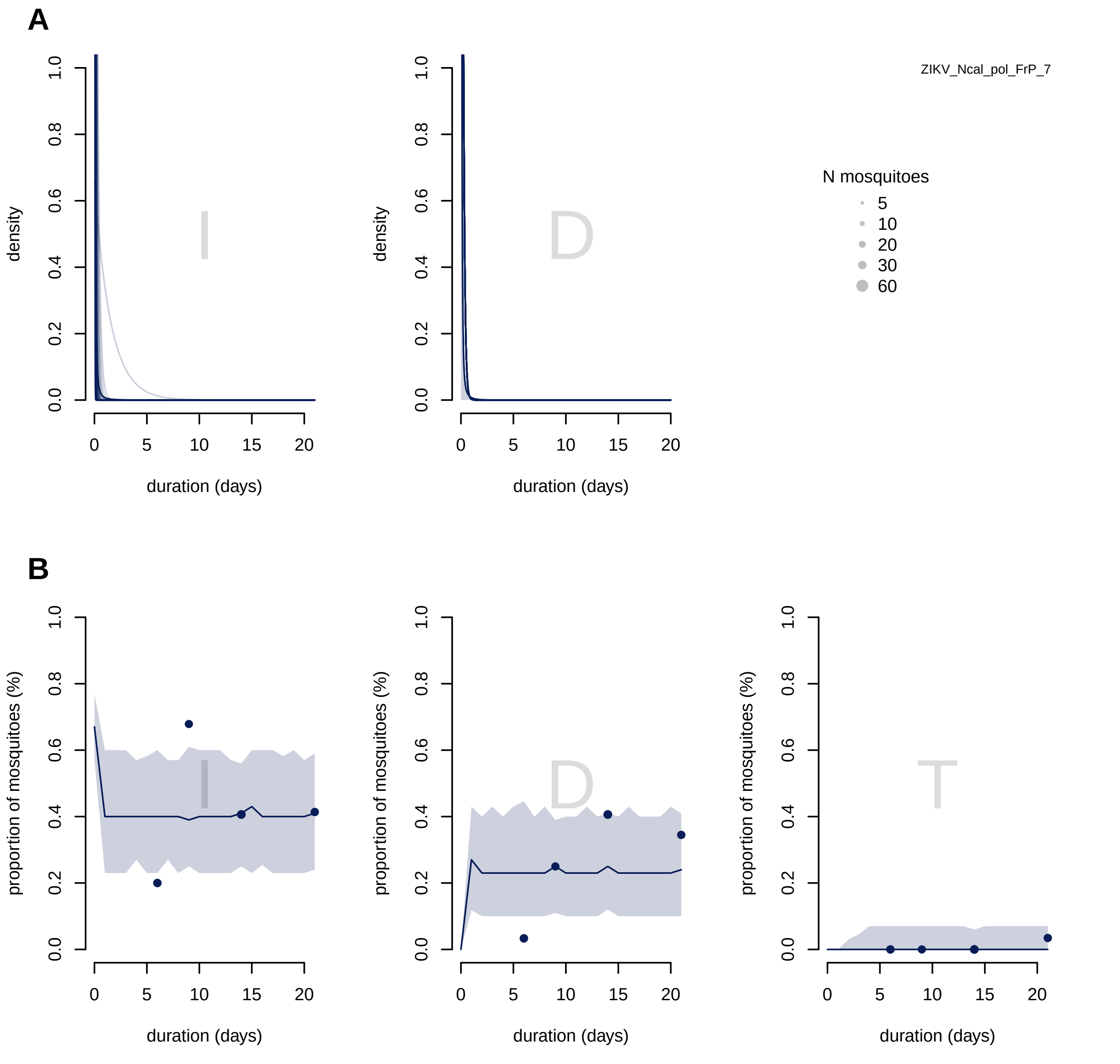

Fig AH. Inference results for IVD stages distributions for scenario ZIKVc15(ZIKV_Ncal_pol_FrP_7): *Aedes.polynesiensis* from French Polynesia infected by Zika virus from New Caledonia with an infectious dose of 7 $\log_{10}$ TCID50/mL : A) Selected distributions in the infected and disseminated states for the main model selected. The dark line represents the mean of distributions and light lines represent a random sample of 50 distribution among all selected distribution. B) Selected dynamics in the infected (I), disseminated (D), and transmitter (T) states for the main model selected. The dots represent the observed data, the line (mean dynamics), and the uncertainty ribbons (5%-95%) represent selected simulated dynamic.

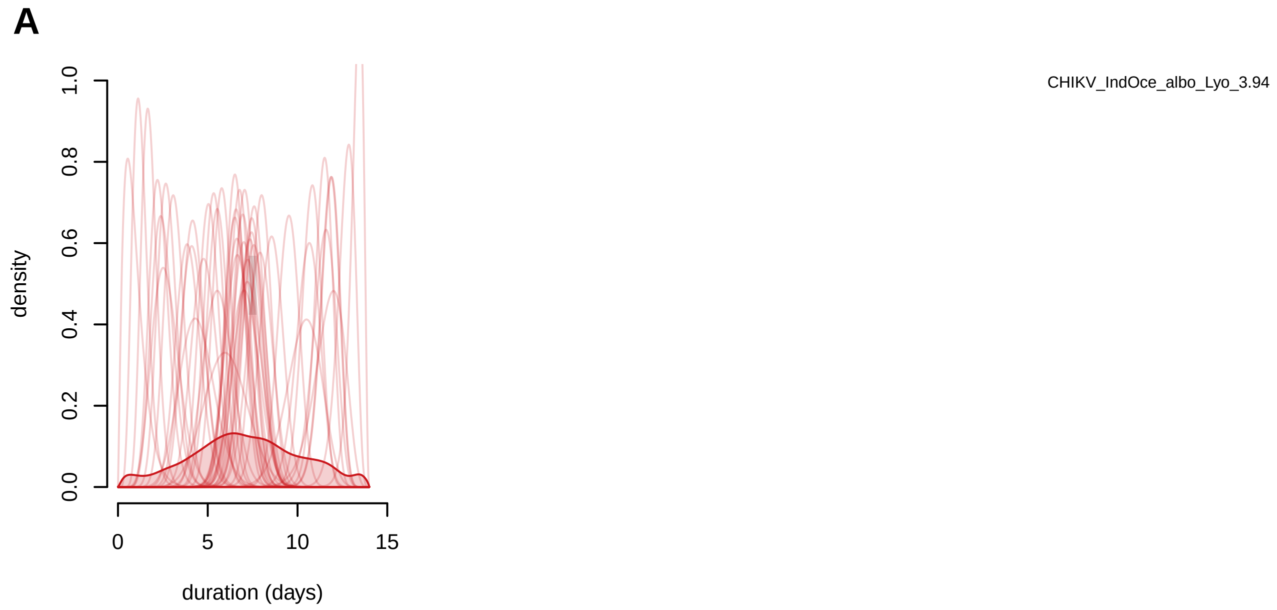

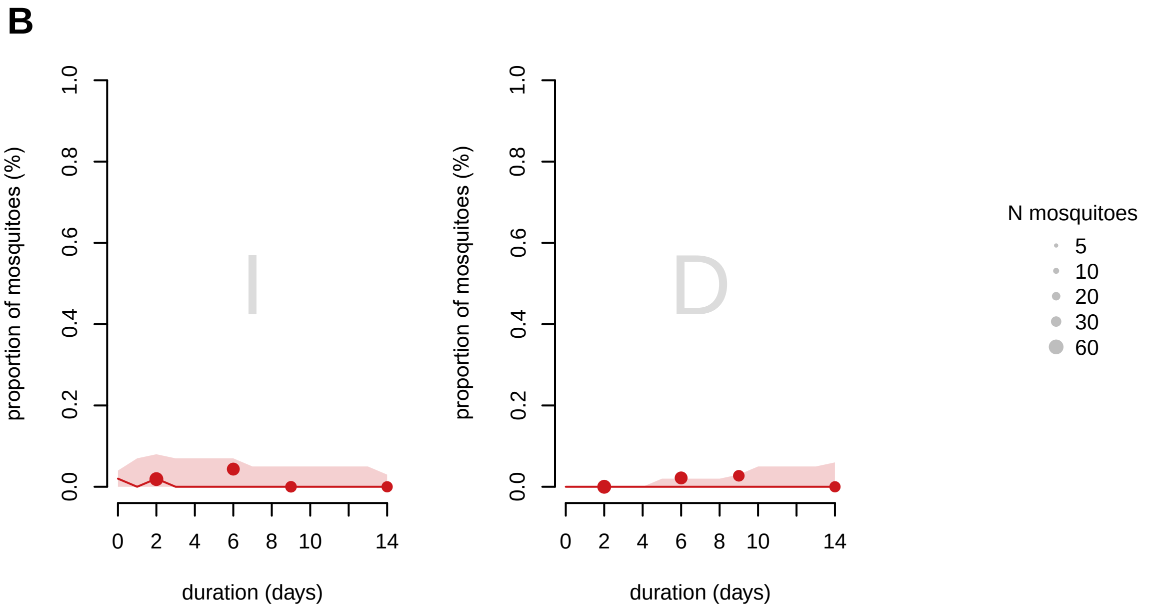

Fig AI. Inference results for IVD stages distributions for scenario CHIKVp1(CHIKV_IndOce_albo_Lyo_3.94): *Aedes.albopictus* from Lyon infected by chikungunya virus from Indian Ocean with an infectious dose of 3.94 log10 FFU/mL : A) Selected distributions in the infected state for the main model selected. The dark line represents the mean of distributions and light lines represent a random sample of 50 distribution among all selected distribution. B) Selected dynamics in the infected (I) and disseminated (D) states for the main model selected. The dots represent the observed data, the line (mean dynamics), and the uncertainty ribbons (5%-95%) represent selected simulated dynamic.

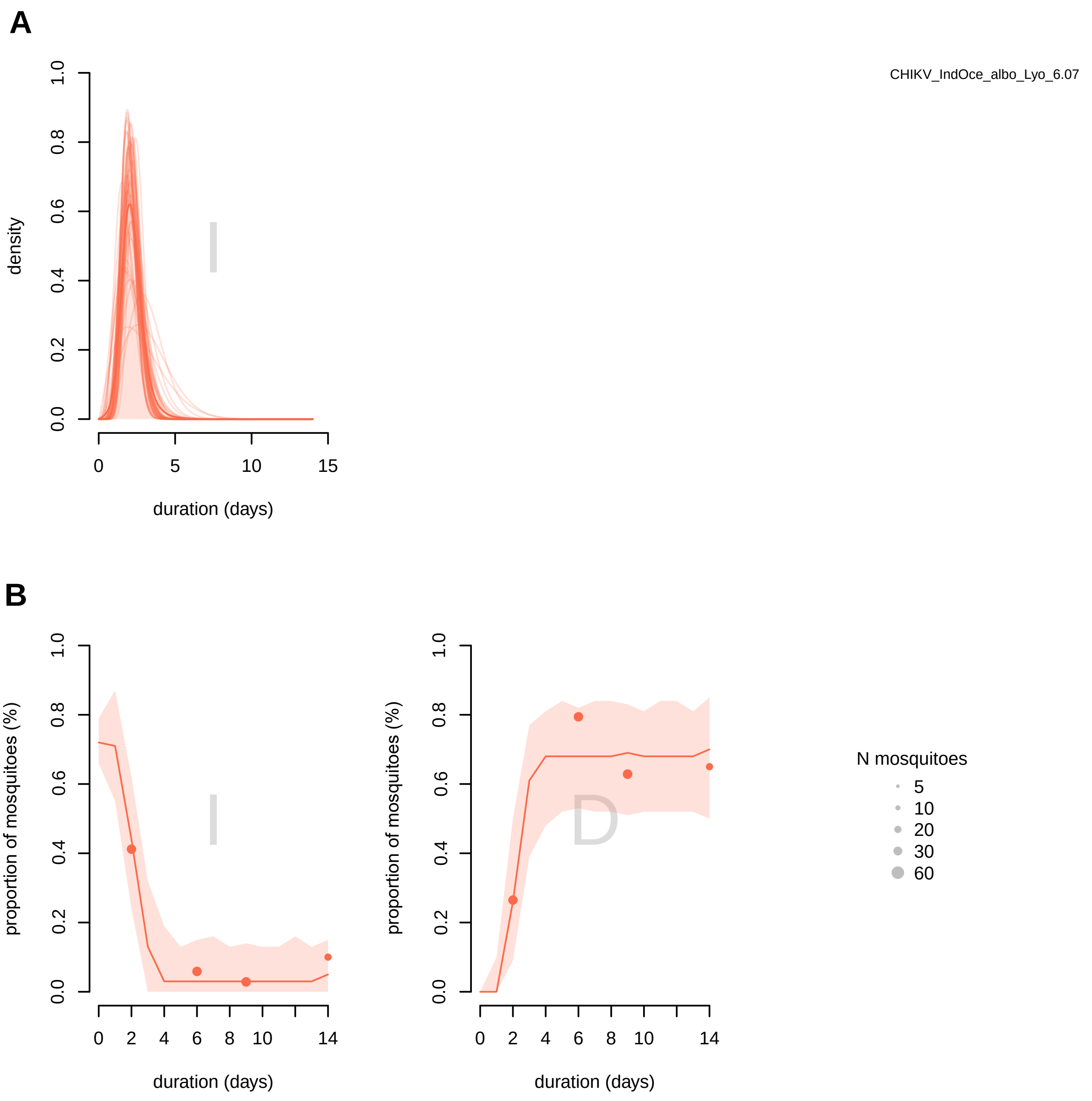

Fig AJ. Inference results for IVD stages distributions for scenario CHIKVp2(CHIKV_IndOce_albo_Lyo_6.07): *Aedes.albopictus* from Lyon infected by chikungunya virus from Indian Ocean with an infectious dose of 6.07 log10 FFU/mL : A) Selected distributions in the infected state for the main model selected. The dark line represents the mean of distributions and light lines represent a random sample of 50 distribution among all selected distribution. B) Selected dynamics in the infected (I) and disseminated (D) states for the main model selected. The dots represent the observed data, the line (mean dynamics), and the uncertainty ribbons (5%-95%) represent selected simulated dynamic.

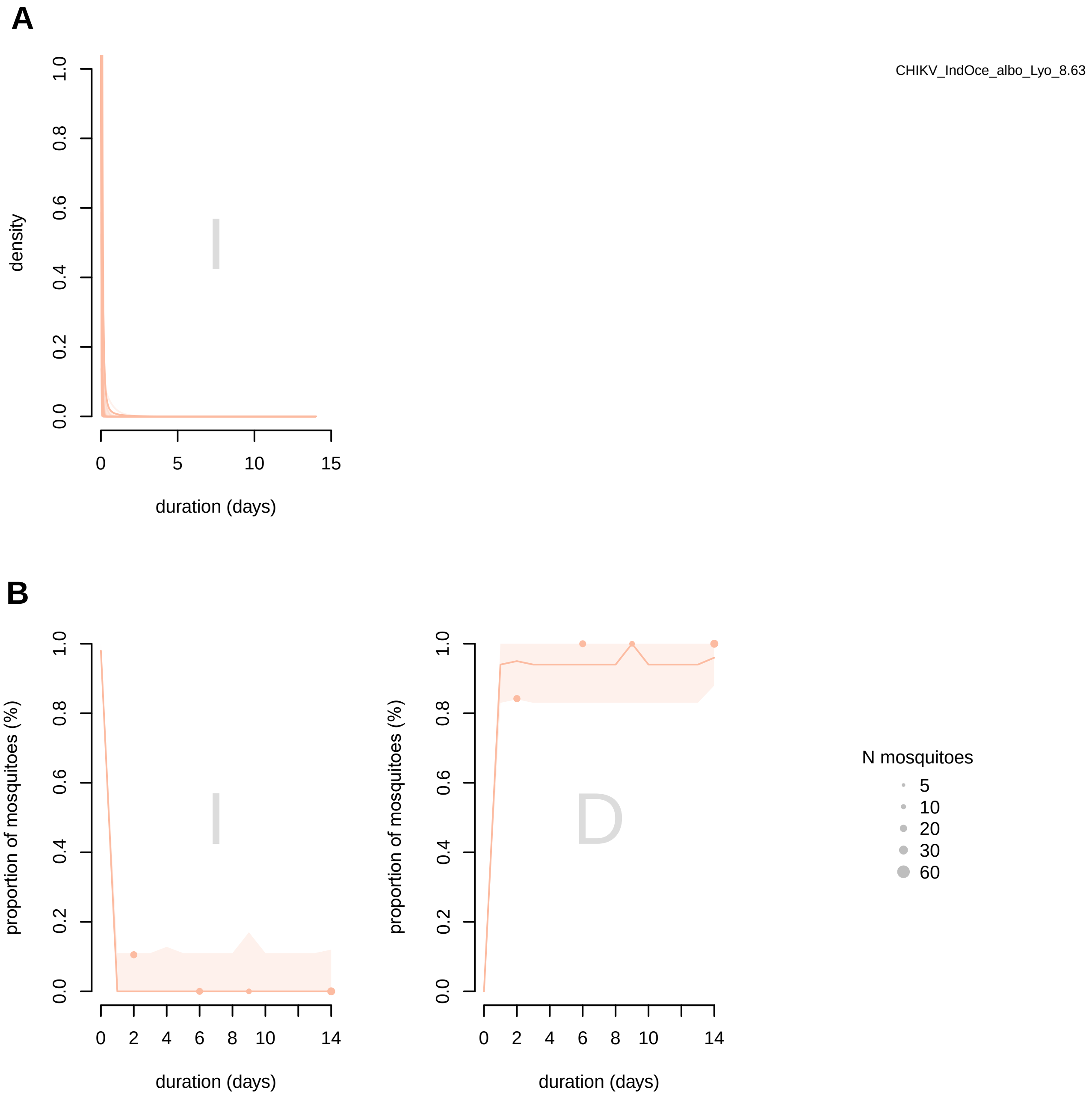

Fig AK. Inference results for IVD stages distributions for scenario CHIKVp3(CHIKV_IndOce_albo_Lyo_8.63): *Aedes.albopictus* from Lyon infected by chikungunya virus from Indian Ocean with an infectious dose of 8.63 log10 FFU/mL : A) Selected distributions in the infected state for the main model selected. The dark line represents the mean of distributions and light lines represent a random sample of 50 distribution among all selected distribution. B) Selected dynamics in the infected (I) and disseminated (D) states for the main model selected. The dots represent the observed data, the line (mean dynamics), and the uncertainty ribbons (5%-95%) represent selected simulated dynamic.

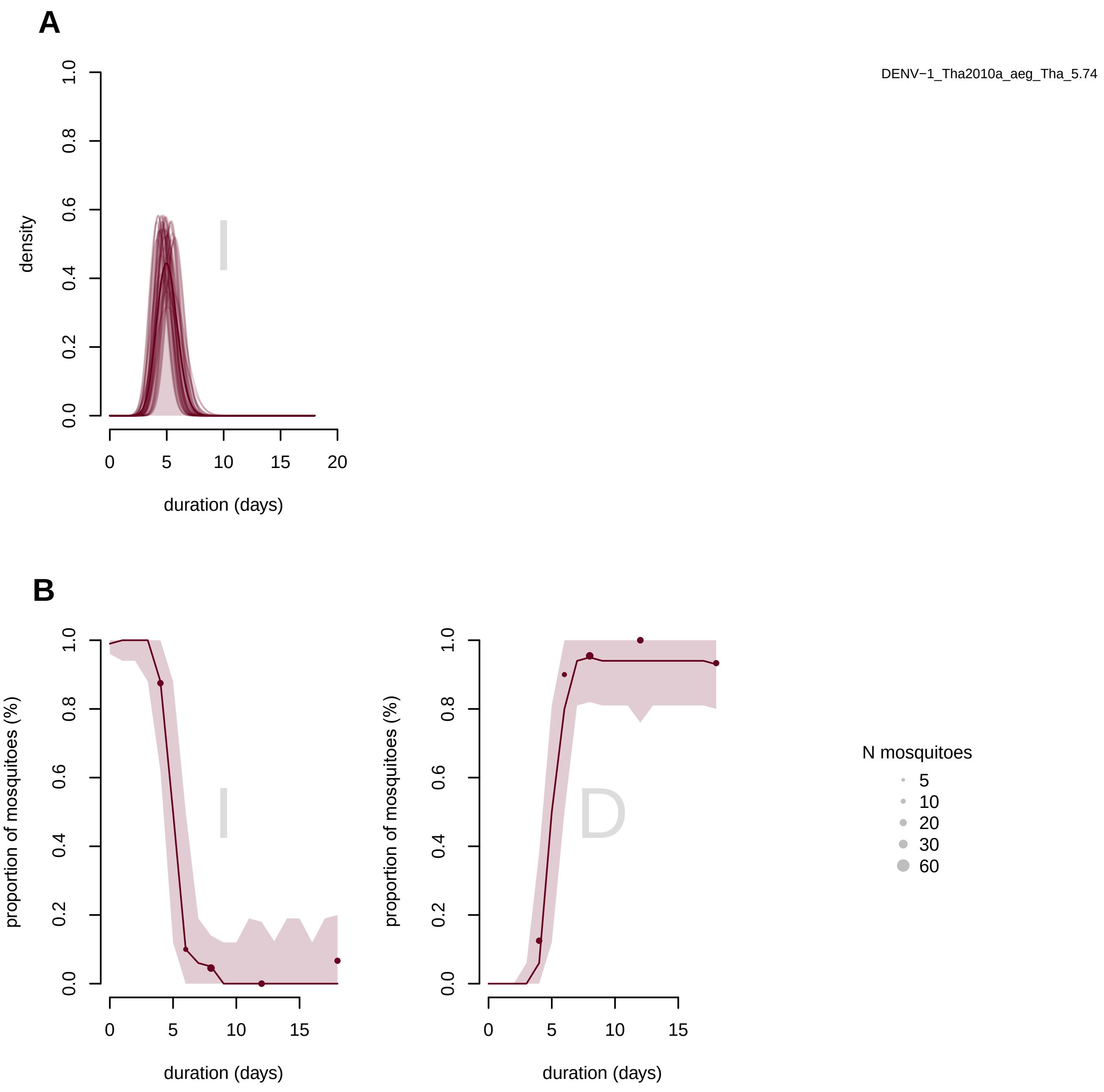

Fig AL. Inference results for IVD stages distributions for scenario DENVp1(DENV1_Tha2010a_aeg_Tha_5.74): *Aedes.aegypti* from Thailand infected by dengue virus from Thailand with an infectious dose of 5.74 log10 FFU/mL : A) Selected distributions in the infected state for the main model selected. The dark line represents the mean of distributions and light lines represent a random sample of 50 distribution among all selected distribution. B) Selected dynamics in the infected (I) and disseminated (D) states for the main model selected. The dots represent the observed data, the line (mean dynamics), and the uncertainty ribbons (5%-95%) represent selected simulated dynamic.

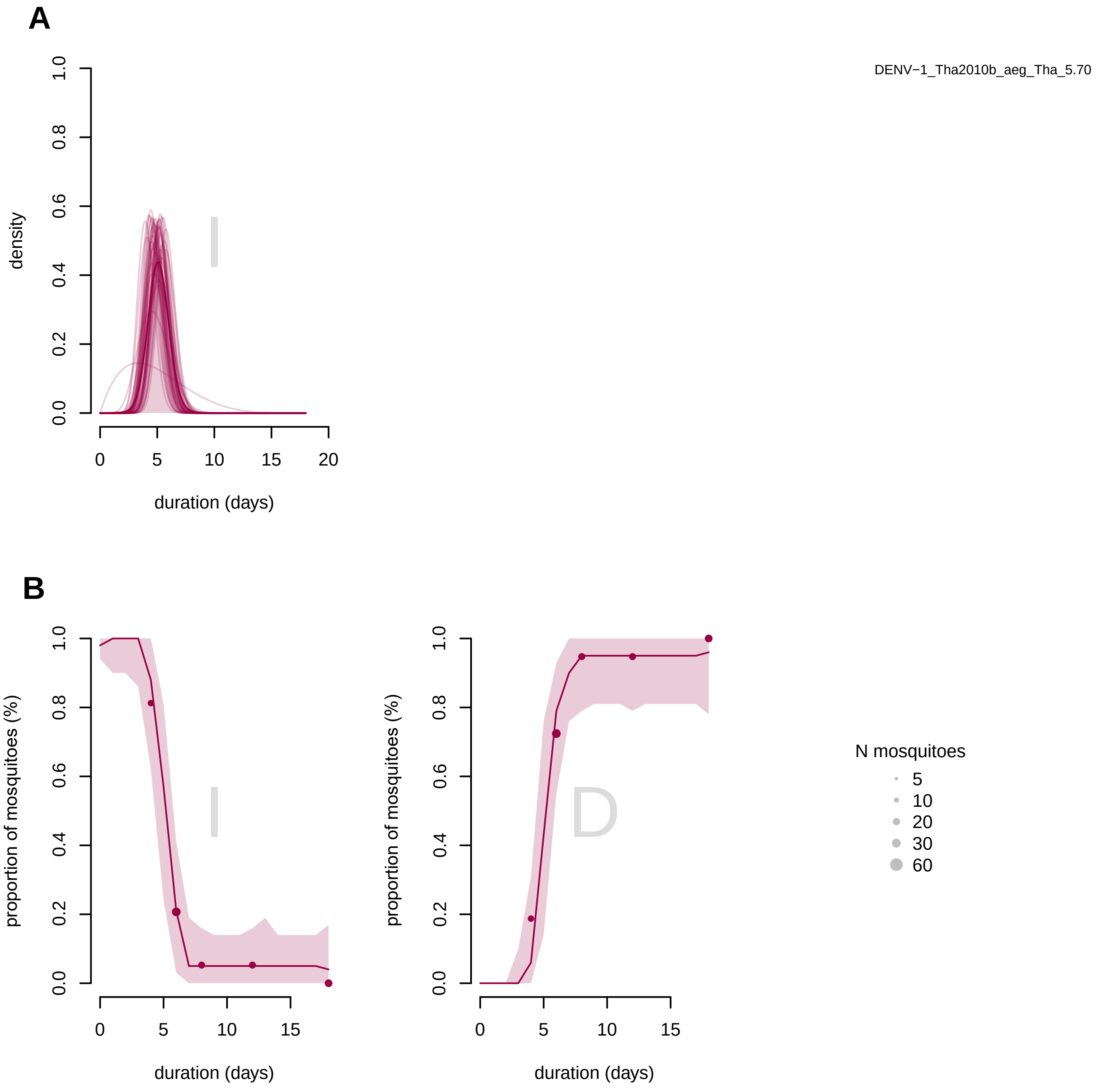

Fig AM. Inference results for IVD stages distributions for scenario DENVp2(DENV1_Tha2010b_aeg_Tha_5.70): *Aedes.aegypti* from Thailand infected by dengue virus from Thailand with an infectious dose of 5.70 log10 FFU/mL : A) Selected distributions in the infected state for the main model selected. The dark line represents the mean of distributions and light lines represent a random sample of 50 distribution among all selected distribution. B) Selected dynamics in the infected (I) and disseminated (D) states for the main model selected. The dots represent the observed data, the line (mean dynamics), and the uncertainty ribbons (5%-95%) represent selected simulated dynamic.

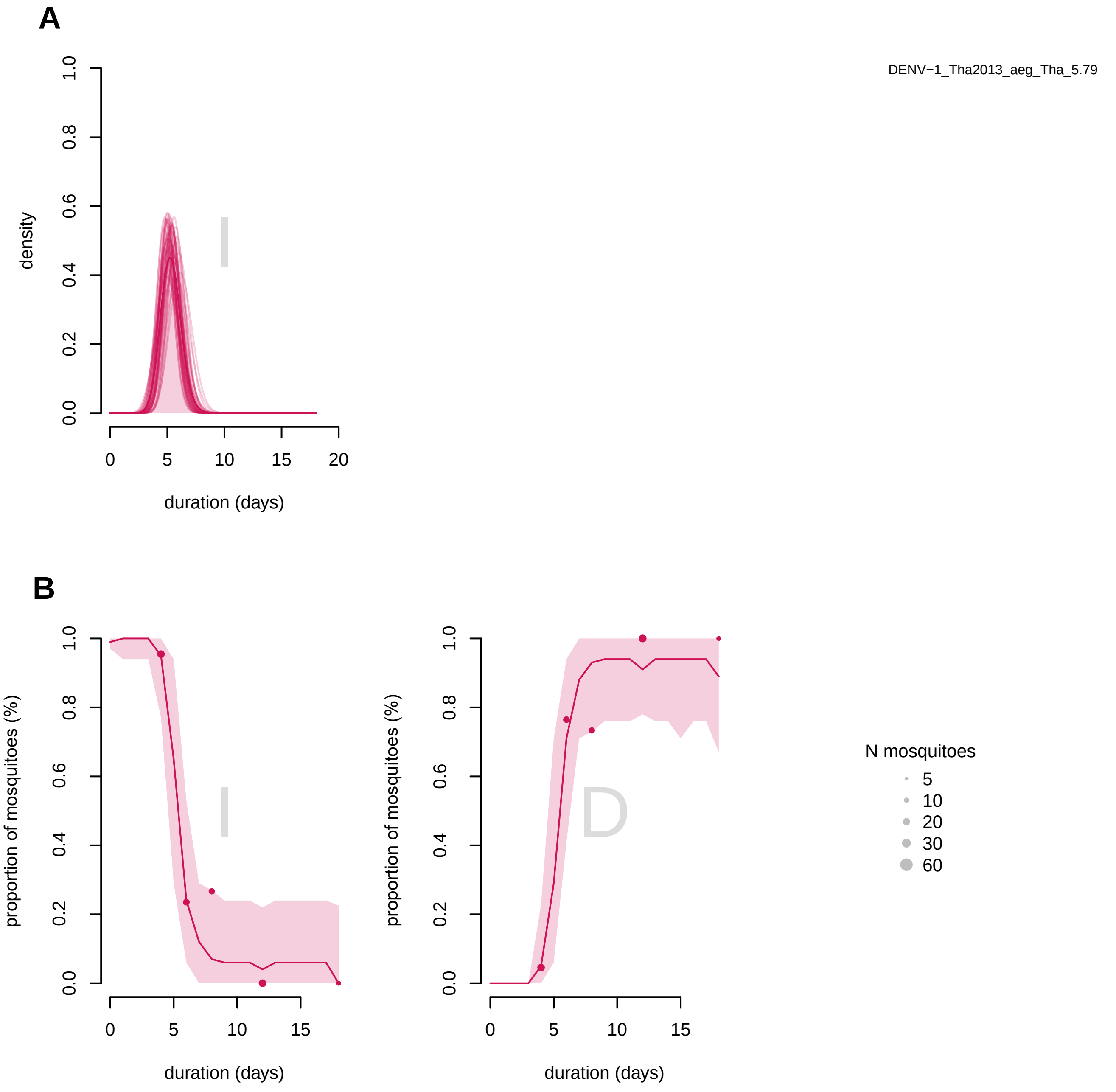

Fig AN Inference results for IVD stages distributions for scenario DENVp3(DENV1_Tha2013_aeg_Tha_5.79): *Aedes.aegypti* from Thailand infected by dengue virus from Thailand with an infectious dose of 5.79 log10 FFU/mL : A) Selected distributions in the infected state for the main model selected. The dark line represents the mean of distributions and light lines represent a random sample of 50 distribution among all selected distribution. B) Selected dynamics in the infected (I) and disseminated (D) states for the main model selected. The dots represent the observed data, the line (mean dynamics), and the uncertainty ribbons (5%-95%) represent selected simulated dynamic.

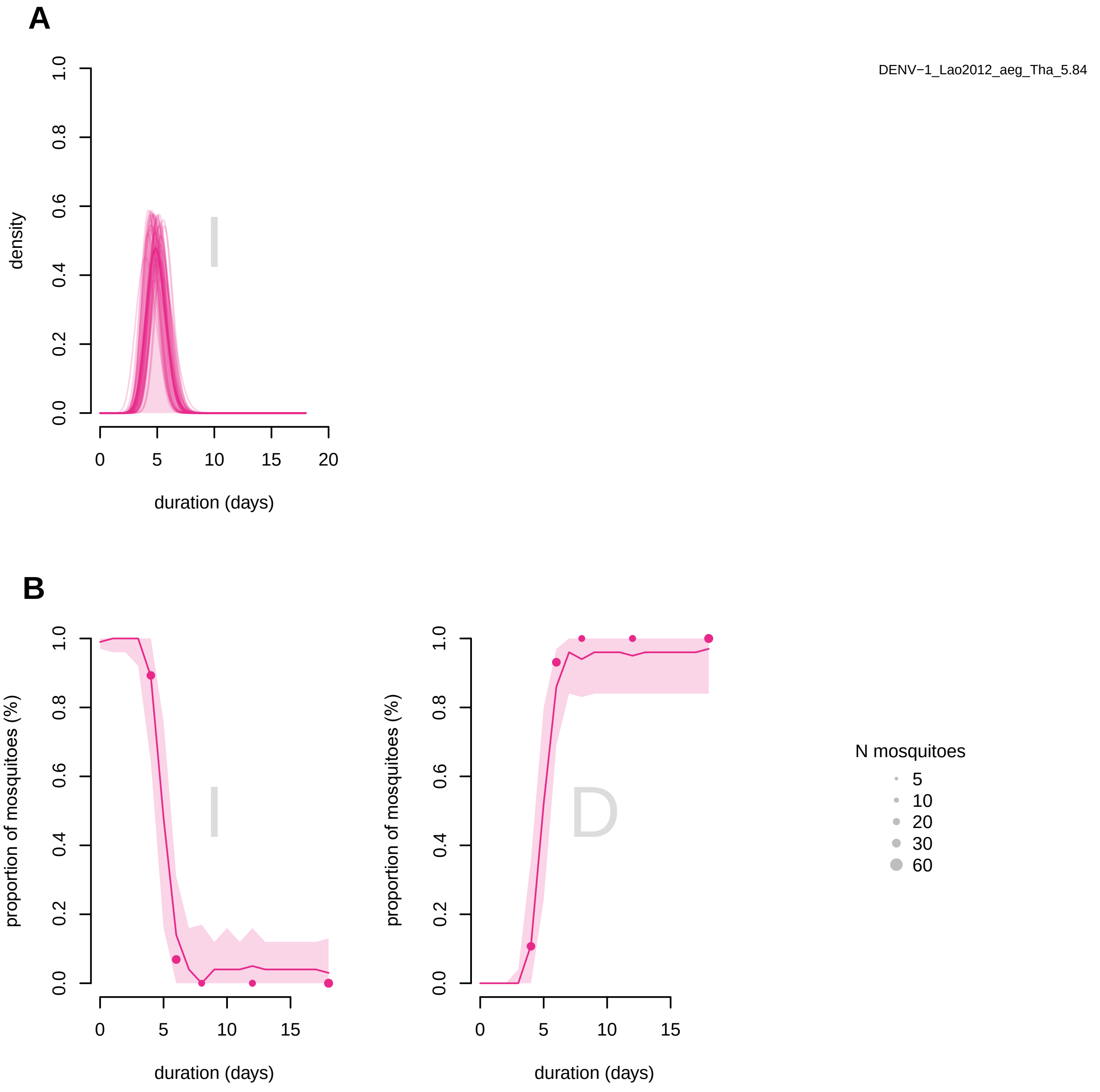

Fig AO. Inference results for IVD stages distributions for scenario DENVp4(DENV1_Lao2012_aeg_Tha_5.84): *Aedes.aegypti* from Thailand infected by dengue virus from Laos with an infectious dose of 5.84 log10 FFU/mL : A) Selected distributions in the infected state for the main model selected. The dark line represents the mean of distributions and light lines represent a random sample of 50 distribution among all selected distribution. B) Selected dynamics in the infected (I) and disseminated (D) states for the main model selected. The dots represent the observed data, the line (mean dynamics), and the uncertainty ribbons (5%-95%) represent selected simulated dynamic.

A

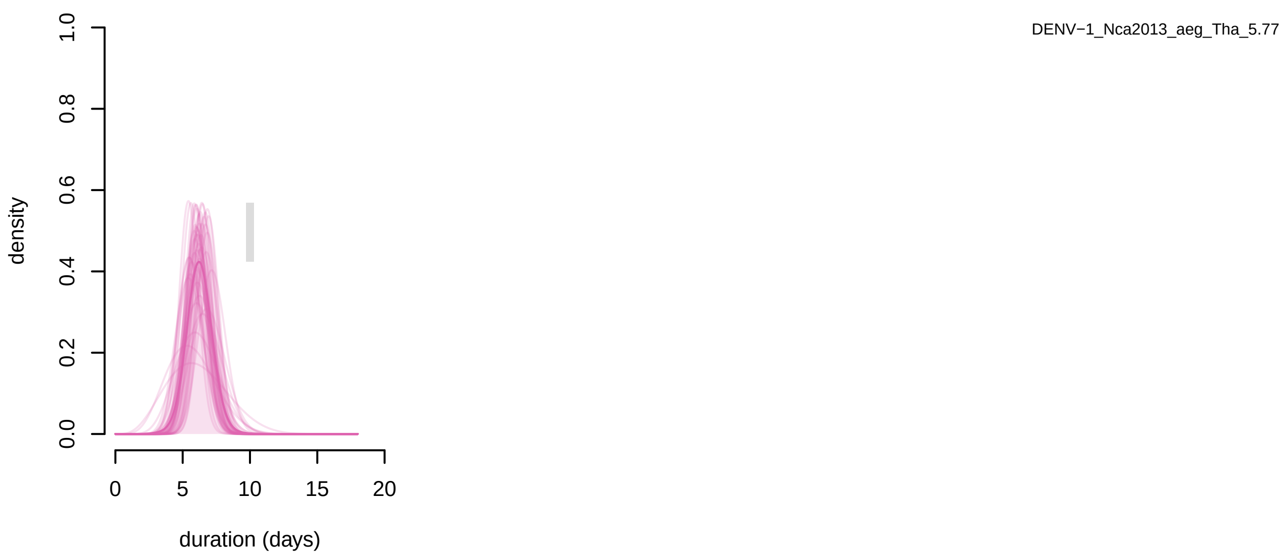

B

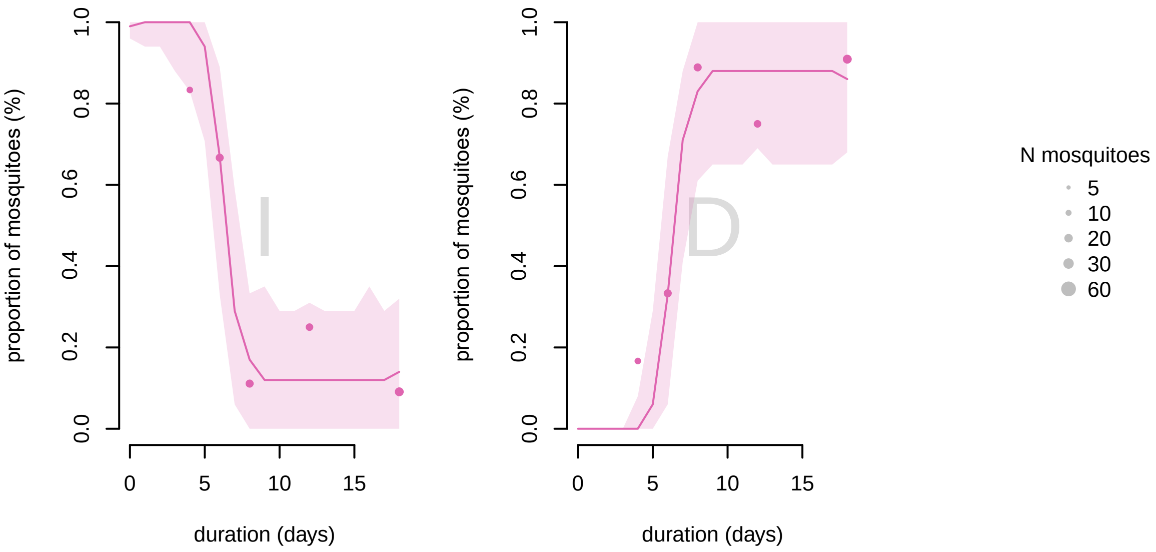

Fig AP. Inference results for IVD stages distributions for scenario DENVp5(DENV1_Nca2013_aeg_Tha_5.77): *Aedes.aegypti* from Thailand infected by dengue virus from New Caledonia with an infectious dose of 5.77 log10 FFU/mL : A) Selected distributions in the infected state for the main model selected. The dark line represents the mean of distributions and light lines represent a random sample of 50 distribution among all selected distribution. B) Selected dynamics in the infected (I) and disseminated (D) states for the main model selected. The dots represent the observed data, the line (mean dynamics), and the uncertainty ribbons (5%-95%) represent selected simulated dynamic.

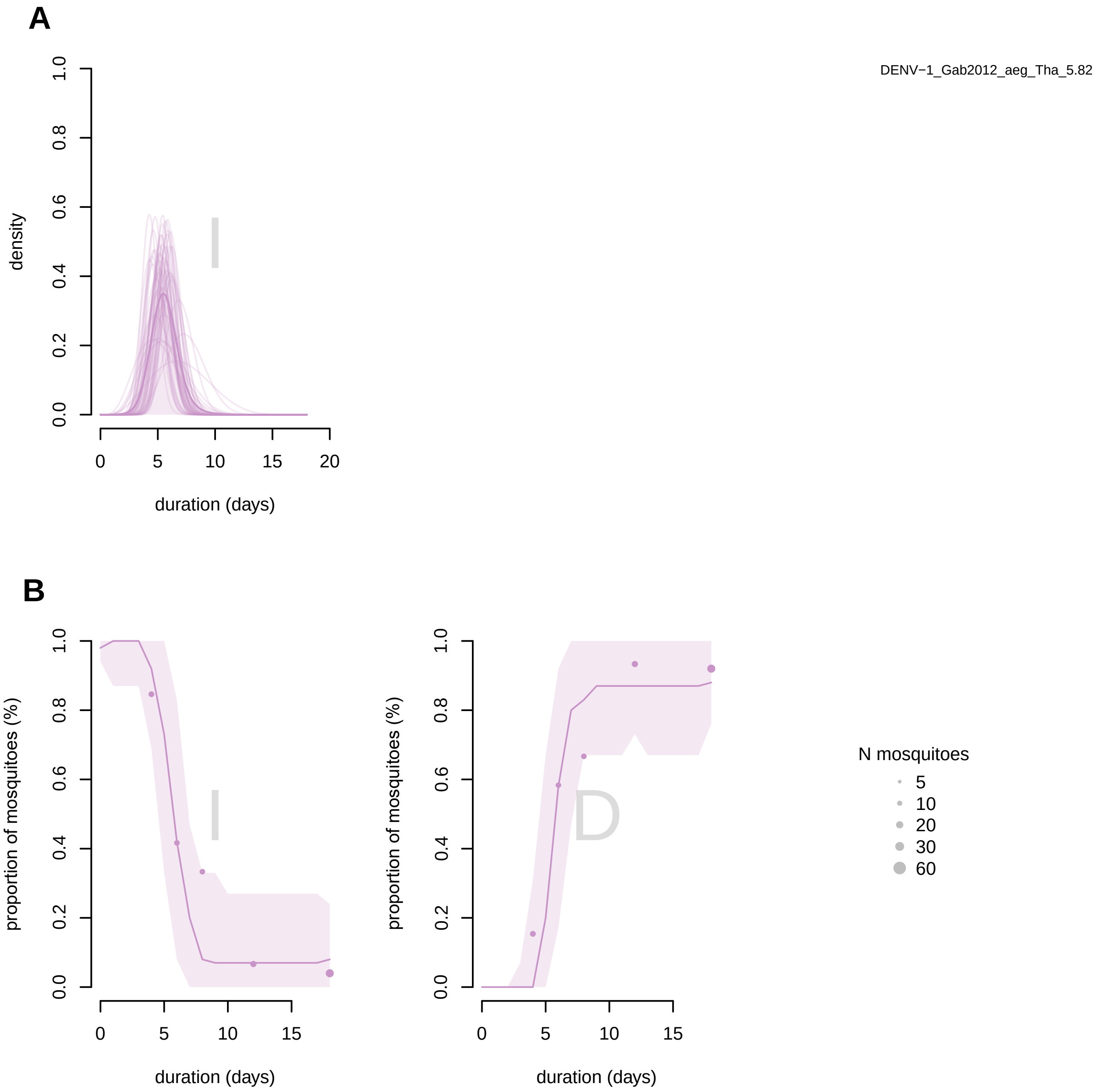

Fig AQ. Inference results for IVD stages distributions for scenario DENVp6(DENV1_Gab2012_aeg_Tha_5.82): *Aedes.aegypti* from Thailand infected by dengue virus from Gabon with an infectious dose of 5.82 log10 FFU/mL : A) Selected distributions in the infected state for the main model selected. The dark line represents the mean of distributions and light lines represent a random sample of 50 distribution among all selected distribution. B) Selected dynamics in the infected (I) and disseminated (D) states for the main model selected. The dots represent the observed data, the line (mean dynamics), and the uncertainty ribbons (5%-95%) represent selected simulated dynamic.

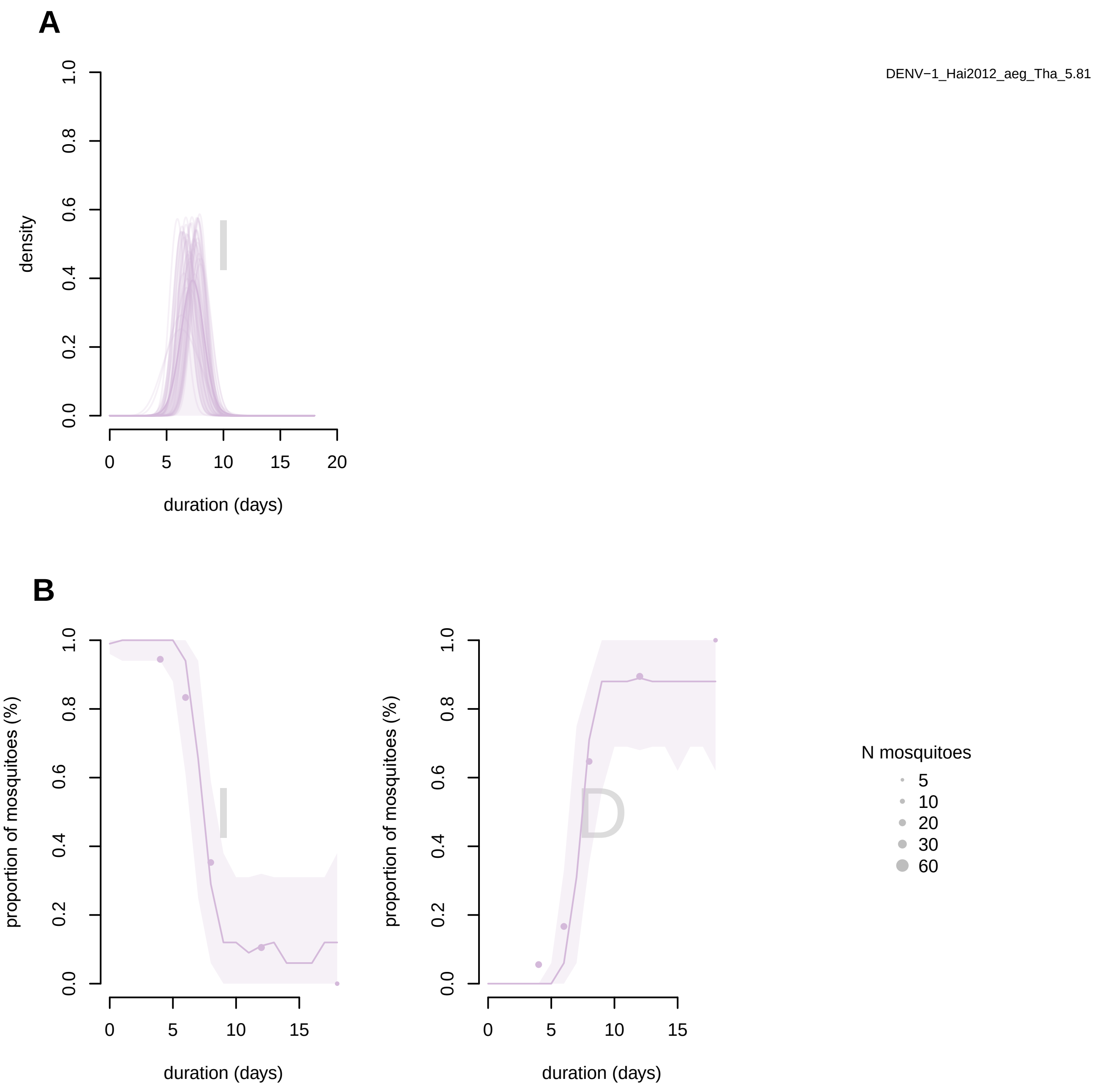

Fig AR. Inference results for IVD stages distributions for scenario DENVp7(DENV1_Hai2012_aeg_Tha_5.81): *Aedes.aegypti* from Thailand infected by dengue virus from Haïti with an infectious dose of 5.81 log10 FFU/mL : A) Selected distributions in the infected state for the main model selected. The dark line represents the mean of distributions and light lines represent a random sample of 50 distribution among all selected distribution. B) Selected dynamics in the infected (I) and disseminated (D) states for the main model selected. The dots represent the observed data, the line (mean dynamics), and the uncertainty ribbons (5%-95%) represent selected simulated dynamic.

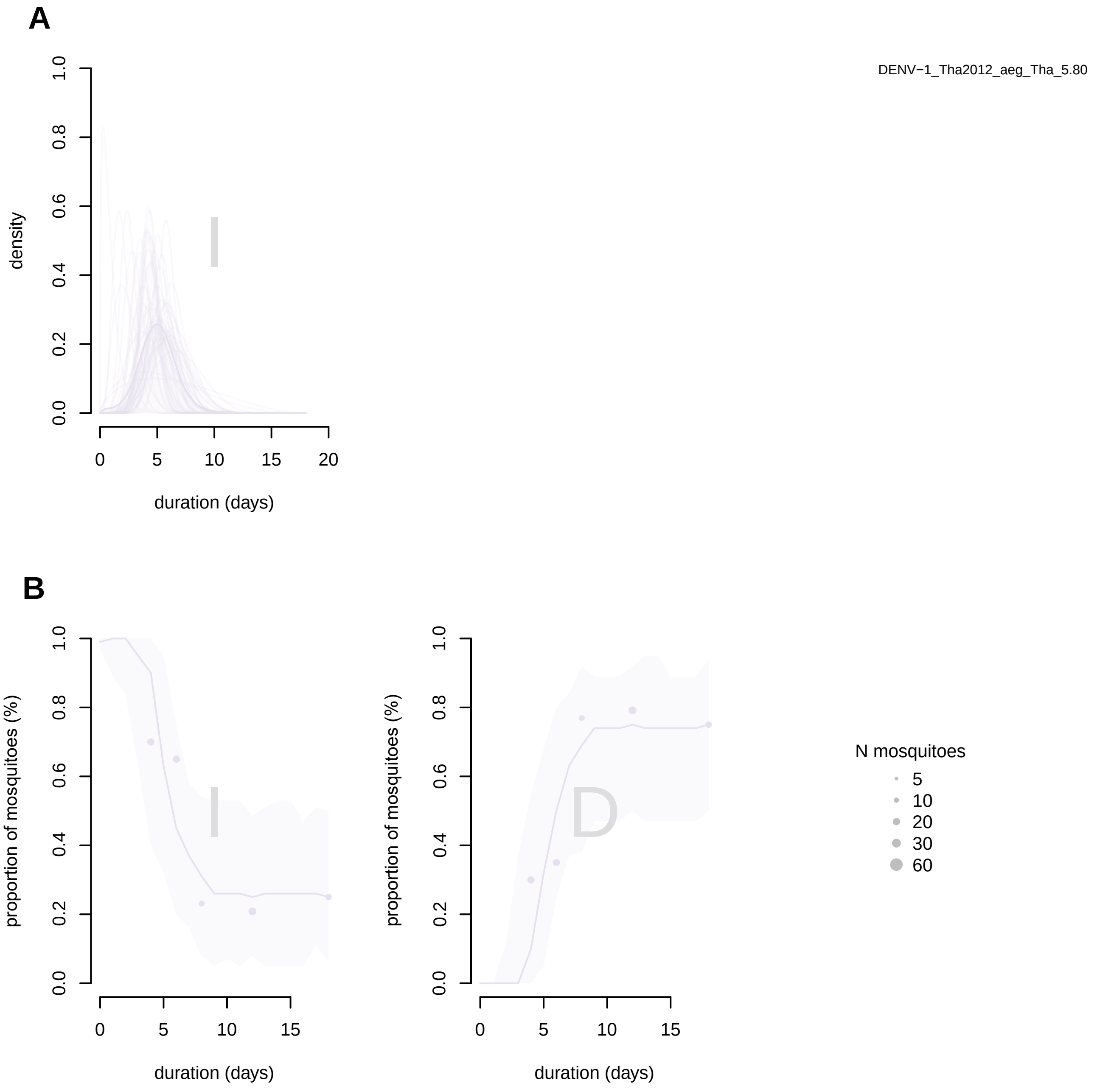

Fig AS. Inference results for IVD stages distributions for scenario DENVp8(DENV1_Tha2012_aeg_Tha_5.80): *Aedes.aegypti* from Thailand infected by dengue virus from Thailand with an infectious dose of 5.80 log10 FFU/mL : A) Selected distributions in the infected state for the main model selected. The dark line represents the mean of distributions and light lines represent a random sample of 50 distribution among all selected distribution. B) Selected dynamics in the infected (I) and disseminated (D) states for the main model selected. The dots represent the observed data, the line (mean dynamics), and the uncertainty ribbons (5%-95%) represent selected simulated dynamic.

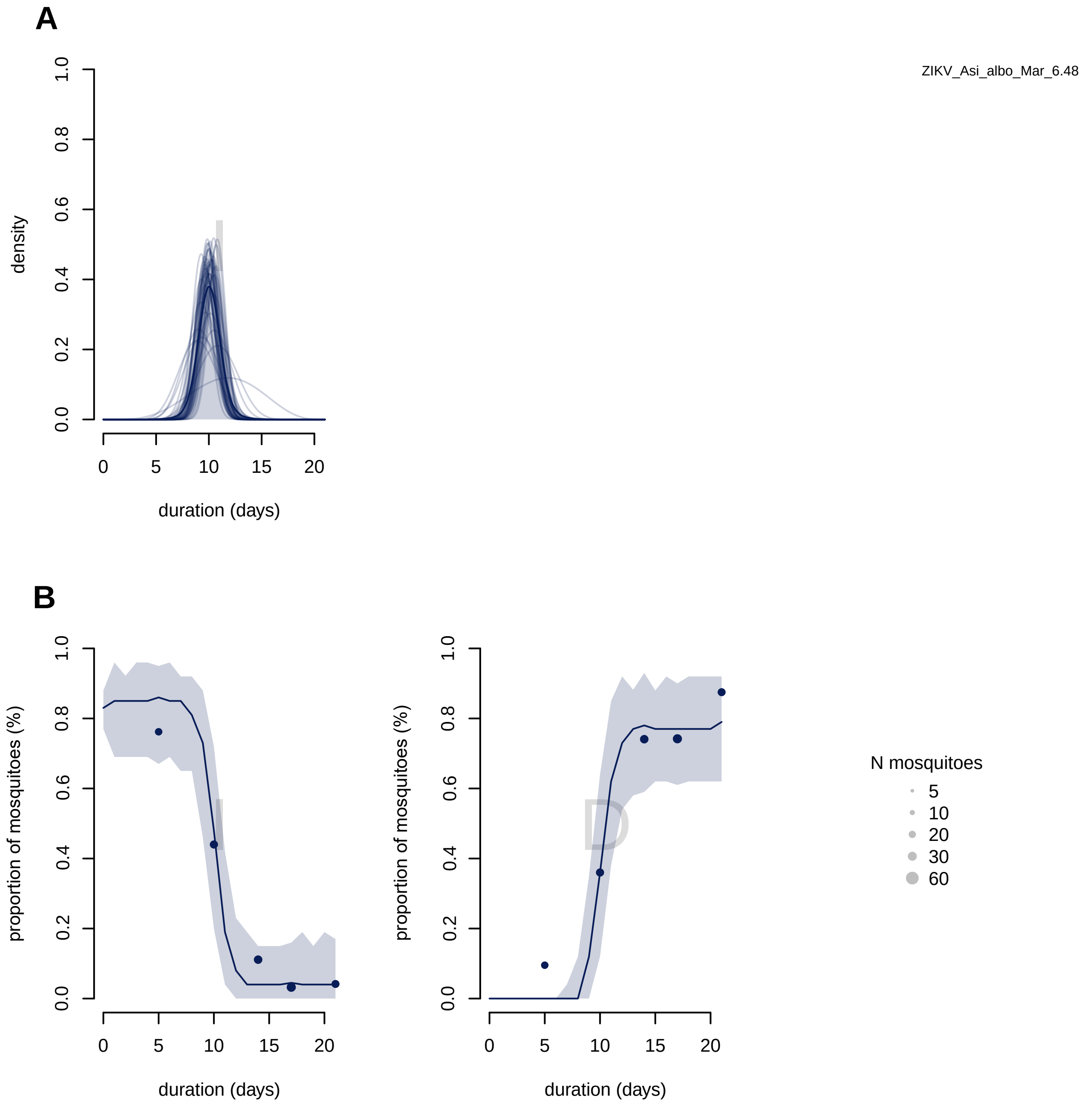

Fig AT. Inference results for IVD stages distributions for scenario ZIKVp1(ZIKV_Asi_albo_Mar_6.48): *Aedes.albopictus* from Marseille infected by Zika virus from Asia with an infectious dose of 6.48 log10 FFU/mL : A) Selected distributions in the infected state for the main model selected. The dark line represents the mean of distributions and light lines represent a random sample of 50 distribution among all selected distribution. B) Selected dynamics in the infected (I) and disseminated (D) states for the main model selected. The dots represent the observed data, the line (mean dynamics), and the uncertainty ribbons (5%-95%) represent selected simulated dynamic.

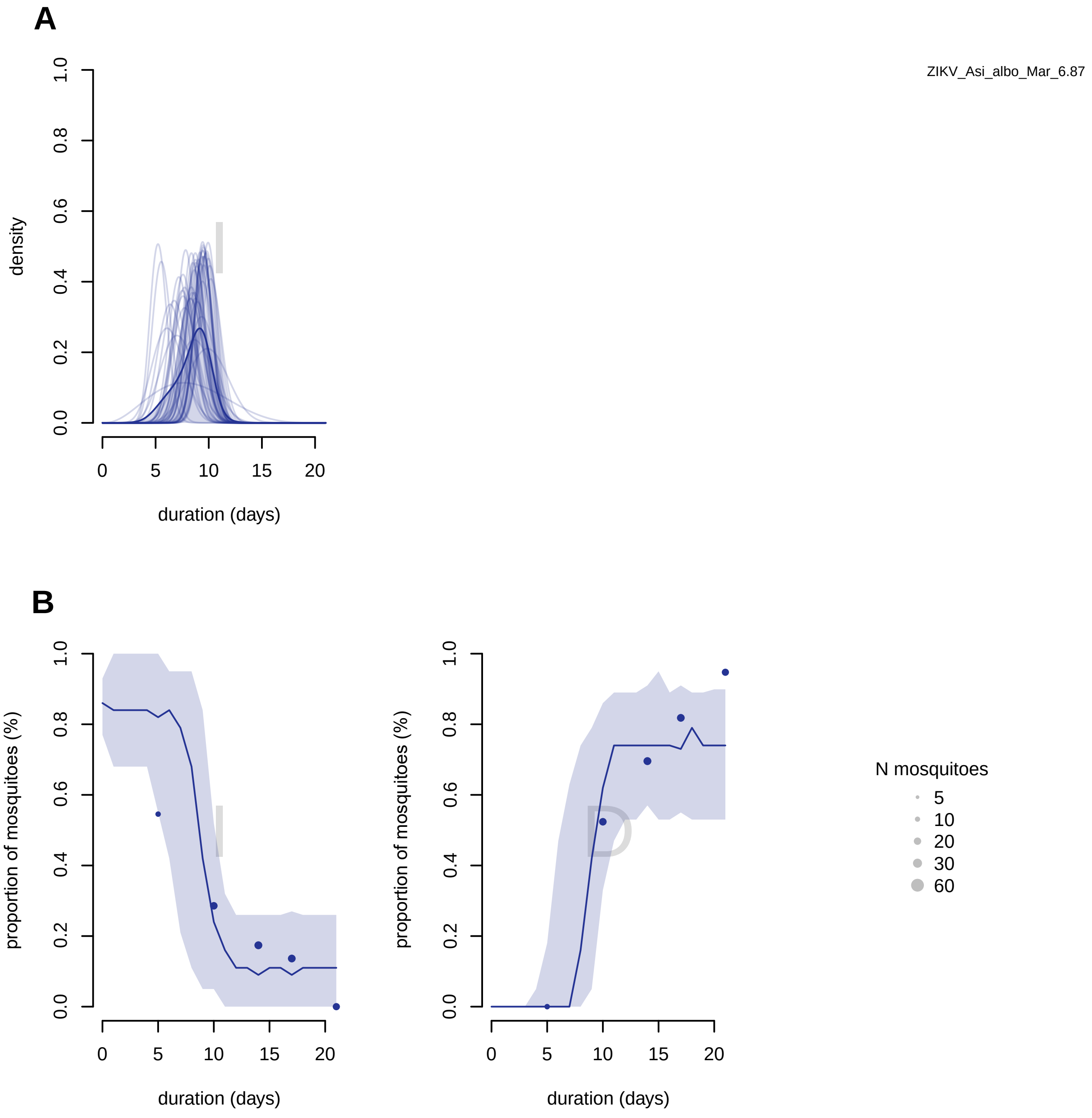

Fig AU. Inference results for IVD stages distributions for scenario ZIKVp2(ZIKV_Asi_albo_Mar_6.87): *Aedes.albopictus* from Marseille infected by Zika virus from Asia with an infectious dose of 6.87 log10 FFU/mL : A) Selected distributions in the infected state for the main model selected. The dark line represents the mean of distributions and light lines represent a random sample of 50 distribution among all selected distribution. B) Selected dynamics in the infected (I) and disseminated (D) states for the main model selected. The dots represent the observed data, the line (mean dynamics), and the uncertainty ribbons (5%-95%) represent selected simulated dynamic.

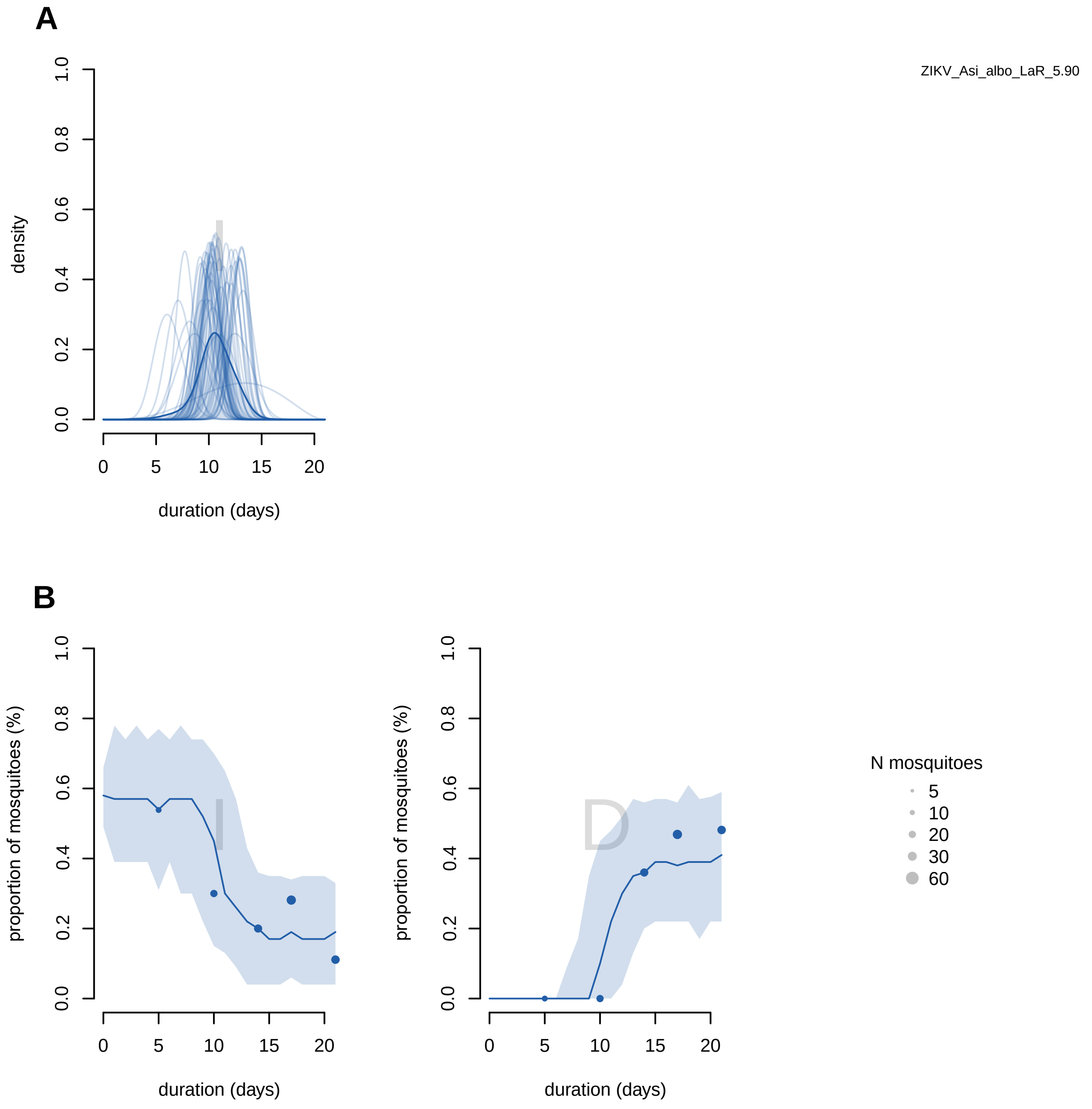

Fig AV. Inference results for IVD stages distributions for scenario ZIKVp3(ZIKV_Asi_albo_LaR_5.90): *Aedes.albopictus* from Reunion Island infected by Zika virus from Asia with an infectious dose of 5.90 log10 FFU/mL : A) Selected distributions in the infected state for the main model selected. The dark line represents the mean of distributions and light lines represent a random sample of 50 distribution among all selected distribution. B) Selected dynamics in the infected (I) and disseminated (D) states for the main model selected. The dots represent the observed data, the line (mean dynamics), and the uncertainty ribbons (5%-95%) represent selected simulated dynamic.

A

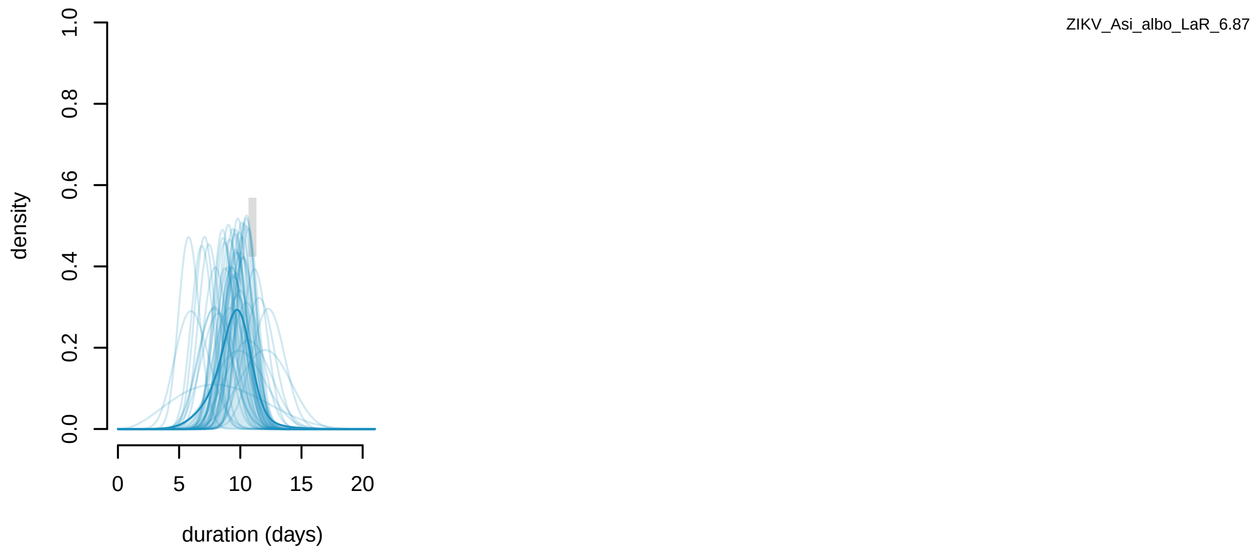

B

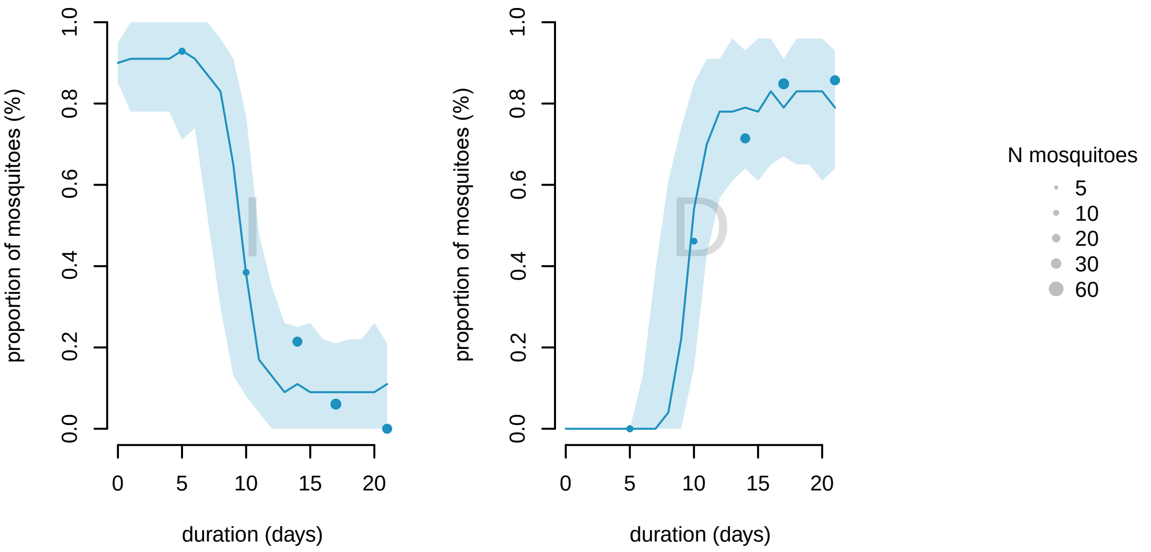

Fig AW. Inference results for IVD stages distributions for scenario ZIKVp4(ZIKV_Asi_albo_LaR_6.87): *Aedes.albopictus* from Reunion Island infected by Zika virus from Asia with an infectious dose of 6.87 log10 FFU/mL : A) Selected distributions in the infected state for the main model selected. The dark line represents the mean of distributions and light lines represent a random sample of 50 distribution among all selected distribution. B) Selected dynamics in the infected (I) and disseminated (D) states for the main model selected. The dots represent the observed data, the line (mean dynamics), and the uncertainty ribbons (5%-95%) represent selected simulated dynamic.

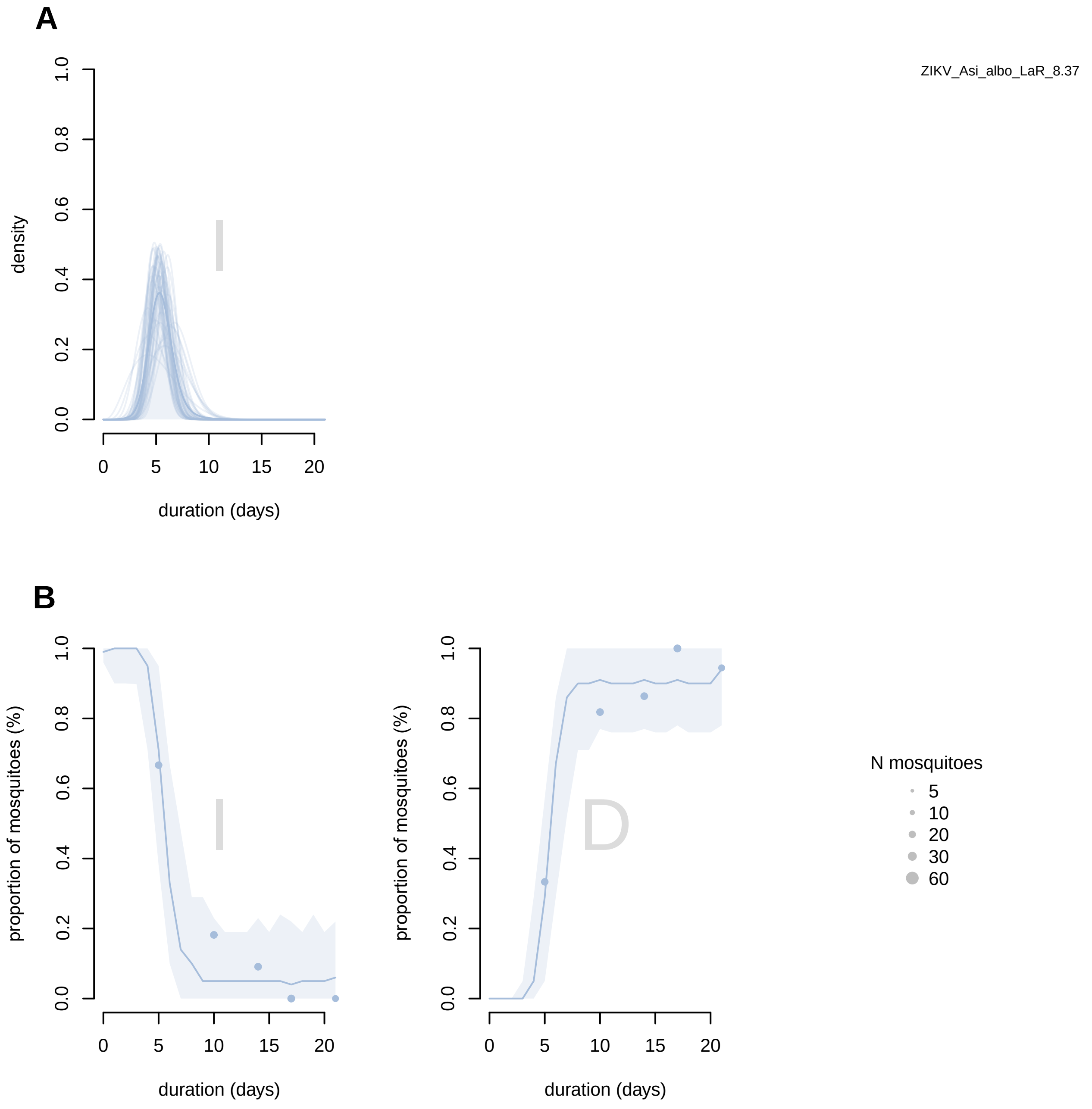

Fig AX. Inference results for IVD stages distributions for scenario ZIKVp5(ZIKV_Asi_albo_LaR_8.37): *Aedes.albopictus* from Reunion Island infected by Zika virus from Asia with an infectious dose of 8.37 log10 FFU/mL : A) Selected distributions in the infected state for the main model selected. The dark line represents the mean of distributions and light lines represent a random sample of 50 distribution among all selected distribution. B) Selected dynamics in the infected (I) and disseminated (D) states for the main model selected. The dots represent the observed data, the line (mean dynamics), and the uncertainty ribbons (5%-95%) represent selected simulated dynamic.

A

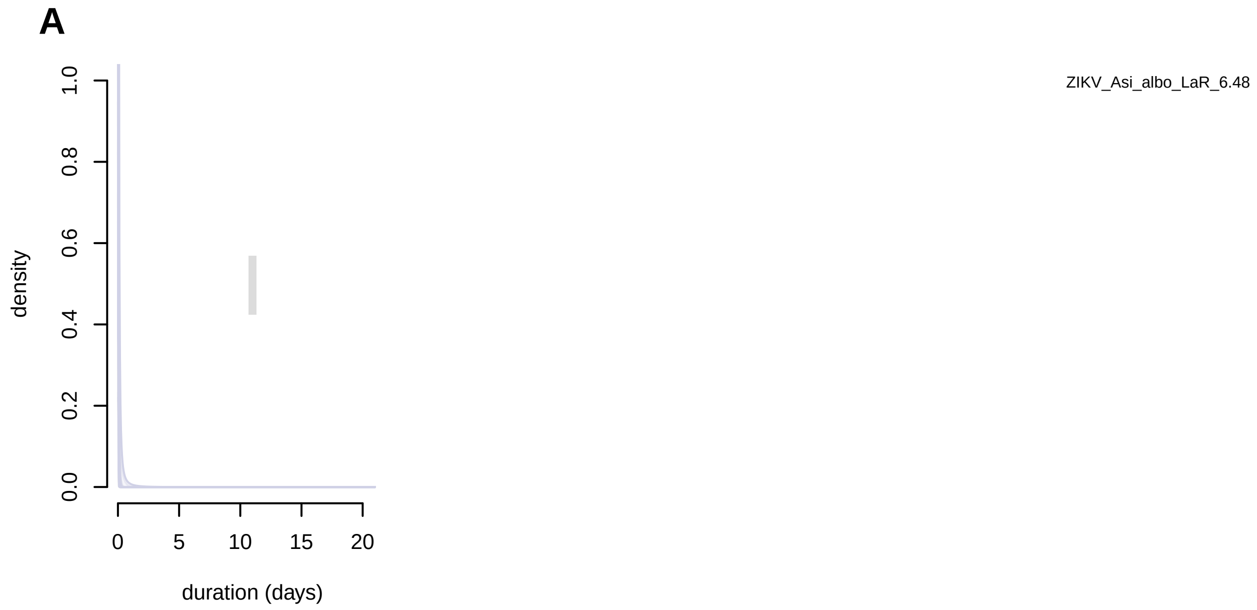

B

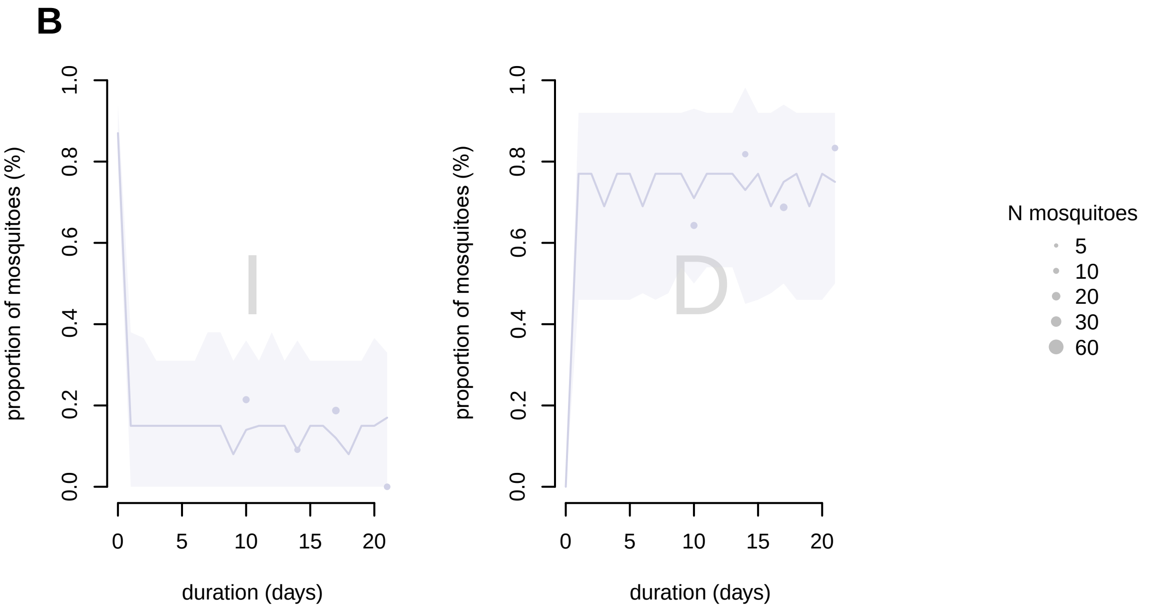

Fig AY. Inference results for IVD stages distributions for scenario ZIKVp6(ZIKV_Asi_albo_LaR_6.48): *Aedes.albopictus* from Reunion Island infected by Zika virus from Asia with an infectious dose of 6.48 log10 FFU/mL : A) Selected distributions in the infected state for the main model selected. The dark line represents the mean of distributions and light lines represent a random sample of 50 distribution among all selected distribution. B) Selected dynamics in the infected (I) and disseminated (D) states for the main model selected. The dots represent the observed data, the line (mean dynamics), and the uncertainty ribbons (5%-95%) represent selected simulated dynamic.
