# Supplementary material for: Intra-vector infection dynamics challenge how to model the extrinsic incubation period for major arboviruses: dengue, Zika, and chikungunya
Source: PLoS Comput Biol. 2025 Aug 25;21(8):e1013393. doi: 10.1371/journal.pcbi.1013393 (PMC12440223; doi:10.1371/journal.pcbi.1013393)
Supplement: S3 Appendix — This appendix contains further tables presenting the statistical analysis of the inference results. (PDF) [file pcbi.1013393.s004.pdf]

S3 Appendix. Statistical analysis of results

This appendix contains further tables presenting the statistical analysis of the inference results.

Table A. Results of inference for each crossing barriers parameters for each scenario with the EIDT model. Scenarios with 90% credibility interval (90% CI) < 0,15 are shown in bold (21/32 for γ^I, 10/32 for γ^D and 4/32 for γ^T).

Scenario	Mode γ_I [90% CI]	Size 90% CI	γ_I from litterature	Mode γ_D [90% CI]	Size 90% CI	γ_D from litterature	Mode γ_T [90% CI]	Size 90% CI	γ_T from litterature
CHIKVc1	0.84 [0.76 , 0.94]	0.18	0.93	0.68 [0.54 , 0.85]	0.32	0.58	0.24 [0.12 , 0.48]	0.36	0.37
CHIKVc2	0.99 [0.97 , 1]	**0.02**	0.99	0.94 [0.88 , 0.99]	**0.11**	0.85	0.25 [0.17 , 0.33]	0.15	0.20
CHIKVc3*	0.98 [0.95 , 1]	**0.04**	0.94	0.82 [0.73 , 0.91]	0.18	0.70	0.62 [0.52 , 0.75]	0.22	0.55
CHIKVc4	0.98 [0.95 , 1]	**0.04**	1.00	0.82 [0.73 , 0.91]	0.18	0.61	0.62 [0.52 , 0.75]	0.22	0.54
CHIKVc5*	0.85 [0.76 , 0.9]	**0.14**	0.83	0.75 [0.65 , 0.83]	0.17	0.76	0.6 [0.51 , 0.7]	0.19	0.61
CHIKVc6*	0.8 [0.74 , 0.86]	**0.12**	0.81	0.92 [0.86 , 0.97]	**0.11**	0.91	0.45 [0.35 , 0.55]	0.20	0.45
CHIKVc7*	0.98 [0.94 , 1]	**0.06**	0.97	0.99 [0.95 , 1]	**0.04**	0.98	0.33 [0.25 , 0.42]	0.17	0.33
CHIKVc8*	0.99 [0.94 , 1]	**0.06**	0.98	0.96 [0.88 , 0.99]	**0.11**	0.94	0.4 [0.32 , 0.49]	0.17	0.40
CHIKVc9*	0.99 [0.96 , 1]	**0.03**	1.00	0.99 [0.94 , 1]	**0.06**	0.98	0.39 [0.3 , 0.5]	0.20	0.40
CHIKVc10*	1 [0.98 , 1]	**0.02**	0.99	1 [0.99 , 1]	**0.01**	1	0.1 [0.06 , 0.13]	**0.08**	0.09
DENVc1	0.53 [0.45 , 0.61]	0.17	0.65	0.78 [0.56 , 0.92]	0.36	0.53	0.21 [0.04 , 0.88]	0.84	0.18
DENVc2	0.23 [0.19 , 0.3]	**0.11**	0.26	0.84 [0.53 , 0.98]	0.45	0.49	0.23 [0.05 , 0.91]	0.86	0.17
DENVc3*	0.18 [0.1 , 0.26]	0.16	0.22	0.73 [0.14 , 0.96]	0.82	0.60	0.73 [0.05 , 0.92]	0.87	0.44
DENVc4	0.92 [0.86 , 0.96]	**0.11**	0.91	0.86 [0.73 , 0.95]	0.22	0.52	0.39 [0.17 , 0.78]	0.61	0.15
DENVc5	0.9 [0.83 , 0.95]	**0.12**	0.92	0.95 [0.77 , 0.99]	0.23	0.61	0.17 [0.03 , 0.87]	0.84	0.11
DENVc6	0.28 [0.21 , 0.39]	0.18	0.30	0.79 [0.52 , 0.96]	0.44	0.48	0.18 [0.04 , 0.61]	0.57	0.30
DENVc7*	0.15 [0.08 , 0.24]	0.15	0.18	0.54 [0.24 , 0.93]	0.69	0.62	0.29 [0.07 , 0.95]	0.87	0.38
ZIKVc1	0.99 [0.98 , 1]	**0.02**	0.99	0.99 [0.95 , 1]	**0.05**	0.96	0.39 [0.32 , 0.49]	0.17	0.29
ZIKVc2	0.9 [0.85 , 0.94]	**0.09**	0.90	0.96 [0.86 , 0.99]	**0.13**	0.75	0.76 [0.56 , 0.94]	0.38	0.44
ZIKVc3	0.45 [0.37 , 0.55]	0.18	0.48	0.66 [0.51 , 0.82]	0.31	0.60	0.74 [0.43 , 0.95]	0.53	0.37
ZIKVc4	0.78 [0.7 , 0.84]	**0.14**	0.83	0.65 [0.49 , 0.78]	0.29	0.47	0.21 [0.03 , 0.9]	0.87	0.12
ZIKVc5	0.9 [0.85 , 0.94]	**0.09**	0.91	0.6 [0.45 , 0.75]	0.31	0.37	0.25 [0.03 , 0.89]	0.86	0.04
ZIKVc6*	0.47 [0.39 , 0.55]	0.17	0.50	0.08 [0.01 , 0.33]	0.32	0.16	0.27 [0.05 , 0.93]	0.88	0.22
ZIKVc7*	0.37 [0.31 , 0.43]	**0.12**	0.38	0.35 [0.21 , 0.63]	0.42	0.27	0.36 [0.05 , 0.92]	0.88	0.25
ZIKVc8*	0.78 [0.71 , 0.87]	0.15	0.82	0.39 [0.22 , 0.63]	0.41	0.25	0.39 [0.03 , 0.94]	0.90	0.05
ZIKVc9*	0.5 [0.42 , 0.59]	0.17	0.53	0.46 [0.34 , 0.71]	0.37	0.45	0.18 [0.03 , 0.44]	0.41	0.23
ZIKVc10*	0.77 [0.71 , 0.84]	**0.14**	0.84	0.63 [0.45 , 0.81]	0.36	0.46	0.36 [0.04 , 0.9]	0.86	0.07
ZIKVc11	0.88 [0.8 , 0.94]	**0.14**	0.9	0.9 [0.83 , 0.95]	**0.12**	0.9	0.7 [0.56 , 0.85]	0.28	0.55
ZIKVc12	0.98 [0.93 , 1]	**0.07**	0.77	0.58 [0.5 , 0.64]	**0.14**	0.4	0.11 [0.05 , 0.18]	**0.13**	0
ZIKVc13*	0.83 [0.76 , 0.88]	**0.13**	0.83	0.52 [0.4 , 0.58]	0.18	0.5	0.06 [0.0 , 0.18]	0.17	0.11
ZIKVc14	0.74 [0.67 , 0.84]	0.17	0.97	0.41 [0.31 , 0.49]	0.19	0.58	0.02 [0 , 0.1]	**0.1**	0.12
ZIKVc15*	0.69 [0.57 , 0.77]	0.20	0.69	0.39 [0.27 , 0.51]	0.24	0.39	0.03 [0.01 , 0.11]	**0.11**	0.03

* scenarios for which the infection, dissemination and transmission rates, taken from articles, were within the 90% CI obtained for the barrier crossing parameters γ_I, γ_D, and γ_T

Table B. Results of inference for each crossing barriers parameters for each scenario with the EID model. Scenarios with 90% credibility interval (90% CI) < 0,15 are shown in bold (14/17 for γ_I and 7/17 for γ_D).

Scenario	Mode γ_I [90% CI]	Size 90% CI	Mode γ_D [90% CI]	Size 90% CI
CHIKVp1	0.02 [0 , 0.04]	**0.04**	0.6 [0.07 , 0.93]	0.86
CHIKVp2	0.72 [0.66 , 0.79]	**0.13**	0.96 [0.86 , 0.99]	**0.13**
CHIKVp3	0.99 [0.95 , 1]	**0.05**	0.99 [0.92 , 1]	**0.07**
DENVp1	0.99 [0.96 , 1]	**0.06**	0.98 [0.9 , 1]	**0.10**
DENVp2	0.98 [0.94 , 1]	**0.07**	0.98 [0.89 , 1]	**0.11**
DENVp3	1 [0.97 , 1]	**0.08**	0.94 [0.83 , 0.99]	0.16
DENVp4	0.99 [0.97 , 1]	**0.09**	0.97 [0.91 , 1]	**0.09**
DENVp5	0.99 [0.96 , 1]	**0.10**	0.89 [0.76 , 0.95]	0.19
DENVp6	0.98 [0.94 , 1]	**0.11**	0.91 [0.8 , 0.98]	0.18
DENVp7	0.99 [0.96 , 1]	**0.12**	0.93 [0.75 , 0.99]	0.23
DENVp8	0.99 [0.97 , 1]	**0.13**	0.74 [0.58 , 0.87]	0.29
ZIKVp1	0.83 [0.77 , 0.88]	**0.14**	0.96 [0.85 , 0.99]	**0.13**
ZIKVp2	0.87 [0.77 , 0.93]	0.15	0.88 [0.77 , 0.95]	0.18
ZIKVp3	0.57 [0.49 , 0.66]	0.17	0.67 [0.56 , 0.8]	0.24
ZIKVp4	0.91 [0.85 , 0.95]	**0.10**	0.89 [0.81 , 0.96]	**0.14**
ZIKVp5	0.99 [0.96 , 1]	**0.04**	0.93 [0.84 , 0.99]	0.15
ZIKVp6	0.86 [0.79 , 0.94]	0.16	0.84 [0.74 , 0.93]	0.19

Table C. Results of the Wilcoxon test used to study the superiority at 0.9 of the γ_I , γ_D and γ_T values (EIDT model). Pvalues < 0,05 corresponding to scenarios for which parameters values are statistically > 0.9 are shown in bold (10/32 for γ_I 9/32 for γ_D and 0/32 for γ_T) .

scenario	γ_I pvalue	γ_D pvalue	γ_T pvalue
CHIKVc1	1	1	1
CHIKVc2	**3.1947121779714e-117**	**1.61599883470573e-94**	1
CHIKVc3	**2.20962136978672e-110**	1	1
CHIKVc4	**1.79395945393212e-105**	1	1
CHIKVc5	1	1	1
CHIKVc6	1	**8.30420846567072e-14**	1
CHIKVc7	**6.96294850737994e-86**	**6.96294850737994e-86**	1
CHIKVc8	**1.95693036570352e-91**	**3.39955867927649e-77**	1
CHIKVc9	**1.4224331828238e-108**	**1.71069404376581e-108**	1
CHIKVc10	**1.12833022090229e-111**	**1.12833022090229e-111**	1
DENVc1	1	1	1
DENVc2	1	1	1
DENVc3	1	1	1
DENVc4	**6.6603154179305e-17**	1	1
DENVc5	0.999968405140845	**0.000121915611642587**	1
DENVc6	1	1	1
DENVc7	1	1	1
ZIKVc1	**1.4734456821593e-132**	**1.4790011082154e-132**	1
ZIKVc2	0.997989130148858	**1.54124620229698e-76**	1
ZIKVc3	1	1	1
ZIKVc4	1	1	1
ZIKVc5	0.602811501831589	1	1
ZIKVc6	1	1	1
ZIKVc7	1	1	1
ZIKVc8	1	1	1
ZIKVc9	1	1	1
ZIKVc10	1	1	1
ZIKVc11	1	0.999029083574895	1
ZIKVc12	1	1	1
ZIKVc13	1	1	1
ZIKVc14	**6.87925622375578e-61**	1	1
ZIKVc15	1	1	1

Table D. Results of the Wilcoxon test used to study the superiority at 0.9 of the γ_I and γ_D values (EID model). P-values < 0,05 corresponding to scenarios for which parameters values are statistically > 0.9 are shown in bold (10/17 for γ_I and 8/17 for γ_D).

scenario	γ_I pvalue	γ_D pvalue
CHIKVp1	1	1
CHIKVp2	1	**1.55002179699219e-60**
CHIKVp3	**6.64172503377505e-93**	**1.44714556069923e-92**
DENVp1	**2.99661165023998e-100**	**6.12994676643568e-93**
DENVp2	**2.99661165023998e-100**	**4.42176195492722e-90**
DENVp3	**2.99661165023998e-100**	**4.57944374121786e-27**
DENVp4	**2.99661165023998e-100**	**3.3115204412706e-96**
DENVp5	**2.99661165023998e-100**	1
DENVp6	**3.04196141838421e-100**	0.900022357291413
DENVp7	**2.99661165023998e-100**	0.998686850092147
DENVp8	**2.7248529270144e-98**	1
ZIKVp1	1	**1.5139955770437e-47**
ZIKVp2	1	1
ZIKVp3	1	1
ZIKVp4	0.519010427774142	0.999999926511964
ZIKVp5	**2.99661165023998e-100**	**1.3348255131e-34**
ZIKVp6	1	1

Table E. Results of the Wilcoxon test used to study the inferiority at 0.5 of the γ_I , γ_D and γ_T values (EIDT model). P-values < 0,05 corresponding to scenarios for which parameters values are statistically < 0.5 are shown in bold (7/32 for γ_I 6/32 for γ_D and 23/32 for γ_T).

scenario	γ_I pvalue	γ_D pvalue	γ_T pvalue
CHIKVc1	1	1	**2.56818261412697e-125**
CHIKVc2	1	1	**3.1947121779714e-117**
CHIKVc4	1	1	1
CHIKVc4	1	1	1
CHIKVc5	1	1	1
CHIKVc6	1	1	**5.05604664244194e-36**
CHIKVc7	1	1	**6.96294850737994e-86**
CHIKVc8	1	1	**2.86977111117595e-89**
CHIKVc9	1	1	**1.0642352774086e-105**
CHIKVc10	1	1	**1.12833022090229e-111**
DENVc1	1	1	**1.39847279239048e-18**
DENVc2	**2.90061772234249e-115**	1	**8.2524889109902e-07**
DENVc3	**2.85400805651275e-83**	0.999999999996661	0.716416349037314
DENVc4	1	1	**2.45691916468642e-19**
DENVc5	1	1	**1.22262900221834e-22**
DENVc6	**8.87668483005163e-75**	1	**5.16473647104948e-57**
DENVc7	**3.92523870829146e-48**	0.99999999999926	0.426393258903374
ZIKVc1	1	1	**1.28122608522439e-130**
ZIKVc2	1	1	1
ZIKVc3	**4.37943933207197e-68**	1	1
ZIKVc4	1	1	**8.09665172169328e-22**
ZIKVc5	1	1	**9.63666290727866e-10**
ZIKVc6	**7.13051578275215e-36**	**1.17446401288081e-104**	0.579774330713689
ZIKVc7	**9.67237173105063e-93**	**1.09701260596836e-51**	**2.35623108497786e-05**
ZIKVc8	1	**2.88279836012155e-51**	**5.00990688705673e-13**
ZIKVc9	0.745710435635756	0.591847563676192	**4.26470649762686e-63**
ZIKVc10	1	1	**2.79949607795003e-11**
ZIKVc11	1	1	1
ZIKVc12	1	**1.11314825803345e-58**	**2.12787185328481e-60**
ZIKVc13	1	**0.0214996959838632**	**2.2240916307875e-61**
ZIKVc14	1	1	**6.87925622375578e-61**
ZIKVc15	1	**1.27805340192947e-72**	**3.00710473305687e-76**

Table F. Visual fit between observation and simulation for EIDT model. Total of scenarios with good to very good quality is equal to 25/32 (= scenarios without dynamics with a fit score qualified of poor quality).

Dpe	Number of Dpe	Mosquito number (mean by Dpe)	Scenario	dynI	dynD	Quality of visual fit between ssObs and ssSim			Scoring visual fit between obs vs sim (/6)
						I	D	T	
3,7,14,21	4	30	CHIKVc1	Beta	Expo	++	+	-	3
3,6,9,12	4	48	CHIKVc2	Beta	Expo	++	+	+	4
3,5,7,10,14	5	41	CHIKVc3	Beta	Expo	++	++	++	6
3,5,7,10,12,14,20	7	19	CHIKVc4	Beta	Expo	-	+	++	3
6,9,14,21	4	39	CHIKVc5	Expo	Expo	-	++	+	3
3,7,10,14,21	5	27	CHIKVc6	Expo	Expo	++	+	+	4
3,5,7,10,12,14,20	7	18	CHIKVc7	Expo	Expo	++	+	+	4
3,6,9,14	4	20	CHIKVc8	Expo	Expo	++	++	++	6
3,6,9,14	4	20	CHIKVc9	Expo	Expo	++	++	++	6
2,4,6,8,10,12,14,16,18,20	10	60	CHIKVc10	Expo	Expo	++	-	-	2
3,7,14,21	4	28	DENVc1	Beta	Beta	+	+	-	2
3,7,10,14,21	5	28	DENVc2	Beta	Beta	+	+	+	3
7,14,21,28	4	17	DENVc3	Beta	Beta	++	++	+	5
2,3,4,5,6,7,10,14	8	21	DENVc4	Beta	Beta	++	+	++	5
2,3,4,5,6,7,10,14	8	21	DENVc5	Beta	Beta	+	++	-	3
7,14,21,28	4	17	DENVc6	Beta	Expo	++	++	++	6
7,14,21,28	4	18	DENVc7	Beta	Beta	++	++	+	5
2,4,6,8,10,12,14,16,18,20	10	30	ZIKVc1	Beta	Beta	+	++	+	4
6,9,14,21	4	39	ZIKVc2	Beta	Beta	++	++	+	5
5,7,10,14	4	26	ZIKVc3	Beta	Beta	++	++	++	6
6,9,14,21	4	32	ZIKVc4	Beta	Beta	+	++	++	5
6,9,14,21	4	38	ZIKVc5	Beta	Beta	+	+	+	3
3,7,14,21	4	30	ZIKVc6	Beta	Beta	+	+	++	4
6,9,14,21	4	39	ZIKVc7	Beta	Beta	++	++	+	5
6,9,14,21	4	26	ZIKVc8	Beta	Beta	++	++	+	5
7,10,14,21	4	23	ZIKVc9	Beta	Expo	++	++	++	6
6,9,14,21	4	29	ZIKVc10	Beta	Beta	+	++	++	5
6,9,14,21	4	28	ZIKVc11	Expo	Beta	++	++	++	6
6,9,14,21	4	30	ZIKVc12	Expo	Expo	+	+	++	4
6,9,14,21	4	34	ZIKVc13	Expo	Expo	+	+	++	4
6,9,14,21	4	30	ZIKVc14	Expo	Expo	++	++	++	6
6,9,14,21	4	30	ZIKVc15	Expo	Expo	-	+	++	3

(-): poor quality (at least two points outside the envelope/high dispersion of distributions around the mean),
(+): good quality (one point outside the envelope/slight dispersion of distributions around the mean),
(++): very good quality (no point outside the envelope, distributions strongly clustered around the mean).

Table G. Visual fit between observation and simulation for EID model. Total of scenarios with good to very good quality is equal to 17/17 (= scenarios without dynamics with a fit score qualified of poor quality).

Dpe	Mosquito number (mean by Dpe)	Scenario	dynI	Quality of visual fit between ssObs and ssSim		Scoring visual fit between obs vs sim (/4)
				I	D	
2,6,9,14	43	CHIKVp1	Beta	++	++	4
	31	CHIKVp2	Beta	++	++	4
	19	CHIKVp3	Expo	++	++	4
4,6,8,12,18	16	DENVp1	Beta	++	++	4
	21	DENVp2	Beta	++	++	4
	17	DENVp3	Beta	++	++	4
	25	DENVp4	Beta	++	++	4
	17	DENVp5	Beta	++	+	3
	15	DENVp6	Beta	++	++	4
	19	DENVp7	Beta	++	+	3
	19	DENVp8	Beta	++	++	4
5,10,14,17,21	26	ZIKVp1	Beta	++	+	3
	19	ZIKVp2	Beta	++	+	3
	23	ZIKVp3	Beta	++	++	4
	23	ZIKVp4	Beta	++	++	4
	21	ZIKVp5	Beta	++	++	4
	13	ZIKVp6	Expo	++	++	4

(-): poor quality (at least two points outside the envelope/high dispersion of distributions around the mean),
(+): good quality (one point outside the envelope/slight dispersion of distributions around the mean),
(++): very good quality (no point outside the envelope, distributions strongly clustered around the mean).

Table H. Mean of RMSE (root mean squared error) for selected dynamics in each states for EIDT model. Total of scenarios with a mean RMSE lower than 5 is equal to 21/32, 23/32, and 26/32 for the infected, disseminated, and transmitter states, respectively.

scenario	mean rmse I	mean rmse D	mean rmse T
CHIKVc1	5	5	5
CHIKVc2	3	6	6
CHIKVc3	5	4	5
CHIKVc4	3	3	3
CHIKVc5	6	4	6
CHIKVc6	2	4	5
CHIKVc7	1	3	3
CHIKVc8	2	3	3
CHIKVc9	1	3	4
CHIKVc10	1	7	7
DENVc1	6	4	2
DENVc2	3	2	1
DENVc3	2	1	1
DENc4	2	3	2
DENVc5	4	4	2
DENVc6	2	2	1
DENVc7	1	1	1
ZIKVc1	2	3	3
ZIKVc2	4	5	4
ZIKVc3	3	3	2
ZIKVc4	6	5	2
ZIKVc5	5	5	1
ZIKVc6	7	2	1
ZIKVc7	4	2	1
ZIKVc8	4	4	1
ZIKVc9	3	2	1
ZIKVc10	6	5	1
ZIKVc11	3	4	4
ZIKVc12	5	4	1
ZIKVc13	5	6	1
ZIKVc14	3	3	2
ZIKVc15	6	5	1

Table I. Mean of RMSE for selected dynamics in each states for EID model.

scenario	mean rmse I	mean rmse D
CHIKVp1	1	1
CHIKVp2	2	4
CHIKVp3	1	2
DENVp1	1	1
DENVp2	2	2
DENVp3	2	2
DENVp4	2	2
DENVp5	2	2
DENVp6	2	2
DENVp7	2	2
DENVp8	3	3
ZIKVp1	2	3
ZIKVp2	3	3
ZIKVp3	3	3
ZIKVp4	3	3
ZIKVp5	2	2
ZIKVp6	2	2

Table J. Analysis of the observed dynamics in each state for each scenario with complete data (ie: data with infected, disseminated and transmitter states observed).

	Dynamics in state I					Dynamics in state D					Dynamics in state T			
scenario	↘	↗↘	↘↗	↗↘↗	→	↗	↗↘	↗↘↗	↘↗	↘↗↘	↗	↘	↗↘	→
CHIKVc1	1					1							1	
CHIKVc2	1					1							1	
CHIKVc3	1								1				1	
CHIKVc4	1							1			1			
CHIKVc5	1						1				1			
CHIKVc6	1								1				1	
CHIKVc7	1								1				1	
CHIKVc8	1								1				1	
CHIKVc9	1								1				1	
CHIKVc10					1	1						1		
DENVc1		1				1					1			
DENVc2		1				1					1			
DENVc3			1				1				1			
DENVc4	1						1				1			
DENVc5	1						1				1			
DENVc6			1				1						1	
DENVc7	1						1						1	
ZIKVc1	1							1					1	
ZIKVc2	1						1				1			
ZIKVc3	1						1				1			
ZIKVc4		1				1					1			
ZIKVc5		1							1					1
ZIKVc6		1					1				1			
ZIKVc7	1					1					1			
ZIKVc8	1					1								1
ZIKVc9	1						1				1			
ZIKVc10				1						1	1			
ZIKVc11			1				1						1	
ZIKVc12		1							1					1
ZIKVc13		1							1					1
ZIKVc14	1						1				1			
ZIKVc15		1					1							1

Table K. Results of the Kolmogorov-Smirnov test: percentage of similar beta distributions in the infected state (I) for each scenario with a beta distribution mainly selected in I. Scenarios for which ≥50% of p-value were > 0.05, corresponding to the percentage of distribution which are statistically similar, are shown in bold (13/21 for the EIDT model and 11/15 for the EID model).

Scenarios EIDT model	Percentage p-value >0.05	Scenarios EID model	Percentage p-value >0.05
CHIKVc1	33	**CHIKVp1**	**50**
**CHIKVc2**	**61**	CHIKVp2	41
CHIKVc3	39	**DENVp1**	**82**
**CHIKVc4**	**61**	**DENVp2**	**69**
**DENVc1**	**57**	**DENVp3**	**79**
DENVc2	45	**DENVp4**	**87**
**DENVc3**	**56**	**DENVp5**	**62**
**DENVc4**	**62**	DENVp6	42
DENVc5	46	**DENVp7**	**59**
**DENVc6**	**61**	DENVp8	27
**DENVc7**	**55**	**ZIKVp1**	**51**
ZIKVc1	44	**ZIKVp2**	**56**
**ZIKVc2**	**54**	**ZIKVp3**	**64**
**ZIKVc3**	**54**	ZIKVp4	47
ZIKVc4	45	**ZIKVp5**	**50**
**ZIKVc5**	**57**		
ZIKVc6	49		
**ZIKVc7**	**51**		
ZIKVc8	47		
**ZIKVc9**	**61**		
**ZIKVc10**	**62**		

Table L. Results of the Kolmogorov-Smirnov test: percentage of similar beta distributions in the disseminated state (D) for each scenario with a beta distribution mainly selected in D. Scenarios for which ≥50% of p-value were > 0.05, corresponding to the percentage of distribution which are statistically similar, are shown in bold (7/16).

Scenario	Percentage p-value >0.05
DENVc1	47
**DENVc2**	**50**
DENVc3	46
**DENVc4**	**53**
DENVc5	45
**DENVc7**	**50**
**ZIKVc1**	**54**
**ZIKVc2**	**50**
**ZIKVc3**	**52**
ZIKVc4	49
ZIKVc5	48
ZIKVc6	46
ZIKVc7	48
ZIKVc8	49
**ZIKVc10**	**53**
ZIKVc11	49
