# Supplementary material for: Intra-vector infection dynamics challenge how to model the extrinsic incubation period for major arboviruses: dengue, Zika, and chikungunya
Source: PLoS Comput Biol. 2025 Aug 25;21(8):e1013393. doi: 10.1371/journal.pcbi.1013393 (PMC12440223; doi:10.1371/journal.pcbi.1013393)
Supplement: Attachment — Submitted filename: PLOS COMP BIology Review - 16 December 2024.pdf [file pcbi.1013393.s006.pdf]

The manuscript submitted by Loisel et al describes a compartmental model to better predict the probability that exposed mosquitoes become infectious during the extrinsic incubation period for a variety of arboviruses. The authors use data found in existing literature to evaluate the ability of their compartmental model to predict experimentally-derived infection probabilities. While the model presented fits the experimental data well and provides a useful framework for modeling the probability of infectious mosquitoes for better understanding transmission, there is some confusion in how the proposed model fits in with existing frameworks used to model vector-borne disease transmission. For example, in most equations of vector-borne disease transmission such as R0 or vectorial capacity, in addition to EIP there is usually a vector competence variable to describe the probability of an exposed mosquito to become infectious. The authors don't seem to emphasize how this has been done already in the introduction and discussion and make it seem like EIP is used independently of vector competence in many transmission models when this is not the case. I think the manuscript needs to be revised to better acknowledge how the authors' newly presented model with three compartments in IVD compares to typical presentations of a single parameter for vector competence in the equation for R0 and vectorial capacity instead of only comparing to one exponential approach vs the author's approach. Does the work presented here allow researchers to better estimate the vector competence variable already used in most transmission models? Or is it independently used to estimate vector competence for a different type of transmission model? Some clarity on how this new framework fits in with existing frameworks rather than just a single variable within other frameworks (EIP) is needed. For example R0 for vector-borne diseases typically is described as $(N(ah)^2bcp^n)/H(-ln(p))r$ where N represents vector abundance, a the daily biting rate of the vector, h the proportion of bites by vectors on susceptible hosts, b represents the per bite probability of a vector becoming infected given exposure, c is the per bite probability of a host becoming infected given exposure, p is the vector daily survival rate, n is the extrinsic incubation period of the parasite in days, H is the abundance of susceptible hosts in the population, and r is the daily recovery rate of infected hosts. I understand that EIP could be a distribution and assumes that all mosquitoes have the same EIP but variation in mosquitoes becoming infectious is already accounted for elsewhere in the R0 equation by the variables p and b. It would be helpful if the authors could explain why the three compartments of IVD are more useful to have than a single compartment currently in use in most models. As the manuscript currently reads, there is more focus on the EIP which is a separate variable from vector competence and should be more clearly explained. Below, I've made some suggestions line by line for where I think additional improvements in this manuscript could be made. Overall, I think it is well written and the model is presented as a useful tool for better predicting vector transmission dynamics.

Major edits:

- Lines 86 -87: There are examples where vector competence is not represented as the inverse of EIP, discuss those examples and how those examples perform compared to the framework you present here.
- Lines 101 - 112: I think it might be useful to show the big picture transmission model most researchers use to represent transmission and which part of that model the work

you present here impacts. For example, the authors call out the EIP parameter but I think the work here more directly predicts the vector competence parameter. Provide an explanation for how these variables relate and which part(s) of transmission models the authors are improving in this study (i.e. estimates of vector competence by using a 3 compartment model). Is it the EIP parameter or the vector competence parameter and how it varies across EIP?

- Lines 260-1: “Although the existence of these barriers is already known (33), the majority of epidemiological models of vector-borne disease transmission does not take them into account and assumes that all exposed mosquitoes eventually become infectious” – in most models of R0 or vectorial capacity for vector-borne diseases, there is a vector competence term that is not assumed to be 1. For example, see Smith DL, McKenzie FE, Snow RW, Hay SI (2007) Revisiting the Basic Reproductive Number for Malaria and Its Implications for Malaria Control. PLOS Biology 5(3): e42. https://doi.org/10.1371/journal.pbio.0050042, where c is used to describe the probability that a mosquito becomes infected given a bite on an infectious human. There are many papers in the malaria context where transmission models contain parameters to describe the probability that a mosquito becomes infected and most assume that this probability is not 1 and varies due to the barriers already mentioned in your manuscript. There is also substantial literature which already acknowledges variation in transmission probabilities due to variability in the barriers or things that impact these barriers, for example see Lefevre T, Ohm J, Dabiré KR, et al. Transmission traits of malaria parasites within the mosquito: Genetic variation, phenotypic plasticity, and consequences for control. Evol Appl. 2018; 11: 456–469. https://doi.org/10.1111/eva.12571. Please acknowledge the approach others have taken to incorporate variation in vector competence in transmission models here and compare your method to methods previously used. In Aedes, there are equally plentiful examples of prior work which acknowledges variation in vector competence which is accounted for in the vectorial capacity equation, for example: Effect of Larval Competition on Extrinsic Incubation Period and Vectorial Capacity of Aedes albopictus for Dengue Virus Bara J, Rapti Z, Cáceres CE, Muturi EJ (2015) Effect of Larval Competition on Extrinsic Incubation Period and Vectorial Capacity of Aedes albopictus for Dengue Virus. PLOS ONE 10(5): e0126703. https://doi.org/10.1371/journal.pone.0126703.
- Line 279: “considerable additional amount of data would be required” – what data is required? Provide recommendations for what type of data need to be experimentally collected and why to help readers understand the what and why of this statement. You mention some of these things later in the discussion, but would be good to list earlier.
- Lines 281 - 284: Could the authors provide specific examples of the types of data and experiments that are currently lacking but would be informative to the field? Similarly, what would the proposed standardized data format be? What sorts of standards would the authors recommend?
- Lines 330 - 334: It would be very useful to experimental biologists to be given more clear recommendations on things like sample sizes for experiments – I wonder if the authors could provide examples of experimental designs they would recommend for different

types of experiments based on the model results and their experience using the existing data in the literature to support this recommendation
- Lines 402 - 416: why is it better to compartmentalize IVD into 3 stages instead of just having one transmission probability variable?

Minor edits:

- Abstract: Rephrase “To elucidate transmission, anticipate their spread and efficiently control them, mechanistic modelling has proven its usefulness.” for clarity. Suggestion: “Mechanistic modeling has proven useful to elucidate transmission, anticipate the spread, and predict the response of arboviruses to control tools.”
- Abstract: Missing “on” in “most models rely assumptions” in cover page (but appears in abstract of main doc)
- Abstract, line 23: change “two strong hypotheses” to “two strong assumptions”?
- Abstract, line 25: change “To assess these hypotheses” to “To assess the validity of these assumptions”
- Line 39: change “poses” to “posed”
- Line 42: specify for arboviruses instead of just general epi models?
- Line 90: change “every exposed mosquitoes” to “every exposed mosquito”
- Line 91: change “is better represented by non-exponential distribution to “is better represented by a non-exponential distribution”
- Line 105: change “infection” to “infections”
- Figure 1: there are gray bars in A-C, ii and iii around x=day10, y=0.5. What are these designating?
- Line 147: Figure 1 caption missing (i) description. Why do “modBetaBeta, modBetaExpo, modExpoBeta, modExpoExpo” listed in caption not match models listed in top legend (“IbetaDexpo, IbetaDbeta, IexpoDexpo, IexpoDbeta”)?
- Line 154: change “exposition” to “exposure”
- Line 232: change “demonstrating” to “demonstrated”
- Line 245: change diseases to disease
- Line 256: mention what is meant by “others”. Suggest “other vector-borne diseases like malaria”?
- Line 262: changes “allows to” to “allows us to”
- Line 313: change “defining” to “define”
- Line 359 - 361: wording is unclear, please rephrase for clarity
- Line 394: change “This could enable to study” to “This could enable researchers to study”
- Figure S2.2: Change “on body”, “on saliva” to “in body”, “in saliva”
- -Figure 1: “effectif” is in French? Translate to English
-
