# Supplementary material for: Intra-vector infection dynamics challenge how to model the extrinsic incubation period for major arboviruses: dengue, Zika, and chikungunya
Source: PLoS Comput Biol. 2025 Aug 25;21(8):e1013393. doi: 10.1371/journal.pcbi.1013393 (PMC12440223; doi:10.1371/journal.pcbi.1013393)
Supplement: Attachment — Submitted filename: response_to_reviewers.pdf [file pcbi.1013393.s009.pdf]

ANSWERS TO REVIEWERS

Loisel et al., submitted in PLOS CB

PCOMPBIOL-D-24-01822

Intra-vector infection dynamics challenges how to model extrinsic incubation period for major arboviruses: dengue, Zika, and chikungunya

We would like to thank the three reviewers for their interest in our work and their useful and accurate comments that greatly helped us improving our manuscript.

We explain below how we addressed their remarks and what was changed in the text to take them into account.

We also would like to reinforce as an introduction to our response letter the novelty of our work. First, our study estimates simultaneously the proportion of mosquitoes for which viruses do not cross each of the three barriers and the distribution of the duration in each of the two stages (infected and disseminated) between viral exposure and transmission. The findings of this study demonstrate that, in contrast to the common assumption, these distributions are rarely exponential, especially for DENV and ZIKV. Furthermore, duration distributions and barriers parameters are usually aggregated (single barrier and single duration) and estimated independently. As a result, it is impossible to assess if they are correlated, which thus is never accounted for in transmission models. In contrast, our framework provides parameter estimates that are altogether coherent. Instead of using knowledge from different publications (on transmission rate and EIP), our framework provides a consistent set of parameters for intra-mosquito barriers and duration distributions during EIP. Such correlations could be statistically quantified using our framework upon additional vector competence data. Furthermore, our study accounts for all (as far as we found) of the published vector competence experiments with comparable protocols (15 articles for an overall 43 experimental data sets), allowing to conclude about intra-vector infection dynamics (IVD) parameters for the three major viruses studied. This work provides a bridge between epidemiology and medical entomology by showing the added value of estimating IVD parameters based on experimental vector competence data to better understand vector-borne disease transmission.

We made this clearer in the article, in introduction (lines 105-126), results (lines 138-142), and discussion (lines 270-310).

Reviewer #1:

R1.1: In this manuscript, the authors present a stochastic model of the progress of mosquitoes through stages of viral infection, from exposure to the ability to transmit. While the authors present this model as 'within-vector viral dynamics', this is not actually what is modelled. There are no virus dynamics – changes in viral population over time - in the model, rather they simulate the progression of a population, or sample of mosquitoes, through infection categories.

First, we would like to thank reviewer #1 for his comments and help in improving our paper.

We agree that our model does not represent or quantify viral populations over time. Our aim is to propose a qualitative mechanistic model of intra-vector infection dynamics that can be integrated into broader vector-host transmission models. Rather than modelling changes in viral populations, our approach simulates the progression of a mosquito through its different infection stages (infected, disseminated, infectious). Focusing on mosquito stages allows us to use a large panel of qualitative vectorial competence data rather than intra-mosquito quantitative data on virus load which are scarce. We believe our model can be categorized as a "intra-vector infection model" because it addresses the progression of mosquito throughout infection stages which directly rely to intra-mosquito viral dynamics. We have clarified the qualitative nature of our model in the introduction (lines 105-106) to ensure this distinction is clearer to readers. In addition, we have renamed our model 'intra-vector infection dynamics model' instead of 'intra-vector viral dynamics model' in order to remove the word 'viral', which can be misleading about the type of model built.

R1.2: There are three main insights from their work, derived from calibrating the model to published competence data: i) that the distribution of the EIP for DENV and ZIKV is not exponential; ii) that intra-mosquito barriers are not systematically crossed; and that intra-vector viral dynamics are influenced by biotic factors. I would highlight that none of these conclusions are particularly novel (e.g. see https://journals.plos.org/plosone/article?id=10.1371/journal.pone.0050972, https://pmc.ncbi.nlm.nih.gov/articles/PMC10771300/, http://www.sciencedirect.com/science/article/pii/S1567134818307159).

In addition to confirm that the intra-mosquito barriers were not systematically crossed, our model allows them to be represented and calibrated in conjunction with the duration of the two associated IVD stages, which is fully novel, as now more clearly stated in introduction (lines 105-110 and lines 118-123) and further discussed (lines 291-300).
The first article listed above was cited in the discussion line 284, where its differences compared to our study were mentioned. While this article provides insightful knowledge on EIP distribution for DENV with a meta-analysis, its authors aggregated information from multiple experiments with different infectious doses, virus serotypes and mosquito species. In contrast, we aimed to proposed a framework which permits to establish the direct link between one set of experimental conditions (one mosquito and one virus genotypes, at one infectious dose) and the distribution of the duration in IVD stages without aggregating information from multiple experiments. Our framework is easy to handle and can be used for any other arboviruses. Once enough experimental data will be published, and associated IVD parameters estimated, statistical associations could be derived between parameters' values and experimental conditions and (a)biotic factors, thus offering a way to better interconnect within-vector viral dynamics and epidemiological situations. The section on the impact of biotic factors on the intra-vector infection dynamics has been removed from the results and incorporated into the discussion as advised in major comments (see below point R1.4).

Major comments

R1.3: We would recommend that the manuscript, particularly how the modelling and results are described, be re-written so that it is much clearer to understand. We provide some specific examples of this in the minor comments to try to help with this. We also suggest that the authors think about how best to present the work in figures as these are also difficult to interpret.
We have revised the manuscript to improve the clarity of the model and result descriptions, following your suggestions. We have also enhanced the readability and interpretability of the figures based on your feedbacks. (see details in "minor comment" part, R1.12-R1.25 for results and R1.26 –R1.30 for model description)

R1.4: It is clear from your third section (L185) that all of your parameter estimates, and selected distributions are conditional on individual experiment settings. While this is acknowledged in this section as a reason why estimates varied, I would argue that this is not a result *per se* and indeed is certainly not a novel result, but a limitation to your study in that you don't account for any of these variations. Importantly, the one to note which stands as separate is the virus dose/ concentration of virus in the bloodmeal as this directly influences the viral dynamics as it is the amount of starting virus – this is probably a major driver of variation in your parameter estimates. I would suggest this is all really for the discussion.

This section has been deleted from the results and this point is now mentioned only in the discussion.

Lines 312-319 : "Besides influencing the EIP duration (10,14), biotic factors (mosquito and virus genotypes, and viral infectious dose) also impact the distributions of residence time in IVD stages, as well as the probabilities of crossing intra-mosquito barriers (10). However, for establishing a quantitative statistical link between such biotic factors and IVD parameters, much more experimental data are needed, which currently are not available in the literature. Nevertheless, the variety of experimental data already available - and used in our study - enabled us to propose a modelling framework easy to use on novel data once available. This framework leads to estimate consolidated parameter sets according to such biotic factors and usable in epidemiological models to better assess outbreak management measures."

R1.5: The last section from L194 could be made more succinct and more clearly explained. The main statement is true for any modelling that uses data, and the explanation in this section is quite dense so it is hard to see the useful results. The language is also sometimes vague. We've tried to articulate how this is with specific examples in the minor comments.

We have revised this section to improve its clarity following your suggestions (see "minor comments" part, R1.21-25).

It would have been good if the authors had gone the step further and shown in this piece of work the impacts of their findings for transmission by including their fitted distributions and parameter estimates in a transmission model. This may make the publication move a step further from findings that are already generally well accepted.

We agree that incorporating fitted distributions and parameter estimates into a transmission model is the natural next step of our work. However, we do not agree that such findings are well accepted in the modelling community. Indeed, among recently published vector-borne disease models, almost none makes the assumption of a non-exponential EIP duration, without mentioning this assumption, thus making it highly implicit (https://www.ncbi.nlm.nih.gov/pmc/articles/PMC8845267/, https://www.ncbi.nlm.nih.gov/pmc/articles/PMC5866925/). Our work challenges this hypothesis for three arboviruses and proposes a means of studying the actual distribution of IVD durations for other arboviruses. On the other hand, as stated in our introduction, it has been shown for dengue that varying the distribution of the within-vector EIP and within-host incubation duration impacts peak dates and amplitude of host epidemic dynamics, but without disconnecting the two processes. Hence, more knowledge is needed to quantify the impact of using a more realistic EIP distribution. However, this would require a huge amount of work and is beyond the scope of this article. Our intra-vector model can be seen as a founding framework that hopefully will stimulate future experimental and modelling research.

Minor comments

Introduction

R1.6: Do movements of peoples and goods with environmental changes necessarily imply global expansion? Suggest here that you clarify you mean expansion of the distribution of arboviral diseases/ increased geographic range, as 'global expansion' is ambiguous.

The suggestion has been taken into account and added to the text.

Lines 54-56: “This expansion in the worldwide distribution of arboviruses, combined with an increasing resistance of mosquitoes to insecticides and the lack of vaccines (6), complicates the control of these diseases (3,4).”

R1.7: please elaborate on what you mean by ‘robust provisions’ and how better understanding transmission dynamics can contribute to limiting future threats by giving tangible examples in the introduction, or omit this statement. It’s easy to make these statements, less easy to justify.

The suggestion has been taken into account and added to the text.

Lines 60-62: “Improving our knowledge of their transmission dynamics is one of the objectives proposed by the World Health Organization to anticipate and limit future epidemics in its ‘Global Initiative on Arboviruses’ (9)”.

R1.8: ‘*high accuracy and reliability of these models are crucial to guide arbovirus surveillance and implement efficient disease management*’ – the reference given with this statement is https://pubmed.ncbi.nlm.nih.gov/24245625/ . I would argue that this reference does not provide evidence that accuracy and reliability of transmission models are crucial to guide arbovirus surveillance and control. The study covers a retrospective modelling analysis of an outbreak. I would suggest that arbovirus surveillance and control can be implemented quite well without any transmission modelling – see - what is crucial is good data and financing. In this paper, they do refer to the use of statistics. Perhaps you can just remove this statement.

Using a model calibrated on epidemiological data, this article demonstrated the effect of intervention starting date on dengue final epidemic size. It thus illustrates how epidemic models could be useful to better select when to start a costly or difficult-to-accept intervention. We have modified the sentence to temper our statement:

Lines 67-69 : "The accuracy and reliability of these models are essential to better guide and assist arbovirus surveillance and help implementing effective disease management interventions (12)."

R1.9: ‘*most transmission models do not explicitly represent IVD but provides an approximation with a single compartment whose exit rate is assumed constant and equal to the inverse of the EIP*’. While this may be true, it should be highlighted/ acknowledged here that it is probably chosen as a simplifying assumption by modellers because varying the latent period distribution is assumed not to importantly impact the answer to the question they are using the model for.

Among recently published vector-borne disease models, almost none makes the assumption of a non-exponential EIP duration, without discussing the impact it could have on their previsions, making this assumption highly implicit. As the impact of EIP distribution on epidemic dynamics has not been yet quantified, we would rather recommend that scientists systematically think about this hiding assumption and make sure its possible impact can be neglected, or at least discuss it.

R1.10: ‘*the successful crossing of all three barriers by viruses in every exposed mosquitoes’* – this is not a common assumption in vector-borne disease models – usually this is accounted for by including a parameter for the host-to-mosquito transmission probability and the mosquito-to-host transmission probability.

We concur with your assertion. We have removed this statement from our manuscript. Indeed, the benefit of our model is that it estimates simultaneously the proportions of mosquitoes in which viruses do not cross each of the three barriers and the distribution of the duration in each of the two stages between viral exposure and transmission. Usually, these parameters are aggregated (single barrier and single duration) and estimated independently. As a result, it is impossible to assess if they are correlated, which is never accounted for in transmission models. In contrast, our framework provides parameter estimates that are altogether coherent. Instead of using knowledge from distinct publications on transmission rate and EIP, our framework provides a consistent set of parameters. When more virology data become available, such correlations could be statistically quantified using our framework. We modified explanations in the text in this way in introduction (lines 105-110 and lines 118-123) and in discussion (lines 291-300).

R1.11: L107: You refer to three models of within-mosquito viral dynamics, outlining a gap in the research that there is no full model through to salivary gland infection, but in fact your model does not fill this gap as it is not a model of within-mosquito viral dynamics – you do not explicitly model viral dynamics within individual mosquitoes. I think you just need to re-phrase how you describe your modelling here.

The suggestion has been taken into account and the text has been modified.

Lines 101-110: "Recent studies have investigated the role of infectious dose in two of the three stages of IVD—either infection and dissemination or infection and transmission for DENV (26) and ZIKV (27,28). For DENV, viral titre has been explicitly represented, while for ZIKV, both viral titre and the number of infected cells have been modelled. These contributions have significantly advanced our understanding of the biotic factors affecting the crossing of barriers within the mosquito. A generic mechanistic model of mosquito states during IVD is however still lacking. Such a model would enable integrating a wider range of experimental conditions and simultaneously estimating both the proportion of mosquitoes in which viruses fail to cross each barrier and the distribution of IVD stages. Typically, these parameters are estimated independently, making it impossible to account for their potential correlations in transmission models."

Results

R1.12: It would be useful to present the table of the fitted model details in the main manuscript. In the figure legends, please provide descriptions that stand alone without having to refer to other parts of the manuscript, so that someone can look at the figures and understand them without too much effort.

A conceptual diagram of the IVD model and a table of model parameters have been added in the first paragraph of the results, and the captions of the figures have been completed to make them easier to understand. (Lines 143-164, Fig.1 and Table 1)

R1.13: What do you mean by 'best' – can you state more explicitly, especially given the methods are presented at the end, how the distribution was selected.

The suggestion has been taken into account and added to the text.

Lines 134-138 : "Using vector competence data available in the literature for DENV, ZIKV, and CHIKV, we inferred model parameter values within our IVD framework (Fig.1 and Table 1) using an Approximate Bayesian Computation - Sequential Monte Carlo (ABC-SMC) approach combined with model selection. Specifically, we compared models differing by the use of either a beta or an exponential distribution for the durations in the infected and disseminated states."

R1.14: Rather than 'much more frequently' can you just state quantitatively instead?

The suggestion has been taken into account and added to the text.

Lines 165-167: "Indeed we demonstrated that the beta distribution was selected 1.7 times more often than the exponential distribution. This was particularly evident for DENV, with a 13-fold increase, and ZIKV, with a 5.6-fold increase."

R1.15: is generally poorly phrased and is very repetitive. Can you re-phrase in a way that is more biologically relevant? For example, what I think you are trying to say is that it is not realistic to expect some mosquitoes to immediately become infected/ infectious. I think you can state this much more succinctly and just refer to the figure?

The suggestion has been taken into account and we rephrased the manuscript.

Lines 164-165: "We have shown that it is often unrealistic to assume a large proportion of mosquitoes to become immediately infectious after being infected, as presumed by the exponential distribution."

R1.16:
Fig 1. All the data in here should stand alone so it can be interpreted without referring to the main text or supplementary material (this also goes for the other figure) :
- Spell out abbreviations, and what does 'scenario' mean?
- 'Selected proportion of model' should be re-phrased…

- 1i) – it is hard to interpret – what do the colours correspond to? It says one colour per scenario – but grey is used for five scenarios? It would also be useful to be clear what you mean by scenario – here are you referring to a single vector competence experiment? If so just call it experiment or data set as' scenario' is vague.
- Please state what a selected scenario is in this legend.

All the suggestions (R1.16) have been taken into account and the caption has been modified as such: Lines 191-201: "Fig 2.Inference of the duration distributions of the intra-vector infection dynamics (IVD) stages for all scenarios*. A: chikugunya virus (CHIKV); B: dengue virus (DENV); C: Zika virus (ZIKV). (i): Proportion of selected models among those tested (IbetaDexpo, IbetaDbeta, IexpoDexpo, IexpoDexpo). Scenarios with 5 or more day post-exposure (Dpe) are presented in colour (one colour per scenario), others are in grey. (ii): Average of the selected distributions in the infected state for the main selected model (only scenarios with 5 or more Dpe are represented). (iii): Selected dynamics in the infected state for the main selected model (only scenarios with 5 or more Dpe are represented). Dots: observed data; lines: mean dynamics; uncertainty ribbons (5%-95%): selected simulated dynamics. *One scenario consists of a vector competence experiment on female mosquitoes from a specific genus, species and geographical origin, exposed to a virus isolate from a given species at a single infectious dose (log_{10} FFU/mL) in the blood meal."

R1.17: iii) What does 'effectif' mean?

It has been replaced by "proportion of mosquitoes (%)"

R1.18: Please can you explain what greater insight your model provides concerning the evidence that the barriers are not systematically crossed, beyond what is already in the data? If by the end of an experiment, not all mosquitoes have become infected/ are able to transmit – that seems self-evident? Or perhaps I am misunderstanding.

We agree with your statement. The message we wanted to get across in this paragraph was not well expressed. In fact, the advantage of our model is that it simultaneously estimates the proportions of mosquitoes in which the viruses do not cross each of the three barriers and the distribution of the duration in each of the two stages between viral exposure and transmission. In addition, our model makes it possible to quantify the uncertainty surrounding the value of the barrier parameters, which is not possible with data that provide a single value. The study of this uncertainty allowed us to identify the impact of the mosquito sample size on this uncertainty. We have modified the paragraph and its title to support this message.

Lines 203-205: « Title : Uncertainty of the intra-mosquito barrier-crossing estimation depends on the mosquito sample size."

This allowed us to provide recommendations in the discussion:

Lines 380-391 : "For half of the scenarios studied, the sample size used in the vector competence experiments used to infer the model was sufficient to obtain a low uncertainty in our results. However, when the barriers to infection or dissemination were high, the number of mosquitoes in the final stage was insufficient to achieve inference results with low uncertainty. Our approach could be used to determine in advance how many mosquitoes should be sampled at each time point to ensure gathering sufficiently robust information, assuming we have a preliminary idea of the concerned IVD dynamics. For example, we suggest that experimenters increase the number of mosquitoes sacrificed at intermediate and final time points of the experiment, where the transmission barrier is often crossed. This is particularly important for virus/mosquito/dose conditions where low values are expected for the infection and transmission barriers. This approach would ensure a sufficient number of mosquitoes to accurately study the duration distribution in the disseminated state and the dynamics of crossing the transmission barrier."

R1.19: Fig 2. 'values of the crossing barrier parameters' – would suggest to re-phrase so that it is clearer e.g. something like 'Parameter value estimates for the probability of crossing the infection and dissemination barriers' ?

The suggestion has been taken into account and added to the text:
Lines 234-241: "Fig3. Parameter estimates for the probability of crossing the infection, dissemination, and transmission barriers for all scenarios* tested for chikungunya virus (CHIKV), dengue virus (DENV) and Zika virus (ZIKV). A: Infection barrier; B: Dissemination barrier; C: Transmission barrier. Each bar represents the parameter mode for a given scenario, with one colour per scenario and with the 90% credibility interval represented by the error bar. *One scenario consists of a vector competence experiment on female mosquitoes from a specific genus, species and geographical origin, exposed to a virus isolate from a given species at a single infectious dose (log10 FFU/mL) in the blood meal."

R1.20: Yaxis label modegammaD – please just state 'parameter estimate for (parameter symbol)' etc without the 'mode', again instead of 'scenario' explain explicitly what that is -.e.g dataset or experiment.

The suggestion has been taken into account and added to figure 3.

R1.21: This statement seems overly obvious and is applicable to all modelling exercises that use data for calibration or fitting.

We agree with your statement. The message we wanted to get across in this paragraph has not been properly expressed. To make the paragraph clearer, it has been split in two. One part has been placed in the previous paragraph to support the study of the uncertainty surrounding the estimation of the values of the barrier parameters. The other part has been retained and the title of the paragraph changed. It now describes the contribution of inference to the exploration of hypotheses about the mechanisms possibly involved in IVD.

Lines 244-245: "Title: Model fit quality reveals underlying intra-vector dynamics and assumptions.
Lines 256-265: "The decrease in inference quality questions the processes assumed in IVD. Indeed, fit quality reduced in scenarios where the observed dynamics deviates from the expected dynamics, which is first a decrease in the infected state, followed by an increase and subsequent decrease in the disseminated state, and finally a continuous increase in the transmitter state. In 6 scenarios (DENVc1, DENVc2, ZIKVc4, ZIKVc5, ZIKVc6, ZIKVc10), the proportion of infected mosquitoes initially increased but then decreased, while in 9 scenarios (CHIKVc1, CHIKVc2, CHIKVc5, CHIKVc6, CHIKVc7, CHIKVc8, DENVc6, DENVc7, ZIKVc1) the proportions in the transmitter state first increased and then decreased (Table S4.10 in S4 Appendix). These deviations likely reflect biological processes not captured by our model, which assumes a unidirectional progression of mosquitoes through states from which return is impossible as classically acknowledge by entomologists (30)."

R1.22: '*the quality of our model inference was satisfying based on an adequate visual fit between observed and simulated data for the selected models'… 'satisfactory visual fit quality'*. Please try and reduce vague statements.
R1.23: '*a decrease in the fit quality was observed when the dynamics of the infected, disseminated and transmitter states deviated from a 'classic' dynamics (i.e. increasing in the infected state, increasing and then decreasing in the disseminated state, and increasing in the transmitter state'* – what is classic dynamics? Use of parentheses generally indicates poorly phrased sentence and what exactly is increasing and decreasing here?
R1.24: What do you mean by '*up to date assumptions*'?

The three suggestions (R1.22, R1.23, R1.24) have been taken into account and the text modified (Lines 246-265, and see R1.21)

R1.25: what do you mean here by low uncertainty – can you be quantitative…. A narrow 90% CI – how narrow?! And on only half of scenarios? I am really not sure what is being communicated here in these next few sentences or why.

We modified the paragraph to make it clearer and moved this part on section: "Uncertainty of the intra-mosquito barrier-crossing estimation depends on the mosquito sample size" . (Lines 223-231)

Methods

R1.26: I think it would help in this section if you avoided using so many parentheses. For example, '*To infer the distribution of the IVD stage durations, we divided respectively the I and D compartments into n and m sub-compartments of 1-day duration each (n and m thus being the maximum length of stay, in days, in I and D).*' Could be written as 'To model the distributions of the time to dissemination and time to transmission, we divided the infection and dissemination compartments into *m* and *n* sub-compartments. The number of *m* sub-compartments was equivalent to the duration of the experiment and *n* was equivalent to one minus the duration.

The suggestion has been taken into account and the text has been modified in "Model design"

Lines 488-490: "To model the distributions of IVD stage durations, we divided the infection and dissemination compartments into *n* and *m* sub-compartments, with *n* the duration of the experiment in days, and *m* this duration minus one day."

R1.27: If the model consisted of one compartment per IVD stage, and went on to mention four compartments (E, I, D, and T), why were three stages of IVD mentioned in L402? It is unclear

The suggestion has been taken into account and added to the text.

Lines 472-474: "To represent the barriers and stages of IVD and to assess the distribution of residence times in the infected and disseminated states, we developed a discrete-time stochastic mechanistic compartmental model with a one-day time step (Fig 1)."

R1.28: "When crossing a barrier …" consider starting this sentence in a new paragraph

The suggestion has been taken into account (line 496).

R1.29: "mosquitoes were distributed among the sub-compartments …". Were they randomly distributed based on the output of multinomial distribution? If yes, make it clear by adding the word.

The suggestion has been taken into account and added to the text.

Lines 496-498: "When crossing a barrier and entering a new IVD stage (I or *D*), mosquitoes were randomly distributed using a multinomial distribution among the sub-compartments of this new stage to represent the duration distribution in the stage."

R1.30: – First it is unclear how N_{tot} relates with other compartments. Would be good to include it in model diagram or describe it in the caption. Secondly, it is mentioned that mosquito size varies between observation dates and was adapted based on experiment data in each scenario. But how this was done in the simulation (e.g., initial population size or adjusted over time) could be clarified.

The suggestion has been taken into account and added to the text.

Lines 505-509: "Due to mortality, the number of sampled mosquitoes varied across observation dates. Thus, we introduced parameter N_{tot}, representing the number of mosquitoes sampled at each observation date, adjusted for each scenario and observation date to match experimental data. This parameter was integrated into the data-driven observation layer of the model, enabling to estimate the number of mosquitoes per compartment per observation date."

R1.31: Consider mentioning the acceptance criteria (e.g., threshold(s) used) and the number of accepted particles.
R1.32: "providing that all four criteria were met" in what way? Do you mean if sum of squares (a single value) is less than the final threshold specified? If yes, then the sentence is a bit confusing with the phrase "all four criteria". If you were referring to something different, would be good to make it clear.

The two suggestions R1.31 and R1.32 have been taken into account and added to the text.

Lines 537-542: "For each particle, we calculated four distances, one for each of the four IVD states, defined as the sum of the squared errors between the observed and simulated data for the different Dpe. A particle was accepted if each of the four associated distances met its own acceptance criteria, i.e., was below the respective threshold. The thresholds for these criteria were recalculated at each

inference round, using the 90th quantile of particles accepted in the previous round. In each round, 800 particles were accepted."

Discussion

R1.33: '…*do not hold in most situations with regards to the mosquito and virus genotype and or infectious dose'* – I don't understand how you can make this conclusion as you do not account for these factors in your modelling – do you mean irrespective of these factors? Even then, actually, at very high virus dose, these assumptions may hold, I would argue.

We agree that our initial formulation did not fully capture what we have demonstrated in this study. The estimation of the distributions used to simulate the data as realistically as possible was carried out under specific experimental conditions. Due to the lack of data, it is not possible to establish a functional link between biotic factors (e.g. mosquito and virus genotype or infectious dose) and our estimated parameters. However, our results highlight that the exponential distribution is less frequently selected than the beta distribution. Especially for DENV and ZIKV, for more than 90% of the experimental conditions studied, at least one of the two main distributions selected in the infected or disseminated state was not exponential. More importantly, our results also highlight the need to adapt the choice of distribution to the biotic factors at play in order to improve the realism of epidemiological models. We have reworded our paragraph to better explain that our results were lead to experimental conditions.

Lines 272-276: "For these three major arboviruses, this study simultaneously estimates the parameters of intra-vector infection dynamics : the distribution of the durations of the two key stages of IVD (midgut infection and dissemination in the circulatory system) which are often non exponential under experimental conditions considered, and the proportions of mosquitoes in which the viruses cross each of the three within-mosquito barriers.»

R1.34: with respect to parameterising transmission models from experimental data – this accounting for nuances in distributions may only be relevant if asking relative questions e.g. comparing between vector species because data from laboratory experiments often do not reflect 'true' values. For example, we know that the probability of infection is higher and the EIP shorter when mosquitoes feed on a host rather than artificial bloodmeal.

We discuss this limitation in the discussion lines 354-362 : "One limitation of our study is that data used to infer model parameters come solely from laboratory experiments, which sometimes are far from field conditions (38). Indeed, parameters governing IVD might be influenced by the type of blood feeding (artificial versus on an infected animal), as it is the case for mosquito infection rates (39). Here, we used data from experiments performed with artificial blood feeding, due to a lack of suitable data from experiments performed with blood feeding on infected vertebrate hosts. Such experiments are scarce and challenging to conduct due to evident ethical and logistical constraints (40,41). However, for the rare cases where such data is available, our modelling approach can be used without any change to improve knowledge on IVD while increasing model accuracy."

We have added sentences to support this point of vigilance.

Lines 288-289: "However, it is important when interpreting the results, to keep in mind that these are laboratory experiments, which may differ from field conditions."

R1.35: this simply isn't true, usually this is accounted for using transmission probability parameters.

We agree with you that intra-mosquito barriers are taken into account in epidemic models in the host-to-mosquito and mosquito-to-host transmission parameters. We deleted this assertion in the text.

Reviewer #2: Overall, I enjoyed reading this manuscript but there are some minor revisions I recommend.
Thanks for your supportive comments and your help in improving our paper.

Intro

R2.1: I find the purpose of this paper to be poorly explained, it is well-known that only a small fraction of arbovirus infections in mosquitoes end up being transmitted. There are decades of empirical data showing this, this paper tries to argue that this is a new finding. I think the authors need to be more careful in the phrasing they use to describe to purpose and results of this manuscript.

We concur with your assertion. We made the purpose and results of this manuscript more precise and better argued with regards to the novelty of our work (see also the first page of this response letter).

Line 111-126: "We aimed to better characterise the IVD for DENV, ZIKV, and CHIKV, three arboviruses of major public health importance. To evaluate the relevance of assuming an exponentially distributed extrinsic incubation period (EIP), we developed a stochastic mechanistic model of IVD. This model explicitly represents the distribution of IVD stages and the crossing of the three within-mosquito barriers. To examine how these processes vary according to biotic factors - such as virus species or strains, mosquito species, and infectious doses, we calibrated the model using a comprehensive dataset of published experimental results under comparable protocols.

Our analysis simultaneously estimated the proportions of mosquitoes in which viruses fail to cross each barrier and the distribution of durations in infection and dissemination stages. The results revealed significant deviations from the exponential assumption, underscoring the importance of using more realistic IVD stage distributions. Furthermore, the framework produced conjoint coherent parameter estimates that allow for the explicit integration of experimental results into broader host-vector transmission models. This approach facilitates the representation and exploration of links between intra-vector processes and the larger-scale dynamics of arbovirus transmission. By enhancing the robustness of existing epidemiological models, this new knowledge could contribute to improved epidemic forecasting and more effective public health interventions for mosquito-borne diseases."

R2.2: During the introduction the authors refer to compartment often , please define this compartment.

R2.3: The reviewers need to note in their manuscript that EIP is not solely used to depict vector-borne disease transmission, it is typically accompanied by infection, dissemination, or transmission rates to calculate the R0 or the vectorial capacity.

The two suggestions R2.2 and R2.3 have been taken into account and the sentence has been amended to emphasise that the compartment referred to is that of a transmission model.

Lines 85-88: "Most compartmental transmission models do not explicitly represent IVD, but provide an approximation with a single compartment whose entry rate depends on infection, dissemination and transmission rates, and whose exit rate is assumed to be constant and equal to the inverse of the mean EIP (12,20)."

Results

R2.4: This manuscript is currently formatted where the results come before the methods, if you intend to keep the manuscript formatted this way the authors should better explain the model in the results section.

The suggestion has been taken into account and model and method descriptions have been added in the first part of the results.

Lines 132-140: "Using vector competence data available in the literature for DENV, ZIKV, and CHIKV, we inferred the parameters of our IVD model (Fig.1 and Table 1) using an Approximate Bayesian Computation - Sequential Monte Carlo (ABC-SMC) approach combined with model selection. Specifically, we compared models using either a beta or an exponential distribution for the durations in

the infected and disseminated states. We studied 43 scenarios, each consisted of a vector competence experiment on female mosquitoes from a specific genus, species and geographical origin, exposed to a virus isolate from a given species at a single infectious dose (log10 FFU/mL) in the blood meal (Fig. S2.2 and Tables S2.1 and S2.2 in S2 Appendix). As the parameters of our IVD model were estimated jointly, our study provides a consistent set of parameters for the experimental conditions studied. "

R2.5: Describe what you mean by "scenario" in the results, methods, and figure legend. It would seem from your supplemental that it is data from one manuscript, but this needs to be well described in the main text.

The suggestion has been taken into account and scenario definition has been added in results, methods and figure legends. (see R2.4)

R2.6: Fig 1 panel i is hard to understand, I recommend authors change the histogram pattern rather than just the direction of the lines and add a black line to denote a break in the bar

Black lines have been added to denote a break in the bar and patterns have been changed as suggested. (see Line 188, Fig 2)

R2.7: Have the authors investigated how different strains, and populations (colony vs field) change their model?

We would have liked to investigate the influence of such biotic factors on IVD. However, this is not currently feasible given the limited amount of available data, which makes it impossible to statistically assess the influence of biotic factors on parameter estimations. We discuss this in the discussion.

Lines 326-330: "However, this would require a considerable amount of additional data in order to be able to study independently and statistically the possible influence of biotic factors. Such a functional link would be essential to account for an IVD varying with biotic factors, themselves varying in space and time, and could significantly impact model-based recommendations to public health stakeholders."

Methods

R2.8: Why was such a small number of research articles chosen for the model? I would appreciate the authors outlining the reason they picked these specific manuscripts and not others in the methods of the manuscript rather than a graphical depiction in the supplemental. I can think of several other mosquitoes on DENV/ZIKV infection in mosquitoes that were not included, wouldn't more scenarios help improve your model?

The suggestion has been taken into account and a description of articles chosen has been added to the text in "Experimental data" section.

Lines 443-458: "To calibrate our model, relevant vector competence experimental data were collected by an extensive literature search (Fig.S2.1 in S2 Appendix). We used four search databases (WOS core collection, Scopus, Pubmed and Medline) with the following query: ("arthropod-borne" OR arbovirus) AND (competence OR "vector competence" OR "intra-mosquito" OR "intra-vector" OR dissemination OR transmission OR "systemic infection" OR "extrinsic incubation period" OR dynamic) AND virus AND ("in vivo" OR empirical OR experimental OR saliva OR "vector competence assays") NOT tick. This query returned 2197 records. After deleting the duplicates, we examined the articles by title and deleted 815 of them. We then examined 239 articles on the basis of their abstracts and excluded articles dealing with arboviruses other than CHIKV, DENV or ZIKV or not including experiments on vector competence. We also chose to exclude articles that were not in open access because we wanted to encourage open science. Next, we screened the remaining 102 articles and removed articles with only one IVD stage studied, articles with only figures and no accessible tables or raw data, and articles with fewer than 4Dpe in order to increase our chances of having a sufficient number of time-points to capture the dynamic aspect of IVD. This search leading to 15 articles (12,18,25,29,46,47,50–58) (Tables S2.1 and S2.2 in S2 Appendix) encompassing a total of 43 scenarios."

R2.9: If the main point of this paper is the generic model then the model should be included in the main text of the MS. I recommend the authors move figure S2.2 to the main text and add more detail about the model to the figure

We have moved the model figure to the main text at the beginning of the results, as the generic model is indeed a key aspect of our study (line 143 Fig 1 and line 156 Table 1). Figure S2.2 in S2 appendix illustrates the experimental protocol used to generate the data used for model inference. This figure is a summary of the protocol used in the articles find in literature and not our own work. This figure is therefore included in the supplementary material to complete the 'experimental part' of the method section, but is not a major component of our study. For this reason, we feel that it is more appropriate to keep Figure S2.2 in the supplementary material.

R2.10: Fig S2.2 is very confusing, what does the number of lines mean on the left side of the figure, please rewrite the figure legend.

The number on the left side of the figure was the number of mosquitoes killed per batch at each time point during the experiment. This number varied between 20 and 60 according to the experiment. Figures have been modified to increase the clarity of this aspect (Figure S2.2 in S2 Appendix).

Discussion

R2.11: What other abiotic factors should be tested ?

Our intention here was not to suggest testing additional biotic factors but rather to highlight the need for more experimental data to allow studying already identified biotic factors. This sentence partially reiterated ideas presented earlier and was primarily intended to transition to the following paragraph. To avoid mixing multiple ideas within a single paragraph, we have removed the mention to biotic factors already discussed above and refined the text for clarity.

Lines 334-336: "Hence, further laboratory work is needed using similar experimental procedures, associated with an increased access, sharing and standardization of raw experimental data."

R2.12: A lot of mosquito studies have a sample size of 40-50 mosquitoes per treatment (line 328), 20 is the lowest you can go and is rarely used now, please correct this.

The sentence has been modified.

Lines 380-382 : "For half of the scenarios studied, the sample size used in the vector competence experiments used to infer the model was sufficient to obtain a low uncertainty in our results."

R2.13: One of the major caveats of this MS is that the authors state they only used open-access papers with data available. People rarely publish their raw data, but one can often infer from the tables in the manuscript, especially in vector biology manuscripts where the data is described in detail (n, n infected, n disseminated, n transmitting, and dpi) (line 259). Have the authors thought about running the model with non-open access manuscripts and does that change the result?

We used all the open access articles that provide tables or figures from which the necessary data could have been extracted. Articles that only present graphs without numerical data or lack detailed tables specifying the number of mosquitoes per state could not have been included. As part of a public research institute that promotes open science, we prioritized open access articles to ensure transparency and reproducibility. While we aimed to be as exhaustive as possible within these constraints, we acknowledge that the dataset may not cover all existing studies. We have added details about literature searching procedure on "Experimental data" section . If you are aware of any missing article that fulfils our conditions, we would be more than happy to add it in a revised version of our article.

Line 449-456: "After deleting the duplicates, we examined the articles by title and deleted 815 of them. We then examined 239 articles on the basis of their abstracts and excluded articles dealing with arboviruses other than CHIKV, DENV or ZIKV or not including experiments on vector competence. We

also chose to exclude articles that were not in open access because we wanted to encourage open science. Next, we screened the remaining 102 articles and removed articles with only one IVD stage studied, articles with only figures and no accessible tables or raw data, and articles with fewer than 4 days post-exposure (Dpe) in order to increase our chances of having a sufficient number of time-points to capture the dynamic aspect of IVD.”

Reviewer #3: The manuscript submitted by Loisel et al describes a compartmental model to better predict the probability that exposed mosquitoes become infectious during the extrinsic incubation period for a variety of arboviruses. The authors use data found in existing literature to evaluate the ability of their compartmental model to predict experimentally-derived infection probabilities. While the model presented fits the experimental data well and provides a useful framework for modeling the probability of infectious mosquitoes for better understanding transmission, there is some confusion in how the proposed model fits in with existing frameworks used to model vector-borne disease transmission. For example, in most equations of vector-borne disease transmission such as R0 or vectorial capacity, in addition to EIP there is usually a vector competence variable to describe the probability of an exposed mosquito to become infectious. The authors don't seem to emphasize how this has been done already in the introduction and discussion and make it seem like EIP is used independently of vector competence in many transmission models when this is not the case. I think the manuscript needs to be revised to better acknowledge how the authors' newly presented model with three compartments in IVD compares to typical presentations of a single parameter for vector competence in the equation for R0 and vectorial capacity instead of only comparing to one exponential approach vs the author's approach. Does the work presented here allow researchers to better estimate the vector competence variable already used in most transmission models? Or is it independently used to estimate vector competence for a different type of transmission model? Some clarity on how this new framework fits in with existing frameworks rather than just a single variable within other frameworks (EIP) is needed. For example R0 for vector-borne diseases typically is described as (N(ah)2bcpn)/H(-ln(p))r where N represents vector abundance, a the daily biting rate of the vector, h the proportion of bites by vectors on susceptible hosts, b represents the per bite probability of a vector becoming infected given exposure, c is the per bite probability of a host becoming infected given exposure, p is the vector daily survival rate, n is the extrinsic incubation period of the parasite in days, H is the abundance of susceptible hosts in the population, and r is the daily recovery rate of infected hosts. I understand that EIP could be a distribution and assumes that all mosquitoes have the same EIP but variation in mosquitoes becoming infectious is already accounted for elsewhere in the R0 equation by the variables p and b. It would be helpful if the authors could explain why the three compartments of IVD are more useful to have than a single compartment currently in use in most models. As the manuscript currently reads, there is more focus on the EIP which is a separate variable from vector competence and should be more clearly explained. Below, I've made some suggestions line by line for where I think additional improvements in this manuscript could be made. Overall, I think it is well written and the model is presented as a useful tool for better predicting vector transmission dynamics.

First, we would like to thank reviewer #3 for its comments and help in improving our paper.

Concerning questions asked in introduction:

-" Does the work presented here allow researchers to better estimate the vector competence variable already used in most transmission models? Or is it independently used to estimate vector competence for a different type of transmission model? "

-> We answered this question in R3.1 and R3.2.

- "Why the three compartments of IVD are more useful to have than a single compartment currently in use in most models"

-> We answered this question in R3.8.

Major edits:

R3.1: There are examples where vector competence is not represented as the inverse of EIP, discuss those examples and how those examples perform compared to the framework you present here.

Of the recently published vector-borne disease models, almost none assume a non-exponential EIP distribution, and the majority represent vector competence as the inverse of EIP. Some papers suggest replacing the exponential distribution with a gamma (https://www.ncbi.nlm.nih.gov/pmc/articles/PMC4397933/) or log normal distribution (https://www.ncbi.nlm.nih.gov/pmc/articles/PMC3511440/). In our model, we needed a finite support

distribution to extract the proportions, which naturally led us to choose the beta distribution rather than the gamma or log-normal distributions. We explain this in the "method' section:

Lines 499-501 : "The beta distribution allowed us to obtain a wide range of shapes depending on α and β values, without the need for prior knowledge. This distribution being defined on the interval [0,1], it was well suited to represent proportions.".

Furthermore, in our introduction, we cited three studies that used transmission models to investigate how the distribution of different periods, including the EIP, influences epidemic dynamics in hosts.

Lines 91-96 : "In vector-borne diseases as well, the selected EIP distribution can impact virus transmission between vector and hosts, e.g. by inducing strong variations in the basic reproductive ratio as shown for bluetongue (23) or by shaping the height and timing of hosts epidemic peak (12) and the early increase in infected hosts (24) for dengue. It is therefore of interest to examine the distribution of the duration of EIP to be used in epidemiological models, with a view to best representing this distribution and offsetting any potential impact on transmission."

This highlights the importance of investigating the actual EIP distribution for different arbovirus and experimental conditions, as we have done in our study. However, at this stage we cannot directly compare our framework with other representations of the EIP in vector-borne disease transmission models, as we have not yet integrated it into such a model. Our work focuses on characterising the distribution of IVD stages and associated barriers, rather than modelling the complete transmission process between vectors and hosts. Considerable effort would be required to couple our model to a vector-borne disease transmission framework, which is beyond the scope of this paper.

Such future work could include computing Ro for a transmission model incorporating our IVD framework. The standard Ro formula used in most transmission models relies on an exponential EIP assumption, which does not hold in our case. Therefore, additional work would be needed to derive and compare a new Ro formulation with the classical one. While an important avenue for future research, this falls beyond the scope of the present study. We have specified this future direction in the discussion:

Lines 306-310 : "Furthermore, given that most epidemiological models rely on the assumption of an exponential EIP to calculate R0, incorporating our framework into these models would require a new formulation of R0, as the standard expressions would no longer apply. While some studies have considered the impact of non-exponential EIP distributions on transmission potential, this remains an important avenue for further research."

R3.2: I think it might be useful to show the big picture transmission model most researchers use to represent transmission and which part of that model the work you present here impacts. For example, the authors call out the EIP parameter but I think the work here more directly predicts the vector competence parameter. Provide an explanation for how these variables relate and which part(s) of transmission models the authors are improving in this study (i.e. estimates of vector competence by using a 3 compartment model). Is it the EIP parameter or the vector competence parameter and how it varies across EIP?

We have added a figure to the supplementary data that situates our model within the broader context of vector-borne transmission models and we cites this figure in discussion:

Line 302: "A critical contribution of our model is that it allows conjointly relaxing the exponential assumption and explicitly represented the intra-mosquito barriers if used as a building block in larger population-scale epidemiological models (Fig S2.3 in S2 Appendix)."

We chose to include this figure in the supplementary data rather than in the main text as we have not yet performed a full integration of our framework into a broader transmission model, which is beyond the scope of the present study. Including this figure in the main text could confuse readers towards the central message of our article on advancing the understanding and representation of IVD. Our approach explicitly accounts for the distributions of the various stages that mosquitoes progress through during the EIP (infection, dissemination) before being able to transmit the virus. Additionally, our model

incorporates the crossing of intra-mosquito barriers, which can be considered as factors contributing to vector competence. These barriers, often integrated implicitly into the vector-to-host or host-to-vector transmission rates in epidemic models, are explicitly modelled here. We have modified the introduction to better explain the purpose of our study.

Lines 111-126: "We aimed to better characterise the IVD for DENV, ZIKV, and CHIKV, three arboviruses of major public health importance. To evaluate the relevance of assuming an exponentially distributed extrinsic incubation period (EIP), we developed a stochastic mechanistic model of IVD. This model explicitly represents the distribution of IVD stages and the crossing of the three within-mosquito barriers. To examine how these processes vary according to biotic factors - such as virus species or strains, mosquito species, and infectious doses, we calibrated the model using a comprehensive dataset of published experimental results under comparable protocols.

Our analysis simultaneously estimated the proportions of mosquitoes in which viruses fail to cross each barrier and the distribution of durations in infection and dissemination stages. The results revealed significant deviations from the exponential assumption, underscoring the importance of using more realistic IVD stages distributions. Furthermore, the framework produced conjoint coherent parameter estimates that allow for the explicit integration of experimental results into broader host-vector transmission models. This approach facilitates the representation and exploration of links between intra-vector processes and the larger-scale dynamics of arbovirus transmission. By enhancing the robustness of existing epidemiological models, this new knowledge could contribute to improved epidemic forecasting and more effective public health interventions for mosquito-borne diseases."

R3.3: "Although the existence of these barriers is already known (33), the majority of epidemiological models of vector-borne disease transmission does not take them into account and assumes that all exposed mosquitoes eventually become infectious" – in most models of R0 or vectorial capacity for vector-borne diseases, there is a vector competence term that is not assumed to be 1. For example, see Smith DL, McKenzie FE, Snow RW, Hay SI (2007) Revisiting the Basic Reproductive Number for Malaria and Its Implications for Malaria Control. PLOS Biology 5(3): e42. https://doi.org/10.1371/journal.pbio.0050042, where c is used to describe the probability that a mosquito becomes infected given a bite on an infectious human. There are many papers in the malaria context where transmission models contain parameters to describe the probability that a mosquito becomes infected and most assume that this probability is not 1 and varies due to the barriers already mentioned in your manuscript. There is also substantial literature which already acknowledges variation in transmission probabilities due to variability in the barriers or things that impact these barriers, for example see Lefevre T, Ohm J, Dabiré KR, et al. Transmission traits of malaria parasites within the mosquito: Genetic variation, phenotypic plasticity, and consequences for control. Evol Appl. 2018; 11: 456–469. https://doi.org/10.1111/eva.12571. Please acknowledge the approach others have taken to incorporate variation in vector competence in transmission models here and compare your method to methods previously used. In Aedes, there are equally plentiful examples of prior work which acknowledges variation in vector competence which is accounted for in the vectorial capacity equation, for example: Effect of Larval Competition on Extrinsic Incubation Period and Vectorial Capacity of Aedes albopictus for Dengue Virus Bara J, Rapti Z, Cáceres CE, Muturi EJ (2015) Effect of Larval Competition on Extrinsic Incubation Period and Vectorial Capacity of Aedes albopictus for Dengue Virus. PLOS ONE 10(5): e0126703. https://doi.org/10.1371/journal.pone.0126703.

We concur with your assertion that the non-systematic crossing of intra-mosquito barriers is taken into account in numerous transmission models by host-to-mosquito and mosquito-to-host transmission rates. What is novel in our approach is to jointly estimate the proportions of mosquitoes in which viruses cross each of the three barriers and the distribution duration in the associated waiting stages. We thus modified the text to be more precise. We also compared in discussion our method to methods previously used.

Discussion, lines 291-300: "Second, concerning within-mosquito barriers, our modelling approach allowed representing and quantifying the probabilities of viruses crossing each of these barriers as a function of experimental conditions. We highlighted that the transmission barrier was the most challenging to cross regardless of the mosquitoes, viruses and infectious doses considered (29). Such barriers are already accounted for in most vector-borne disease transmission models by the host-to-mosquito transmission rate. However, the value of this rate is usually chosen independently of the EIP

duration (e.g., with values taken from different articles), while it is probable that these two parameters are correlated. Our modelling framework offers the possibility to estimate the three barrier-crossing parameters directly from the experimental data, and jointly with the duration distributions of the infected and disseminated states, thus providing a consistent parameter set and consolidated information."

In addition, our model makes it possible to quantify the uncertainty surrounding the value of the barrier parameters, which is not possible with data that provide a single value.

Concerning the impact of biotic factors on barriers, we moved this section to discussion.

Lines 312-319: "Besides influencing the EIP duration (10,14), biotic factors (mosquito and virus genotypes, and viral infectious dose) also impact the distributions of residence time in IVD stages, as well as the probabilities of crossing intra-mosquito barriers (10). However, for establishing a quantitative statistical link between such biotic factors and IVD parameters, much more experimental data are needed, which currently are not available in the literature. Nevertheless, the variety of experimental data already available - and used in our study - enabled us to propose a modelling framework easy to use on novel data once available. This framework leads to estimate consolidated parameter sets according to such biotic factors and usable in epidemiological models to better assess outbreak management measures."

R3.4: "considerable additional amount of data would be required" – what data is required? Provide recommendations for what type of data need to be experimentally collected and why to help readers understand the what and why of this statement. You mention some of these things later in the discussion, but would be good to list earlier.

R3.5: Could the authors provide specific examples of the types of data and experiments that are currently lacking but would be informative to the field?

We have detailed the type of data missing and needed to improve knowledge of the IVD in the discussion to answer questions R3.4 and R3.5.

Lines 326-334: "However, this would require a considerable amount of additional data in order to be able to study independently and statistically the possible influence of biotic factors. Such a functional link would be essential to account for an IVD varying with biotic factors, themselves varying in space and time, and could significantly impact model-based recommendations to public health stakeholders. Although numerous studies provide experimental data on vector competence, data enabling the estimation of the dynamic aspect of the IVD - specifically studies tracking infected, disseminated and transmitted states across a sufficient number of time steps (≥ 4 in our study) - remain relatively scarce. Out of the 91 articles with vector competence data on CHIKV, ZIKV, and DENV analysed in our study, only 9 met this criterion."

R3.6: Similarly, what would the proposed standardized data format be? What sorts of standards would the authors recommend?

We proposed to follow the recommendations made by Wu et al in their study "A minimum data standard for vector competence experiments" 10.1038/s41597-022-01741-4. We have modified the sentence in our text to make this clearer.

Lines 348-350: "Sharing raw and standardized data as proposed by Wu et al (35) following the FAIR (Findable-Accessible-Interoperable-Reusable) principle (36) is crucial for increasing data value and usefulness (35)."

R3.7: It would be very useful to experimental biologists to be given more clear recommendations on things like sample sizes for experiments – I wonder if the authors could provide examples of experimental designs they would recommend for different types of experiments based on the model results and their experience using the existing data in the literature to support this recommendation

We have added sentences in the text to propose such a recommendation.

Lines 386-391: “For example, we suggest that experimenters increase the number of mosquitoes sacrificed at intermediate and final time points of the experiment, where the transmission barrier is often crossed. This is particularly important for virus/mosquito/dose conditions where low values are expected for the infection and transmission barriers. This approach would ensure a number of mosquitoes sufficient enough to accurately study the duration distribution in the disseminated state and the dynamics of crossing the transmission barrier."

R3.8: why is it better to compartmentalize IVD into 3 stages instead of just having one transmission probability variable?

We decided to represent the three IVD stages to be able to estimate separately the distributions of residence times in the infected and disseminated states. Furthermore, as the factors influencing the crossing of intra-mosquito barriers are not always the same according to the barrier, such a model enables to distinguish the potential impact of variations in biotic factors on the distributions in these two states. We have modified the sentence in the text.

Method, Lines 472-474: “To represent the barriers and stages of IVD and to assess the distribution of residence times in the infected and disseminated states, we developed a discrete-time stochastic mechanistic compartmental model with a one-day time step (Fig 3).”

And we have added a potential future use of this model who represents the different IVD stages and barriers.

Discussion, Lines 320-323: “In addition, as our model explicitly represents the different stages of the mosquito during IVD (infected, disseminated, transmitter), it could therefore be used to study these stages and barriers separately in a control strategy based on reducing vector competence by vaccination or genetic modification targeting one of these stages (33).”

Minor edits:

- Abstract, lines 18-20: Rephrase “To elucidate transmission, anticipate their spread and efficiently control them, mechanistic modelling has proven its usefulness.” for clarity. Suggestion: “Mechanistic modeling has proven useful to elucidate transmission, anticipate the spread, and predict the response of arboviruses to control tools.” -> Suggestion followed in the text.

- Abstract: Missing “on” in “most models rely assumptions” in cover page (but appears in abstract of main doc) : Modified in submission form.

- Abstract, line 23: change “two strong hypotheses” to “two strong assumptions”? -> Done.

- Abstract, line 24: change “To assess these hypotheses” to “To assess the validity of these assumptions” -> Done.

- Line 37: change “poses” to “posed” -> Done.

- Line 40: specify for arboviruses instead of just general epi models? -> Done: “models” has been replaced by “vector-borne transmission models”.

- change “every exposed mosquitoes” to “every exposed mosquito” -> Sentence has been deleted.

- Line 91: change “is better represented by non-exponential distribution to “is better represented by a non-exponential distribution” -> Done.

- change “infection” to “infections” -> Sentence has been modified and this term deleted.

- Figure 1: there are gray bars in A-C, ii and iii around x=day10, y=0.5. What are these designating? -> This grey bar represented the "I" letter to indicate that the figure is associated to the Infected state. This is now stated in the figure title and the letter has been deleted.

- Figure 1 caption missing (i) description. Why do "modBetaBeta, modBetaExpo, modExpoBeta, modExpoExpo" listed in caption not match models listed in top legend ("IbetaDexpo, IbetaDbeta, IexpoDexpo, IexpoDbeta")? -> In Figure 2 caption, "modBetaBeta, modBetaExpo, modExpoBeta, modExpoExpo" was replaced by IbetaDexpo, IbetaDbeta, IexpoDexpo, IexpoDexpo.

- Line 194: change "exposition" to "exposure" -> Done.

- Line 180: change "demonstrating" to "demonstrated" -> Done.

- change diseases to disease -> Sentence has been modified.

- Lines 286-288: mention what is meant by "others". Suggest "other vector-borne diseases like malaria"? -> "Used here for three major arboviruses, it can be easily used for other arboviruses such as West-Nile virus, as long as similar experimental data are available."

- Lines 291-293: change "allows to" to "allows us to" -> Sentence has been modified ,: "Second, concerning within-mosquito barriers, our modelling approach allowed representing and quantifying the probabilities of viruses crossing each of these barriers as a function of experimental conditions"

- Line 362: change "defining" to "define" -> Done.

- Lines 415-417: wording is unclear, please rephrase for clarity -> "However, modelling the exit of mosquitoes from the transmitter state would require more precise knowledge of this phenomenon to choose between a return to the disseminated state or a simple exit from the transmitter state."

- Line 430: change "This could enable to study" to "This could enable researchers to study" -> Done.

- Figure S2.2: Change "on body", "on saliva" to "in body", "in saliva" -> Done

- Figure 1: "effectif" is in French? Translate to English -> "effectif" was modified to "proportion of mosquitoes"
