# Supplementary material for: Intra-vector infection dynamics challenge how to model the extrinsic incubation period for major arboviruses: dengue, Zika, and chikungunya
Source: PLoS Comput Biol. 2025 Aug 25;21(8):e1013393. doi: 10.1371/journal.pcbi.1013393 (PMC12440223; doi:10.1371/journal.pcbi.1013393)
Supplement: Attachment — Submitted filename: response_to_reviewers_auresp_2.pdf [file pcbi.1013393.s010.pdf]

ANSWERS TO REVIEWERS

Loisel et al., submitted in PLOS CB

PCOMPBIOL-D-24-01822

Intra-vector infection dynamics challenge how to model the extrinsic incubation period for major arboviruses: dengue, Zika, and chikungunya

We would like to thank the three reviewers for their interest in our work and their useful and accurate comments that greatly helped us improve our manuscript.

We explain below how we addressed their remarks and what was changed in the text to take them into account.

Reviewer #1:

R1.1: As per one of the other reviewer's previous comments, if you are going to have the results before the methods, I think you need to give a brief description of the model in the first paragraph and refer to the conceptual figure in the text.

As recommended, we have added a brief description of the model at the beginning of the Results section, in addition of a reference to the conceptual figure 1.

Lines 137-160: "Using vector competence data available in the literature for DENV, ZIKV, and CHIKV, we inferred the parameters of our IVD model (Fig.1). The model includes one compartment for each IVD stage. Stage E represents exposed mosquitoes, when the virus is present in the digestive tract following a blood meal. Stage I corresponds to infected mosquitoes, when the virus is present in the midgut. Stage D denotes disseminated mosquitoes, when the virus has spread to the circulatory system. Finally, stage T refers to transmitter or infectious mosquitoes, marked by the presence of the virus in the saliva. To account for the non-systematic crossing of barriers (16), we introduced three parameters, γ_I, γ_D, and γ_T, to represent the proportions of mosquitoes for which the infection, dissemination, and transmission barriers are crossed, respectively. When crossing the first or second barrier and entering in IVD stage I or D, mosquitoes were randomly distributed among the sub-compartments of this new stage using a multinomial distribution to represent the duration distribution in the given stage. This distribution can be mediated either by an exponential distribution (of parameter λ) or by a beta distribution (of parameters α and β), depending on the model used. The models were called IbetaDexpo, IbetaDbeta, IexpoDexpo, and IexpoDexpo, and they differ based on the distribution used - either beta or exponential (expo) - for the infected (I) and disseminated (D) stages, as indicated by the first and second parts of their names, respectively. To perform inference, we used an Approximate Bayesian Computation - Sequential Monte Carlo (ABC-SMC) approach combined with model selection. We studied 49 scenarios, each consisting of a vector competence experiment on *Aedes* female mosquitoes from a specific species and geographical origin. These mosquitoes were exposed to a virus isolate from a given species at a single infectious dose (expressed as $\log_{10}$ (FFU/mL)) in the blood meal (Fig. S2.2 and Tables S2.1, S2.2, S2.3 and S2.4 in S2 Appendix). Depending on the experimental design, we inferred either a partial model (EID), when no information was available for the transmitter stage, or a complete model (EIDT), when the data set covered all

IVD stages. As the parameters of our IVD model were estimated together, our study provides a coherent set of parameters for the experimental conditions that we examined.”

R1.2: Subsection on lines 203-205 – I still think this is so obvious that it doesn’t require a separate subsection. You talk about uncertainty in the previous section and suggest you just merge this section.

We merged this section with the previous one.

R1.3: I think it will help too if you give a description at the start of the results about what parameters were estimated and which relate to distributions and which relate to probability of crossing.

Suggestion has been added to the text at the beginning of the result part (see R1.1).

R1.4: Why does this second section not refer to the specific viruses in the same way the previous section does?

The results presented in this section relate to the three viruses, which is why we have not referred to one virus in particular but to all three at the same time.

R1.5: The manuscript still needs a careful re-read by all co-authors and editing to ensure language is of high quality, that sentences make sense and that results are clearly explained. This will also help with making the paper more accessible to experimental biologists and not just modellers. I’ve tried to highlight this in the Introduction and Results with minor comments below, but please note that I have not done this for the Methods and Discussion – it should really be the responsibility of co-authors to do this job.

We thank the reviewer for their helpful comment regarding the clarity and accessibility of the manuscript. In response, we have carefully revised the entire text to improve the quality of the language, to ensure that sentences are clear and meaningful, and to better explain the results. To further enhance readability and make the manuscript more accessible to experimental biologists as well as modellers, we enlisted the help of a professional scientific editor. This editor conducted a thorough review and correction of the manuscript to ensure linguistic accuracy and clarity throughout. We hope that these improvements address the reviewer’s concerns and make the manuscript more broadly understandable.

R1.6: you mention competence here, but the EIP is a component of competence – also is there are relationship between vector competence and the EIP? Would advise to -re-phrase this sentence. Perhaps start by defining competence and that EIP is a component of it.

Vector competence (VC) is the intrinsic ability of a vector to get infected then subsequently transmit the pathogen during the next bite. When a vector is competent, the time (in days) needed to switch from an infected to an infectious state is the EIP. So we can consider that EIP is indeed a component (*i.e.* the time component) of VC, as it can only be calculated for the competent individuals. The two parameters (VC and EIP) are inseparable but distinct, as the first represents a two-sided qualitative phenotype (yes or no this vector will have pathogen in the saliva and how much vectors will have it at the population level) while the last is a speed (among vectors that get pathogen in the saliva, how fast did the pathogen reach the saliva). The sentence has been modified, “vector competence” has been deleted and added beside.

Lines 69-70: “A key assumption of these models is how the extrinsic incubation period (EIP), a component of the vector competence, is represented (13,14).”

Lines 72-74: “The transmission of a virus by a mosquito thus varies depending on the relationship between the EIP, the mosquito lifespan, and the biting rate.”

R1.7: Line 78: midgut not intestine -> Done

R1.8: I do think here you need to give some credit to modellers who often will use this simplification after critically assessing the impact it will have on results. I would suggest that it is the case that for any given model designed to address a specific question, that often this simplification is acceptable without it compromising the validity of the results. It might just be a case of adding a sentence that in many cases it is likely acceptable.

The sentence has been modified in this way.

Lines 97-101: “In most cases, modelers correctly assume that variations in EIP distribution will not significantly impact model predictions, although this has not been formally addressed. In light of potentially important inter-individual EIP variation among individual mosquitoes, it is worth accurately representing EIP distribution in mosquito populations, and considering its impact on pathogen transmission efficacy.”

R1.9: rather than non-exponential just state the distribution that better represents?

The sentence has been clarified.

Lines 91-93: “Yet for measles, for example, research has shown that the duration of the incubation period in hosts was better represented by a normal (21) or a gamma distribution (22).”

R1.10: not sure what you mean by ‘offsetting any potential impact on transmission’?

The sentence has been modified (see R1.8).

R1.11: integrating a wider range of experimental conditions – not sure what you mean here – into mathematical models? Do you mean enable more flexibility in how we model vector competence? I think an example would be useful here to make it clearer. If by experimental conditions you mean, for example, variation in temperature – this still can be incorporated using an exponentially distributed EIP.

We wanted to introduce here the possible use of qualitative vector-competence studies (i.e., experiments that measure the proportion of mosquitoes at each stage over time) which are more common in the literature than quantitative studies (i.e., experiments that quantify viral load in mosquitoes over time) using our model, which represents the different stages of mosquitoes over time. We decided to remove this sentence to streamline the manuscript.

Lines 110-114: “However, a generic mechanistic model of mosquito stages during IVD is still lacking. Such a model would make it possible to simultaneously estimate the proportion of mosquitoes in which viruses fail to cross within-mosquito barriers and the duration distribution of IVD stages. Typically, these parameters are estimated independently, making it impossible to account for their potential correlations in transmission models.”

R1.12: Line 118: stage -> Done

R1.13: rephase subtitle? Perhaps - The EIP of ZIKV and DENV is not exponentially distributed -> Done ,line 136: "The EIPs of DENV and ZIKV are not exponentially distributed"

R1.14: Fig 1: 'vector with virus in its digestive system into blood host' makes no sense, need to rephrase…. Midgut not intestine. Italicise mathematical symbols. -> We deleted "into blood host" and "intestine".

R1.15: missing a closing bracket. -> Done

R1.16: 'was selected' – provided a better fit to the data.

This sentence has been modified and the term 'selected' has been explained in the manuscript for greater clarity and retained in the rest of the manuscript to avoid making sentences more cumbersome.

Lines 179-181: "For the infected stage, the main distribution selected (i.e., the one the most frequently selected among those providing the best fit to the data) was the beta distribution for 7 out of 7 scenarios tested for DENV (Fig. 2Ai) and for 9 out of 15 scenarios tested for ZIKV (Fig. 2Bi)."

R1.17: Are lines 165 – 168 and 168 – 171 not saying the same thing? Perhaps delete 165 – 168?

This sentence has been deleted: "Indeed, we observed that the beta distribution provided a better fit to the data 1.7 times more often than the exponential distribution. This was particularly evident for DENV, with a 13-fold increase, and ZIKV, with a 5.6-fold increase (Fig.2)."

R1.18: what do you mean by 'partial counterpart'. What does SEID stand for – you need to spell this out in the main text on first use.

The sentence has been modified and a description of EIDT and EID has been added at the beginning of the result part (see R1.1). We deleted the letter "S" because our model do not describe this stage.

Lines 157-159: "Depending on the experimental design, we inferred either a partial model (EID), when no information was available for the transmitter stage, or a complete model (EIDT), when the data set covered all IVD stages."

Lines 185-186: "We obtained similar results when inferring the parameters of the partial EID model (Fig S3.1 in S3 Appendix)."

R1.19: of course 'low uncertainty' relates to 'high precision' they are the same thing? Either explain this quantitatively here or refer to a figure or table of results? How do you define 'low'?

The sentence has been modified to avoid repetition and to remove the imprecision of the term 'low'.

Lines 188-193: "To assess the precision of the estimated parameters governing the distributions in the infected and disseminated stages, we focused on the dispersion of the selected density distributions instead of the individual parameter values. Indeed, two very

different parameter sets can yield similar distribution shapes. For the exponential distributions, we observed a near-complete visual overlap of almost all selected density distributions for each scenario, indicating a low level of uncertainty (Figures S3.3A–S3.51A in Appendix S3).”

R1.20: when you are talking about comparison between distributions – is this between model fits to data that have the same experimental conditions on the same virus-vector combination? Or a single set of data and across the stochastic model outputs? I think this section just needs a little clearer explanation – it’s quite dense.

This part has been modified to better explain what we did. We evaluated for each scenario the uncertainty around distribution estimation in infected and disseminated states.

Lines 188-200: “To assess the precision of the estimated parameters governing the distributions in the infected and disseminated stages, we focused on the dispersion of the selected density distributions instead of the individual parameter values. Indeed, two very different parameter sets can yield similar distribution shapes. For the exponential distributions, we observed a near-complete visual overlap of almost all selected density distributions for each scenario, indicating a low level of uncertainty (Figures S3.3A–S3.51A in Appendix S3). In contrast, the visual assessment of beta distributions was more challenging. Therefore, we quantified the dispersion among all of the beta distributions selected for each scenario by applying a Kolmogorov-Smirnov test, and we calculated the percentage of statistically similar beta distributions (p-value > 0.05). In the infected stage, 13 out of 21 scenarios using the EIDT model, and 11 out of 15 scenarios using the EID model (Table S4.11 in Appendix S4), showed more or at least 50% similarity among their beta distributions. Conversely, precision appeared lower for the disseminated stage, with only 7 out of 16 scenarios exhibiting more or at least 50% statistically similar beta distributions (Table S4.12 in Appendix S4).“

R1.21: you need to spell out these abbreviations.

The sentence has been modified.

Lines 207-210 : “Proportion of selected models among those tested: IbetaDexpo, IbetaDbeta, IexpoDexpo, IexpoDexpo (these models differ based on the distribution used - either beta or exponential - for the infected and disseminated stages, as indicated by the first and second parts of their names, respectively).”

R1.22: do you mean datasets rather than scenarios – be clear what you are referring to…. Ah I’ve just seen this on 199 – you can’t have this at the end of a figure caption – you need this definition upfront at the start of the results to avoid the reader being confused! -> Definition of “scenario” has been moved on the beginning of the caption.

Lines 204-206 : “One scenario consists of a vector competence experiment on female mosquitoes of a specific genus, species, and geographical origin, exposed to a virus isolate of a given species at a single infectious dose (log_{10} FFU/mL) in the blood meal.”

R1.23: models -> Done (line 208)

R1.24: re-phrase these two sentences so it is clearer and remove ‘fit quality reduced in scenarios where the observed dynamics deviates from the expected dynamics’ – because that IS what fit is?! ‘which is first a decrease in the infected state, followed by an increase and subsequent decrease in the disseminated state, and finally a continuous increase in the

transmitter state.’ – I have no idea what this means. You can’t have an increase in a ‘state’ – what does this mean?

The sentences has been modified to be clearer.

Lines 262-274: “The lower inference quality for some scenarios nevertheless raises questions about the processes assumed in IVD. Indeed, the quality of fit decreased for scenarios where the dynamics observed in the infected, disseminated, and transmitter stages deviated from the expected IVD. Classically, entomologists assume that mosquitoes become infected within a few hours (30,31) and then progress unidirectionally through stages with no possibility of returning (32). As a result, we expect a decrease in the proportion of mosquitoes in the infected stage over time, an increase and then a decrease in the disseminated stage, and finally a continuous increase in the transmitter stage. However, in nine scenarios (DENVc1, DENVc2, ZIKVc4, ZIKVc5, ZIKVc6, ZIKVc10, ZIKVc12, ZIKVc13, ZIKVc15), the proportion of mosquitoes in the infected stage initially increased, and for eleven scenarios (CHIKVc1, CHIKVc2, CHIKVc3, CHIKVc6, CHIKVc7, CHIKVc8, CHIKVc9, DENVc6, DENVc7, ZIKVc1, ZIKVc11), the proportions of mosquitoes in the transmitter stage first increased but then decreased (Table S4.10 in Appendix S4). These discrepancies probably reflect biological processes that were not considered in our model.”

R1.25: This is for the discussion but now I see what you were trying to articulate in the previous sentences. I think you need to start with a clear description of this at the beginning of this section – essentially the data suggest that some infected mosquitoes can become uninfected? Make sure to elaborate in the discussion any evidence that this is biologically feasible (e.g. immune clearance) or could be due to diagnostic sensitivity or some other laboratory-related reason.

We already discussed this in the precedent version of the manuscript but we added some details in this revised version to make this point more precise.

Lines 424-432: “Nevertheless, recent studies on CHIKV have highlighted a reduction in the proportion of mosquitoes with the virus present in their saliva at later time points, which challenges this common assumption (13,53). The authors explain that this rare phenomenon could be a false negative detection due to an experimental issue (such as no expectorated saliva or a problem with the detection threshold). However, they also mention the possibility that the virus could be eliminated by the mosquito’s immune system or through the natural inactivation of the virions (13). As our model could not capture these dynamics, there was a discrepancy between the observed and simulated data in a few scenarios for CHIKV. Modeling the exit from the transmitter stage would require more knowledge about this phenomenon.”

R1.26: proficiency?! You been competence? -> Proficiency has been modified by “vector competence” (line 280)

Reviewer #2:

The authors of this MS set out to model how EIP is not exponential but rather a beta distribution. As stated earlier I do feel that this paper could have analyzed more data sets and is missing some of the key knowledge already shared in the field of arbovirology. The authors could be more diligent in providing a fuller picture in the discussion section, outlining the impact this will have on the field and explaining their results more clearly, more specifically what addition it is bringing to the field. Overall, the authors have done a fine job addressing edits, but I wish to see several other changes made to this MS.

We thank the reviewer for their thoughtful and constructive comments, which have helped us improve the manuscript. We acknowledge the importance of including as many relevant data sets as possible and have indeed continued our search following the initial submission. In response to your suggestion, we expanded our search strategy by screening articles published by authors already identified in our initial dataset. This led to the inclusion of two additional studies that had not emerged from our systematic search nor from the systematic review we used as a complementary source.

We recognize that, despite our efforts, our dataset may still not be exhaustive. We have explicitly mentioned this limitation in the revised Discussion section. Nonetheless, we believe that our analysis provides robust evidence that the distribution of the duration of IVD is not consistently exponential, and that alternative distributions such as the beta distribution may offer a better fit.

Lines 338-342: " Furthermore, we might have missed articles providing relevant data since the tools used for systematic searches of articles are not perfect. Nevertheless, the variety of experimental data that already exists - and which was used in our study – is sufficient to lead to robust conclusions, and enables us to propose a modeling framework that will be easy to use on new data once it becomes available."

We have taken into account the reviewer's comments regarding key knowledge in arbovirology, as well as their suggestions to improve the clarity of our results. These aspects are addressed in detail below.

Major comments

R2.1 : If the main purpose of this manuscript is to model how EIP is not exponentially increasing but rather unimodal then I would suggest changing the title of this MS. Additionally, as I stated in my last review I am not convinced that these findings are novel.

The objectives of this manuscript is twofold: (1) to show that EIP duration is not always exponentially distributed; and (2) to propose a way to model IVD and infer simultaneously the distributions of IVD stage durations and the probabilities of crossing intra-mosquito barriers. Our model and the inference of its parameters clearly highlight processes generally not accounted for in models of vector-borne diseases, which do not detail IVD. This is novel, and we believe it is important enough not to reduce the title of the manuscript to the exponential hypothesis. Therefore, we would like to leave the title unchanged.

R2.2: when you say selected you mean the model selected it, I think a different word would be better suited here to describe this result.

A sentence has been added to better explain what was meant and to increase clarity.

Lines 179-181: "For the infected stage, the main distribution selected (*i.e.*, the one the most frequently selected among those providing the best fit to the data) was the beta distribution for 7 out of 7 scenarios tested for DENV (Fig. 2Ai) and for 9 out of 15 scenarios tested for ZIKV (Fig. 2Bi)."

R2.3: A caveat that should be mentioned is that crossing each of these barriers within the mosquito includes a bottleneck, thus the population of the virus is changing and populations that perform well in one compartment may not be well suited for the other. I would review the literature on quasispecies dynamics
https://www.sciencedirect.com/science/article/pii/S2589004223017881
https://www.cell.com/cell-reports/fulltext/S2211-1247(17)30453-9
https://www.sciencedirect.com/science/article/pii/S1879625716301365
https://www.mdpi.com/1999-4915/6/10/3991

We agree with you that variability in virus population could affect its transmission. This is now discussed in our manuscript.

Lines 314-321: "As our inference provides probability distributions for IVD parameters rather than single point estimates, our study offers a framework to capture the inherent variability in IVD. This variability may reflect differences in vector competence between mosquito populations (35), as well as the genetic diversity and evolution of the viral population during incubation. Indeed, each intra-mosquito barrier creates a bottleneck that changes the viral population and shapes genetic diversity (36). Coupling our model with population models could help to assess how IVD variability could influence the transmission dynamics of vector-borne diseases (12,23,24)."

R2.4: it's common for the infection to go up after the initial timepoint, you should cite the studies where infection goes up.

We added references of articles used here for which this dynamic has been observed and other complementary references.

Lines 407-412: "Further investigations could focus on adding a stage between exposed and infected vectors to represent individuals infected but below the detection threshold. Indeed, in some of the experiments used here (46–48), the number of mosquitoes observed in the infected stage increased between the first and second time point studied. This has been observed in some other competence experiments (49–51), but our model does not allow such dynamics, as it assumes the infected barrier is crossed within the first few hours after the infected meal, consistent with the duration of digestion (30,31)."

R2.5: I would also mention coinfections could influence the disease dynamics within the mosquito

We now mention this point in the discussion.

Lines 434-436: “Another modification could be to consider the effect of co-infections (54,55) or multiple blood meals on IVD, with the aim of developing a more accurate representation of the natural behavior of mosquitoes (56,57).”

R2.6: stating this could be used for transmission control is a stretch, how would a public health official utilize this model in their day-to-day work?

Our sentence was not clear. We meant to say that the inclusion of our IVD model in transmission models for vector-borne diseases could improve their accuracy. We have modified the sentence to make it clearer.

Lines 443-449: “The present study proposes a generic modeling framework that enables researchers studying the influence of varying IVD characteristics on vector-host transmission dynamics. It is applicable to other arboviruses and provides a building block to improve the predictive capacity and accuracy of population-scale models. Such transmission models are one of the tools used to identify efficient control measures to fight arboviruses. Improving their accuracy would help limit the significant threat that vector-borne diseases pose to public health in many parts of the world, a threat which is increasing due to climate change.”

<u>Minor comments</u>

R2.7: rather than saying infected 2x I would say taking an infectious bloodmeal

The sentence has been modified.

Lines 177-179: “We have shown that it is often unrealistic to assume that a large proportion of mosquitoes become immediately infectious after taking an infectious blood meal, as presumed by the exponential distribution (Fig. 2).”

R2.8: should state open access -> Done, lines 279-281 : “Our study, which focuses on DENV, CHIKV and ZIKV - three major human arboviruses - took into account all open access published vector competence experiments (that we could find) with comparable protocols.”

R2.9: R0 should be written as a subscript -> Done (line 322)

R2.10: I’m not sure why Fig2C is being discussed before Fig 2A but if this is how you want it formatted then you should change the order of the panels. -> Figure has been modified.

<u>Reviewer #3:</u>

Thank you for responding and addressing reviewer comments. All my concerns have been addressed.

We sincerely thank the reviewer for taking the time to evaluate our revised manuscript and for confirming that all concerns have been addressed. We appreciate your constructive feedback throughout the review process, which has helped us improve the clarity and quality of our work.
