# Supplementary material for: Intra-vector infection dynamics challenge how to model the extrinsic incubation period for major arboviruses: dengue, Zika, and chikungunya
Source: PLoS Comput Biol. 2025 Aug 25;21(8):e1013393. doi: 10.1371/journal.pcbi.1013393 (PMC12440223; doi:10.1371/journal.pcbi.1013393)
Supplement: Attachment — Submitted filename: ReviewComments241212.docx [file pcbi.1013393.s005.docx]

In this manuscript, the authors present a stochastic model of the progress of mosquitoes through stages of viral infection, from exposure to the ability to transmit. While the authors present this model as ‘within-vector viral dynamics’, this is not actually what is modelled. There are no virus dynamics – changes in viral population over time - in the model, rather they simulate the progression of a population, or sample of mosquitoes, through infection categories.

There are three main insights from their work, derived from calibrating the model to published competence data: i) that the distribution of the EIP for DENV and ZIKV is not exponential; ii) that intra-mosquito barriers are not systematically crossed; and that intra-vector viral dynamics are influenced by biotic factors. I would highlight that none of these conclusions are particularly novel (e.g. see <https://journals.plos.org/plosone/article?id=10.1371/journal.pone.0050972>, <https://pmc.ncbi.nlm.nih.gov/articles/PMC10771300/>, <https://www.sciencedirect.com/science/article/pii/S1567134818307159> ).

**Major comments**

We would recommend that the manuscript, particularly how the modelling and results are described, be re-written so that it is much clearer to understand. We provide some specific examples of this in the minor comments to try to help with this. We also suggest that the authors think about how best to present the work in figures as these are also difficult to interpret.

It is clear from your third section (L185) that all of your parameter estimates, and selected distributions are conditional on individual experiment settings. While this is acknowledged in this section as a reason why estimates varied, I would argue that this is not a result *per se* and indeed is certainly not a novel result, but a limitation to your study in that you don’t account for any of these variations. Importantly, the one to note which stands as separate is the virus dose/ concentration of virus in the bloodmeal as this directly influences the viral dynamics as it is the amount of starting virus – this is probably a major driver of variation in your parameter estimates. I would suggest this is all really for the discussion.

The last section from L194 could be made more succinct and more clearly explained. The main statement is true for any modelling that uses data, and the explanation in this section is quite dense so it is hard to see the useful results. The language is also sometimes vague. We’ve tried to articulate how this is with specific examples in the minor comments.

It would have been good if the authors had gone the step further and shown in this piece of work the impacts of their findings for transmission by including their fitted distributions and parameter estimates in a transmission model. This may make the publication move a step further from findings that are already generally well accepted.

**Minor comments**

**Introduction**

L56: *Do movements of peoples and goods with environmental changes necessarily imply global expansion*? Suggest here that you clarify you mean expansion of the distribution of arboviral diseases/ increased geographic range, as ‘global expansion’ is ambiguous.

L61: please elaborate on what you mean by ‘*robust provisions’* and how better understanding transmission dynamics can contribute to limiting future threats by giving tangible examples in the introduction, or omit this statement. It’s easy to make these statements, less easy to justify.

L67: ‘*high accuracy and reliability of these models are crucial to guide arbovirus surveillance and implement efficient disease management*’ – the reference given with this statement is <https://pubmed.ncbi.nlm.nih.gov/24245625/> . I would argue that this reference does not provide evidence that accuracy and reliability of transmission models are crucial to guide arbovirus surveillance and control. The study covers a retrospective modelling analysis of an outbreak. I would suggest that arbovirus surveillance and control can be implemented quite well without any transmission modelling – see <https://pubmed.ncbi.nlm.nih.gov/37347767/> - what is crucial is good data and financing. In this paper, they do refer to the use of statistics. Perhaps you can just remove this statement.

L85: ‘*most transmission models do not explicitly represent IVD but provides an approximation with a single compartment whose exit rate is assumed constant and equal to the inverse of the EIP*’. While this may be true, it should be highlighted/ acknowledged here that it is probably chosen as a simplifying assumption by modellers because varying the latent period distribution is assumed not to importantly impact the answer to the question they are using the model for.

L90: ‘*the successful crossing of all three barriers by viruses in every exposed mosquitoes’* – this is not a common assumption in vector-borne disease models – usually this is accounted for by including a parameter for the host-to-mosquito transmission probability and the mosquito-to-host transmission probability.

L107: You refer to three models of within-mosquito viral dynamics, outlining a gap in the research that there is no full model through to salivary gland infection, but in fact your model does not fill this gap as it is not a model of within-mosquito viral dynamics – you do not explicitly model viral dynamics within individual mosquitoes. I think you just need to re-phrase how you describe your modelling here.

**Results**

It would be useful to present the table of the fitted model details in the main manuscript. In the figure legends, please provide descriptions that stand alone without having to refer to other parts of the manuscript, so that someone can look at the figures and understand them without too much effort.

L133 What do you mean by ‘best’ – can you state more explicitly, especially given the methods are presented at the end, how the distribution was selected.

L134 Rather than ‘much more frequently’ can you just state quantitatively instead?

L133 – 141 is generally poorly phrased and is very repetitive. Can you re-phrase in a way that is more biologically relevant? For example, what I think you are trying to say is that it is not realistic to expect some mosquitoes to immediately become infected/ infectious. I think you can state this much more succinctly and just refer to the figure?

**Fig 1. All the data in here should stand alone so it can be interpreted without referring to the main text or supplementary material (this also goes for the other figure)**

Spell out abbreviations, and what does ‘scenario’ mean?

‘Selected proportion of model’ should be re-phrased…

1i) – it is hard to interpret – what do the colours correspond to? It says one colour per scenario – but grey is used for five scenarios? It would also be useful to be clear what you mean by scenario – here are you referring to a single vector competence experiment? If so just call it experiment or data set as’ scenario’ is vague.

Please state what a selected scenario is in this legend

iii) What does ‘effectif’ mean?

L163: Please can you explain what greater insight your model provides concerning the evidence that the barriers are not systematically crossed, beyond what is already in the data? If by the end of an experiment, not all mosquitoes have become infected/ are able to transmit – that seems self-evident? Or perhaps I am misunderstanding.

**Fig 2.**

‘values of the crossing barrier parameters’ – would suggest to re-phrase so that it is clearer e.g. something like ‘Parameter value estimates for the probability of crossing the infection and dissemination barriers’ ?

Yaxis label modegammaD – please just state ‘parameter estimate for (parameter symbol)’ etc without the ‘mode’, again instead of ‘scenario’ explain explicitly what that is -.e.g dataset or experiment.

L194: This statement seems overly obvious and is applicable to all modelling exercises that use data for calibration or fitting.

L196: ‘*the quality of our model inference was* ***satisfying*** *based on an* ***adequate*** *visual fit between observed and simulated data for the* ***selected*** *models’… ‘****satisfactory*** *visual fit* ***quality****’*. Please try and reduce vague statements.

L205: ‘*a decrease in the fit quality was observed when the dynamics of the infected, disseminated and transmitter states deviated from a ‘classic’ dynamics (i.e. increasing in the infected state, increasing and then decreasing in the disseminated state, and increasing in the transmitter state’ –* what is **classic** dynamics? Use of parentheses generally indicates poorly phrased sentence and what exactly is increasing and decreasing here?

L213: What do you mean by ‘*up to date assumptions’*?

L219: what do you mean here by low uncertainty – can you be quantitative…. A narrow 90% CI – how narrow?! And on only half of scenarios? I am really not sure what is being communicated here in these next few sentences or why.

**Methods**

I think it would help in this section if you avoided using so many parentheses. For example, ‘*To infer the distribution of the IVD stage durations, we divided respectively the 𝐼 and 𝐷 compartments into n and m sub-compartments of 1-day duration each (n and m thus being the maximum length of stay, in days, in 𝐼 and 𝐷).*’ Could be written as ‘To model the distributions of the time to dissemination and time to transmission, we divided the infection and dissemination compartments into *m* and *n* sub-compartments. The number of *m* sub-compartments was equivalent to the duration of the experiment and *n* was equivalent to one minus the duration.

L405 – If the model consisted of one compartment per IVD stage, and went on to mention four compartments (E, I, D, and T), why were three stages of IVD mentioned in L402? It is unclear

L425 – “When crossing a barrier …” consider starting this sentence in a new paragraph

L424 – “mosquitoes were distributed among the sub-compartments …”. Were they randomly distributed based on the output of multinomial distribution? If yes, make it clear by adding the word.

L433 – First it is unclear how N_tot_ relates with other compartments. Would be good to include it in model diagram or describe it in the caption. Secondly, it is mentioned that mosquito size varies between observation dates and was adapted based on experiment data in each scenario. But how this was done in the simulation (e.g., initial population size or adjusted over time) could be clarified.

L478 – Consider mentioning the acceptance criteria (e.g., threshold(s) used) and the number of accepted particles.

L479 – “providing that all four criteria were met” in what way? Do you mean if sum of squares (a single value) is less than the final threshold specified? If yes, then the sentence is a bit confusing with the phrase “all four criteria”. If you were referring to something different, would be good to make it clear.

**Discussion**

L246: ‘…*do not hold in most situations with regards to the mosquito and virus genotype and or infectious dose’* – I don’t understand how you can make this conclusion as you do not account for these factors in your modelling – do you mean irrespective of these factors? Even then, actually, at very high virus dose, these assumptions may hold, I would argue.

L253: with respect to parameterising transmission models from experimental data – this accounting for nuances in distributions may only be relevant if asking relative questions e.g. comparing between vector species because data from laboratory experiments often do not reflect ‘true’ values. For example, we know that the probability of infection is higher and the EIP shorter when mosquitoes feed on a host rather than artificial bloodmeal.

L261 – this simply isn’t true, usually this is accounted for using transmission probability parameters.
