# Supplementary material for: Intra-vector infection dynamics challenge how to model the extrinsic incubation period for major arboviruses: dengue, Zika, and chikungunya
Source: PLoS Comput Biol. 2025 Aug 25;21(8):e1013393. doi: 10.1371/journal.pcbi.1013393 (PMC12440223; doi:10.1371/journal.pcbi.1013393)
Supplement: Attachment — Submitted filename: review.docx [file pcbi.1013393.s008.docx]

As per one of the other reviewer’s previous comments, if you are going to have the results before the methods, I think you need to give a brief description of the model in the first paragraph and refer to the conceptual figure in the text.

Subsection on lines 203-205 – I still think this is so obvious that it doesn’t require a separate subsection. You talk about uncertainty in the previous section and suggest you just merge this section. I think it will help too if you give a description at the start of the results about what parameters were estimated and which relate to distributions and which relate to probability of crossing. Why does this second section not refer to the specific viruses in the same way the previous section does?

The manuscript still needs a careful re-read by all co-authors and editing to ensure language is of high quality, that sentences make sense and that results are clearly explained. This will also help with making the paper more accessible to experimental biologists and not just modellers. I’ve tried to highlight this in the Introduction and Results with minor comments below, but please note that I have not done this for the Methods and Discussion – it should really be the responsibility of co-authors to do this job.

72: you mention competence here, but the EIP is a component of competence – also is there are relationship between vector competence and the EIP? Would advise to -re-phrase this sentence. Perhaps start by defining competence and that EIP is a component of it.

76: midgut not intestine

85 – 87: I do think here you need to give some credit to modellers who often will use this simplification after critically assessing the impact it will have on results. I would suggest that it is the case that for any given model designed to address a specific question, that often this simplification is acceptable without it compromising the validity of the results. It might just be a case of adding a sentence that in many cases it is likely acceptable.

91: rather than non-exponential just state the distribution that better represents?

96: not sure what you mean by ‘offsetting any potential impact on transmission’?

107: integrating a wider range of experimental conditions – not sure what you mean here – into mathematical models? Do you mean enable more flexibility in how we model vector competence? I think an example would be useful here to make it clearer. If by experimental conditions you mean, for example, variation in temperature – this still can be incorporated using an exponentially distributed EIP.

121: stage

131: rephase subtitle? Perhaps - The EIP of ZIKV and DENV is not exponentially distributed

Fig 1: 145-146 ‘vector with virus in its digestive system into blood host’ makes no sense, need to rephrase…. Midgut not intestine. Italicise mathematical symbols.

159 missing a closing bracket.

166: ‘was selected’ – provided a better fit to the data.

Are lines 165 – 168 and 168 – 171 not saying the same thing? Perhaps delete 165 – 168?

173: what do you mean by ‘partial counterpart’. What does SEID stand for – you need to spell this out in the main text on first use.

176 – 177: of course ‘low uncertainty’ relates to ‘high precision’ they are the same thing? Either explain this quantitatively here or refer to a figure or table of results? How do you define ‘low’?

176 – 187: when you are talking about comparison between distributions – is this between model fits to data that have the same experimental conditions on the same virus-vector combination? Or a single set of data and across the stochastic model outputs? I think this section just needs a little clearer explanation – it’s quite dense.

193: you need to spell out these abbreviations.

194: do you mean datasets rather than scenarios – be clear what you are referring to…. Ah I’ve just seen this on 199 – you can’t have this at the end of a figure caption – you need this definition upfront at the start of the results to avoid the reader being confused!

210: models

256-259: re-phrase these two sentences so it is clearer and remove ‘fit quality reduced in scenarios where the observed dynamics deviates from the expected dynamics’ – because that IS what fit is?! ‘which is first a decrease in the infected state, followed by an increase and subsequent decrease in the disseminated state, and finally a continuous increase in the transmitter state.’ – I have no idea what this means. You can’t have an increase in a ‘state’ – what does this mean?

263 – 266: This is for the discussion but now I see what you were trying to articulate in the previous sentences. I think you need to start with a clear description of this at the beginning of this section – essentially the data suggest that some infected mosquitoes can become uninfected? Make sure to elaborate in the discussion any evidence that this is biologically feasible (e.g. immune clearance) or could be due to diagnostic sensitivity or some other laboratory-related reason.

271: proficiency?! You been competence?
